# Supplementary material for: Hemispherotomy leads to persistent sleep-like slow waves in the isolated cortex of awake humans
Source: PLoS Biol. 2025 Oct 16;23(10):e3003060. doi: 10.1371/journal.pbio.3003060 (PMC12530565; doi:10.1371/journal.pbio.3003060)
Supplement: S1 Appendix — Table A. Demographic information for each patient that underwent cortical disconnection of a single entire hemisphere (hemispherotomy), or of the parieto-occipito-temporal area (hereby noted as posterior), lateralized to one hemisphere. SA—Exclusion of EEG segments due to epileptic activity. Fig A. Excluded or included data segments due to epileptic activity. SB—The isolated cortex shows an increase in total power, in the context of a broad-band power redistribution. Fig B. Surgical disconnection increases overall total EEG power in the isolated hemisphere with respect to the contralateral hemisphere after surgery and to the state of the isolated cortex prior surgery. SC—Fitting of the Spectral Exponent and supplementary results. SC.1—Fitting of the Spectral Exponent. Fig C. Residuals/Periodic Component and Proportion of channels excluded from fit. Fig D. Group-level broad-band PSD and aperiodic trend in hemispherotomy, estimated over a broader 0.5–40 Hz range. SC.2—Results in the patient dataset of the Spectral Exponent, estimated between 1 and 20 Hz. Fig E. Replication of the findings of Fig 2 in the patient dataset on the Spectral Exponent, estimated over the 1–20 Hz range. SD—Mixed effects models. Table B. ANOVA of mixed effect models reveal a significant interaction between time and spatial side, indexing that the surgical disconnection differently affected each hemisphere. Table C. Pairwise Contrasts across spatial and temporal conditions on EEG slowing features. SE—Comparing narrow-band versus broad-band slowing in cortical isolation. Fig F. Bootstrap analysis revealed that the Spectral Exponent showed stronger statistical effects than Slow Delta Power in detecting the electrophysiological effect of cortical isolation on the disconnected hemisphere. SF—Controlling for Etiology and Type of Hemispherotomy. SF.1—Etiology. SF.2—Type of hemispherotomy. Fig G. Narrow- and broad-band spectral changes after surgery reveal a marked EEG slowing of the isolated cortex, [file pbio.3003060.s001.docx]

# **Appendix Supplementary Material related to “Hemispherotomy leads to persistent sleep-like slow waves in the isolated cortex of awake humans”**

| **Patient  Num** | **Surgery  Side** | **Surgical area** | **Etiology** | **Age (y)** | **Months to Follow-up** | **sex** |
| --- | --- | --- | --- | --- | --- | --- |
| 1 | R | posterior | malformation | 11,83 | 30 | M |
| 2 | L | hemisphere | ischemic | 6,50 | 7 | M |
| 3 | L | hemisphere | ischemic | 8,17 | 13 | F |
| 4 | R | hemisphere | malformation | 7,08 | 35 | M |
| 5 | R | posterior | malformation | 2,58 | 16 | M |
| 6 | R | posterior | ischemic | 10,17 | 20 | F |
| 7 | R | hemisphere | malformation | 10,67 | 19 | M |
| 8 | L | hemisphere | ischemic | 5,00 | 31 | M |
| 9 | R | hemisphere | ischemic | 8,33 | 8 | F |
| 10 | L | hemisphere | ischemic | 11,00 | 20 | F |

**Supplementary Table A**. **Demographic information for each patient that underwent cortical disconnection of a single entire hemisphere (hemispherotomy), or of the parieto-occipito-temporal area (hereby noted as posterior), lateralized to one hemisphere**. The underlying etiology was either ischemic or a congenital malformation. L stands for Left, R for Right, M for male, F for Female.

**SA - Exclusion of EEG segments due to epileptic activity**

Below, in the upcoming 5 pages, we display representative EEG traces for segments that were excluded or included due to the presence (or absence, respectively) of epileptic activity (similarly to Figure 2 of Avvenuti et al., 2020), as marked by a trained neurologist (**Supplementary Figure A)**.
Epileptiform activity in the isolated cortex was found to be dependent on the nature of the underlying tissue. In patients with extensive cortical malformations (such as cortical dysplasias and polymicrogyria), frequent high-amplitude epileptiform discharges, including spike/polyspike-wave complexes and sharp wave-slow wave complexes were observed. In contrast, in patients with post-stroke sequelae, isolated spikes or sharp waves occurred against a background of low-to-medium voltage activity. In both cases, epileptiform discharges are organized into bursts separated by periods of slow wave activity (see **Supplementary Figure A** for examples).

**Note on section labels**

The Appendix sections were converted from numerical to alphabetical labels (1=A, 2=B,…) according to editorial rules. Readers interested in reading the revision history, or in downloading the excel files reporting the numerical values displayed in the figures (provided in the Zenodo online repository), they may consider this conversion system to locate the appropriate figures and sections. The first author wishes to thank Dr. Grade Tardi for providing a role model of endurance and persistence during the monotonous conversion process.

**
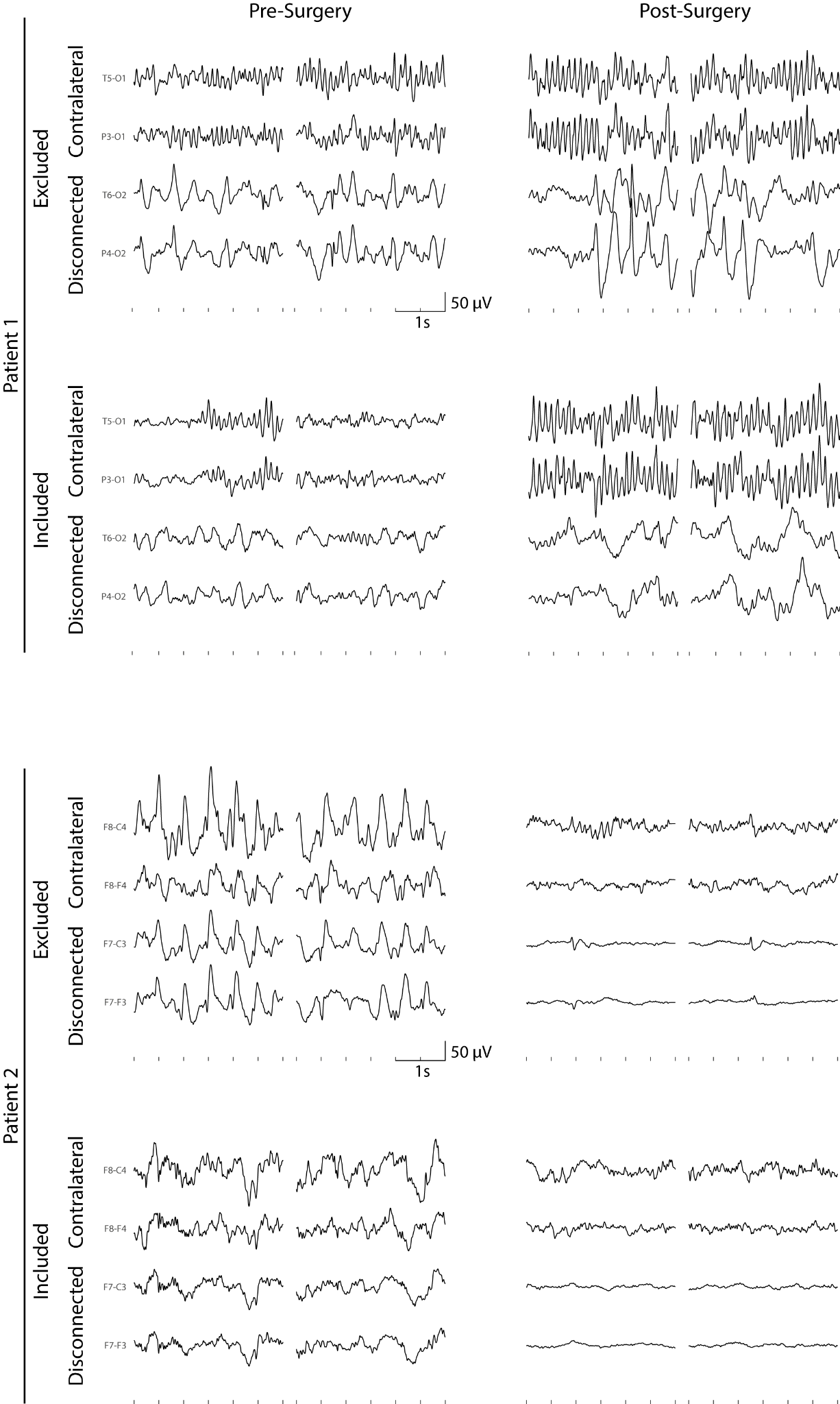
**

**
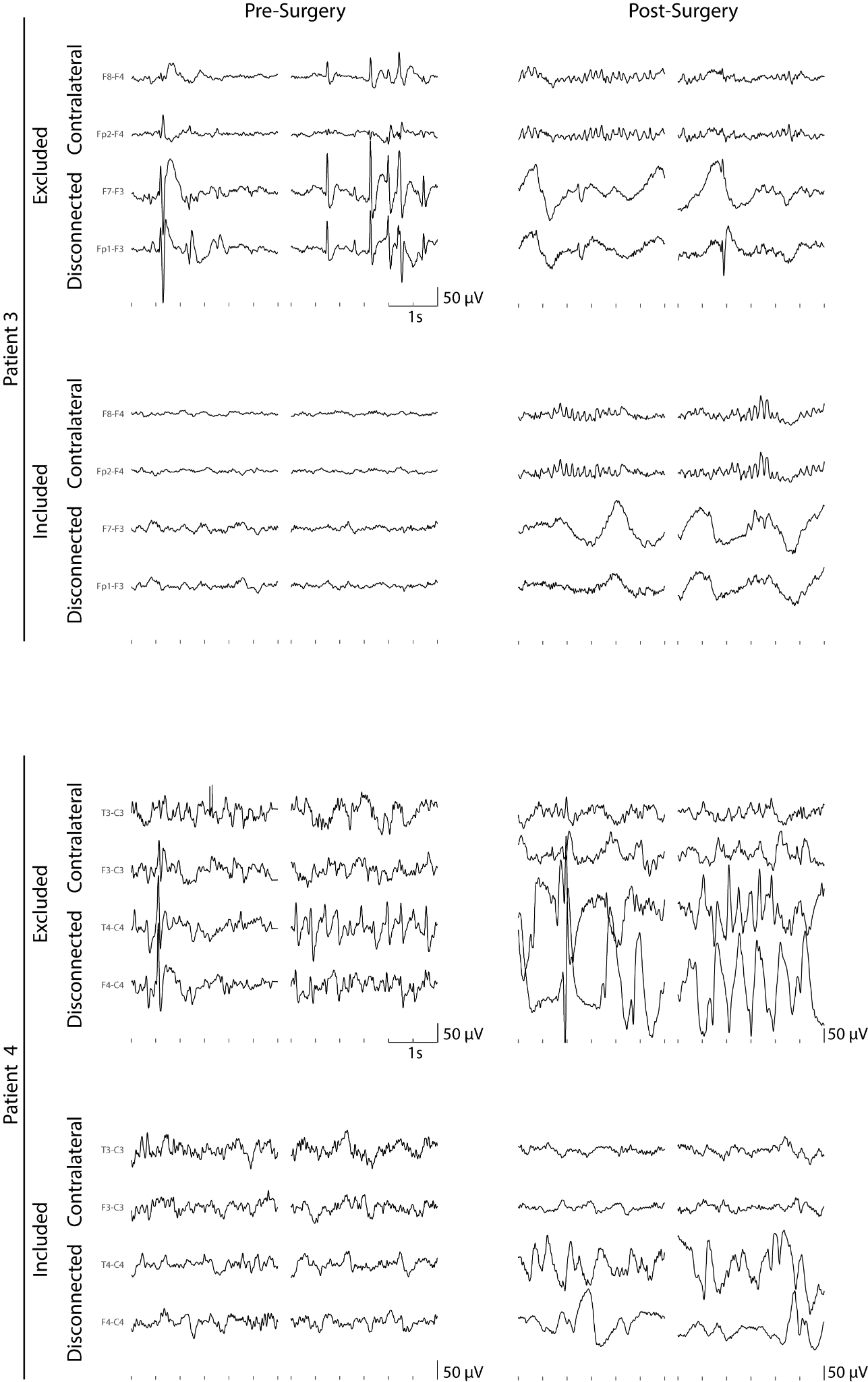
**

**
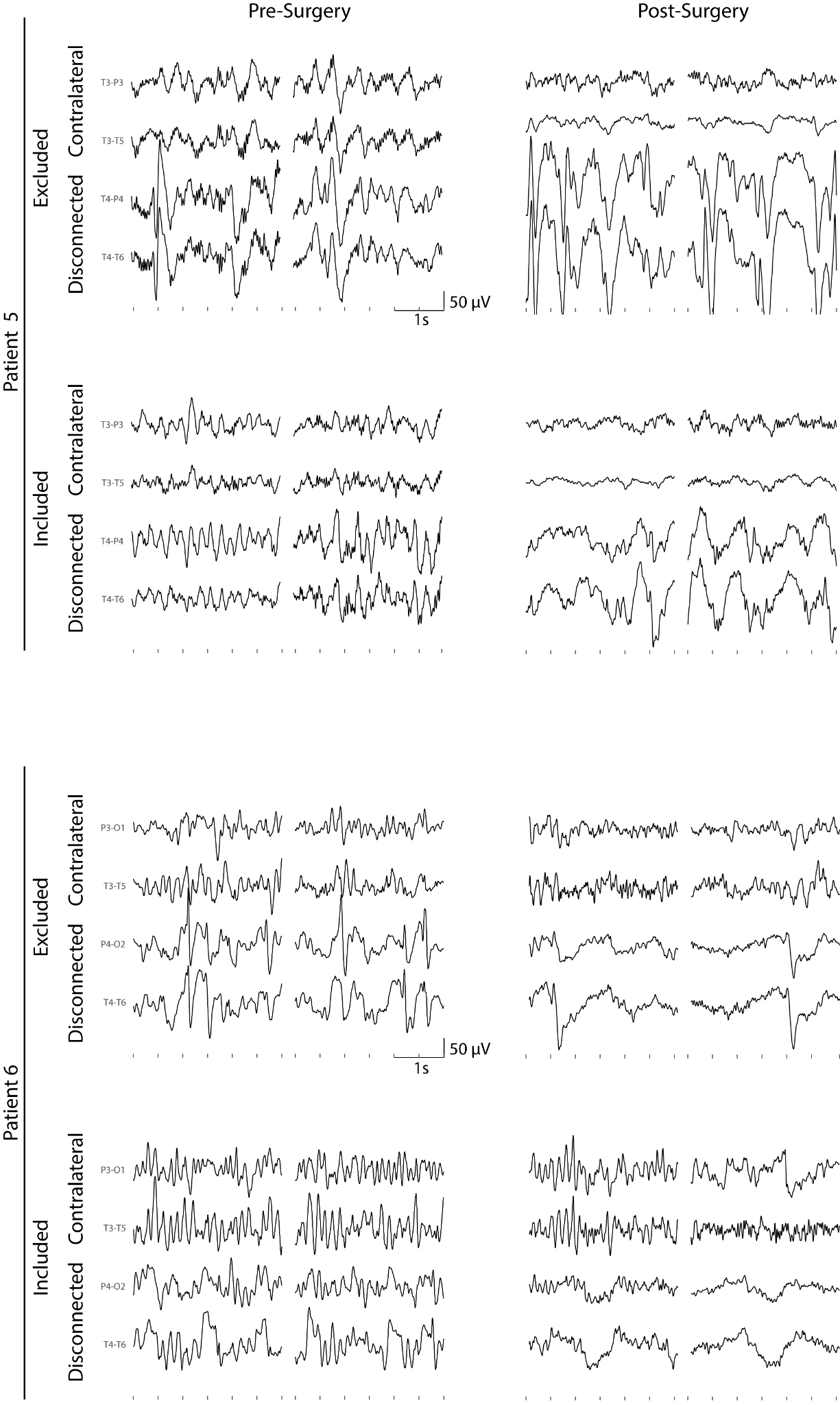
**

**
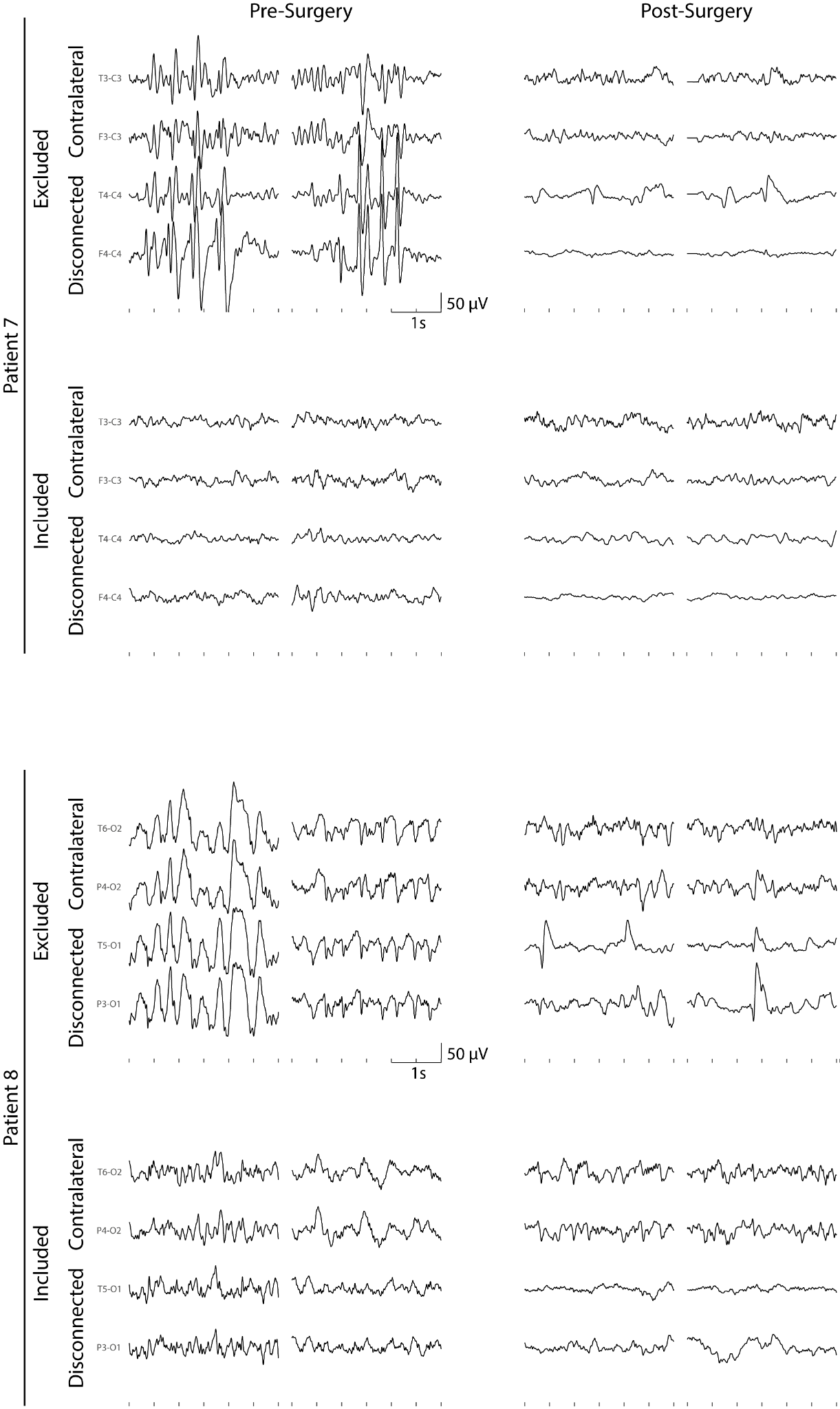
**

**
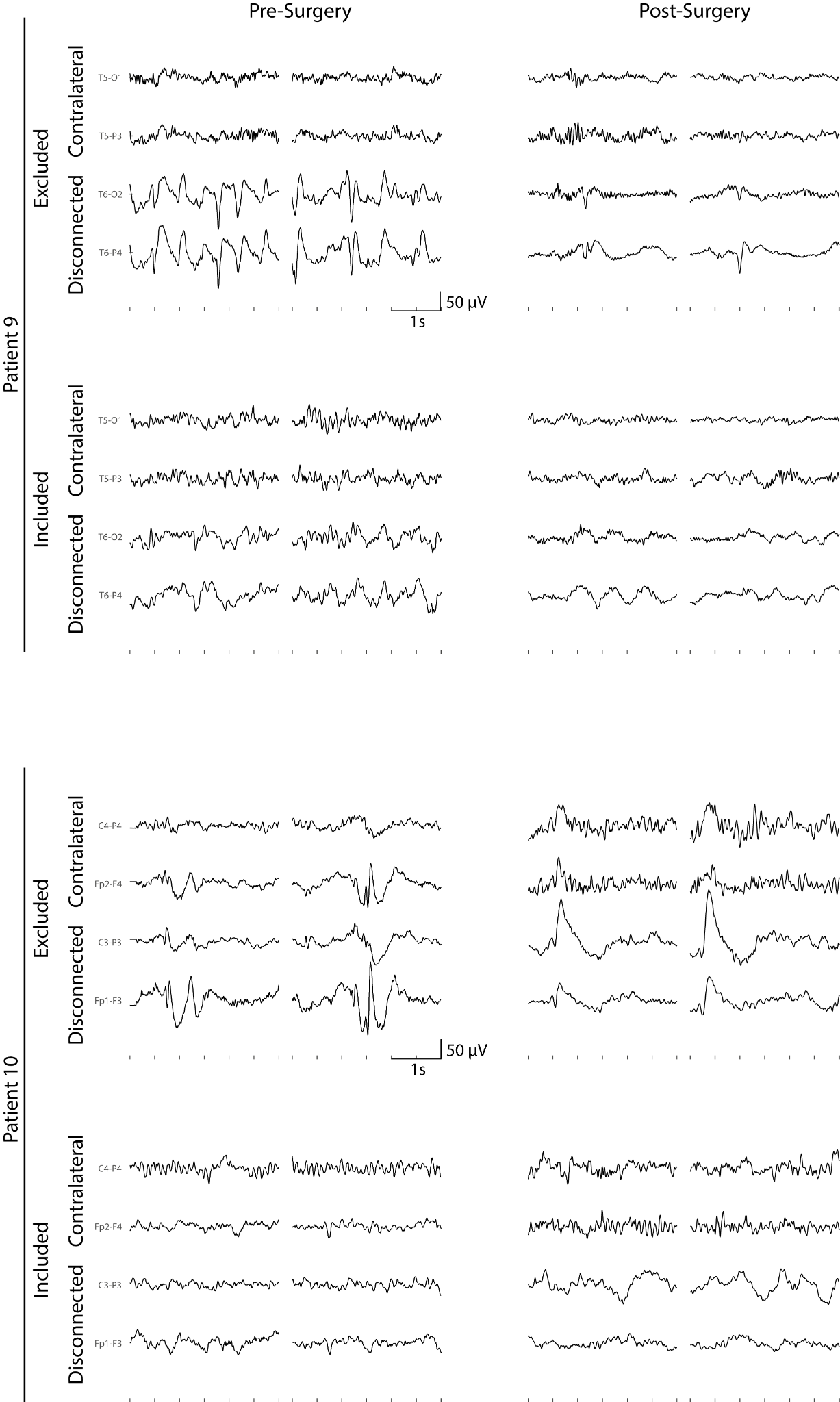
**

**Supplementary Figure A. Excluded or included data segments due to epileptic activity**

We display representative EEG traces for segments that were excluded or included due to the presence (or absence, respectively) of epileptic activity, as marked by a trained neurologist. Each page of the previous five pages displays two patients. For each patient, in each session (pre- and post-surgery), for both included and excluded epochs, we display two representative segments (of 3 seconds each, the same length used for each periodogram in the PSD estimation). Each segment displays activity from two bipolar contacts in the disconnected cortex (chosen individually for each patient to highlight epileptiform activity) and two in the contralateral side (the corresponding homologous contralateral contacts). By only retaining segments that do not display epileptic graphoelements, we minimized the chance that epileptic activity might constitute a bias for the observed spectral changes following hemispherotomy.

**SB - The isolated cortex shows an increase in total power, in the context of a broad-band power redistribution**

While the results in the patient dataset for the narrow-band analysis of the PSD (in the Slow Delta band) are presented in the main text, we here report the results on total absolute power (i.e., the sum of the power spectral density across all frequencies). Regarding spatial inter-hemispheric contrasts (contralateral vs. disconnected hemisphere), before surgery there was no significant difference in total power between the disconnected cortex and the contralateral cortex (T(9) = 0.828, p = 0.4293, **Supplementary Figure B, left panel**). After surgery, a significant difference in total power emerged between the disconnected cortex and the contralateral cortex (T(9) = –2.917, p = 0.0171, **Supplementary Figure B, middle panel**). Regarding temporal contrasts (Difference Post-Pre surgery), total power increased post-surgery relative to pre-surgery within the disconnected cortex (T(9) = 2.577, p = 0.0298), but not within the contralateral cortex (T(9) = 0.541, p = 0.6019), **Supplementary Figure B, right panel**.


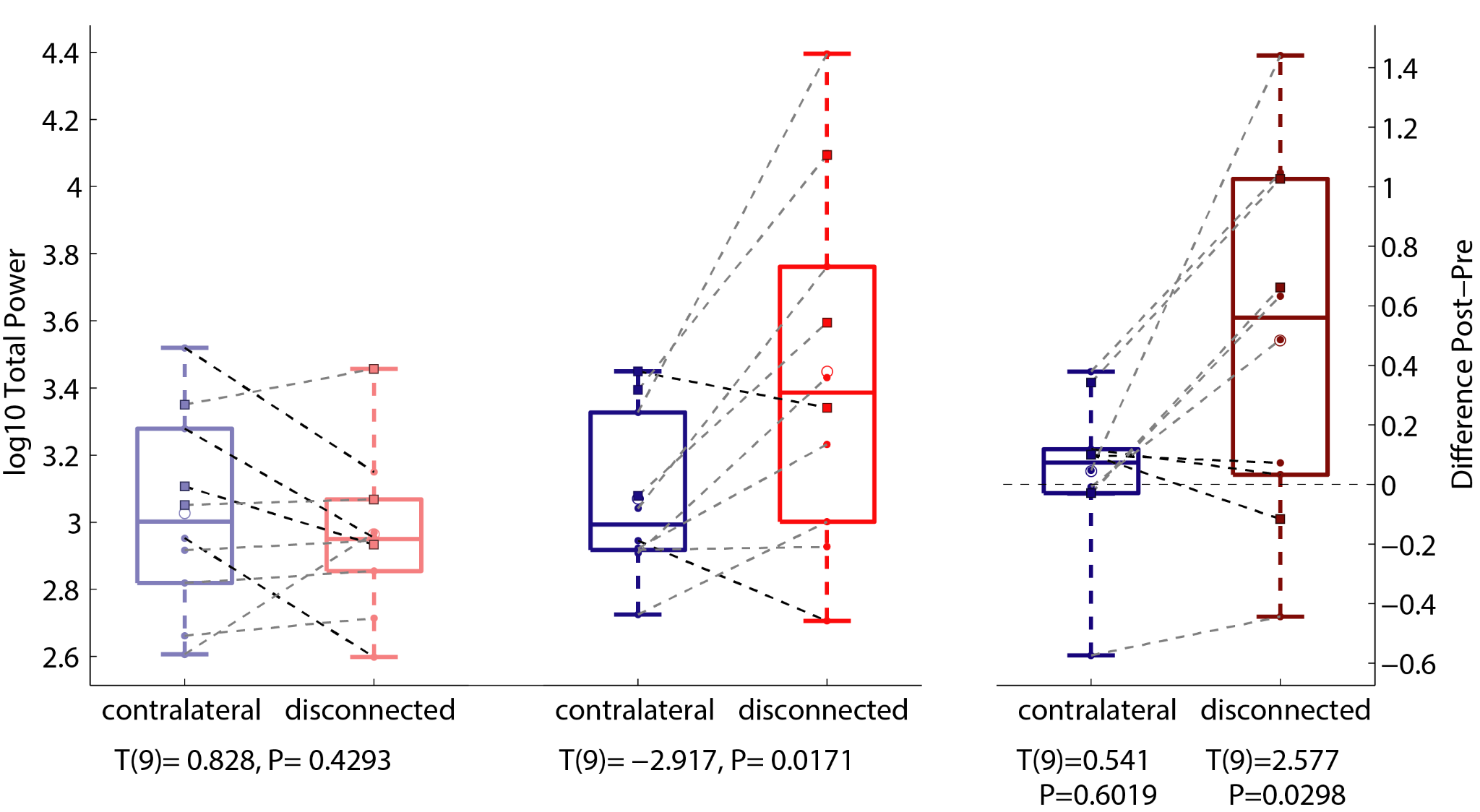


**Supplementary Figure B. Surgical disconnection increases overall total EEG power in the isolated hemisphere with respect to the contralateral hemisphere after surgery and to the state of the isolated cortex prior surgery.** Total absolute EEG power in the disconnected and contralateral cortex before and after surgery. Boxplots show total power values for the disconnected cortex (light red: pre-surgery; red: post-surgery) and the contralateral cortex (light blue: pre-surgery; blue: post-surgery).

Left panel: Before surgery, total power did not differ significantly between the two hemispheres. Middle panel: After surgery, total power was significantly higher in the disconnected cortex compared to the contralateral side. Right panel: Change in total power from pre- to post-surgery. A significant increase was observed in the disconnected cortex (red boxplot), but not in the contralateral cortex (blue boxplot). Patients who underwent complete hemispherotomy were marked with a dot, while those who underwent temporo-parieto-occipital disconnection were marked with a square. The data underlying this figure are provided in the file FigS2_Data, publicly available in the repository at the following <https://doi.org/10.5281/zenodo.16936516>

The disconnected cortex showed a robust increase in power, primarily due to low-frequency activity; this effect remained statistically significant even when considering total absolute power across all frequencies (see **Supplementary Figure B**). This increase in power was most pronounced in the Slow Delta band (0.5–2 Hz), as shown in **Figures 1 and 2**. However, the rise in Slow Delta power was not only an isolated narrow-band effect; rather, it occurred in the context of a broad-band spectral redistribution. The slope of the PSD increased, as well as its offset (Figure 2, inset of panel A), according to an overall clockwise rotation of the PSD profile. Specifically, while the spectral offset can change independently of the slope (reflecting a vertical shift in the PSD), any change in the spectral exponent necessarily leads to a change in offset when the PSD rotates around a pivot point at a non-zero frequency (for a visual explanation see: <https://github.com/fooof-tools/Visualizers#spectral-rotation>; see also **section SC**, and Favaro et al., 2023, for a discussion). This rotation reflects a global steepening of the PSD and a redistribution of power from higher to lower frequencies, quantified by a more negative spectral exponent (**Figure 2D**). Notably, changes in the spectral exponent showed stronger statistical evidence than changes in Slow Delta power (see **Supplementary Figure F**). These joint spectral changes point to a single underlying transformation in cortical dynamics.

**SC - Fitting of the spectral exponent and supplementary results**

**SC.1 - Fitting of the spectral exponent**Our fitting algorithm first resamples the PSD over log-spaced frequency bins (upsampling to ensure that the lower frequencies are adequately represented and leveraged with respect to the higher frequencies), then performs a preliminary linear fit (in log-log axes). Subsequently it identifies peaks (large residuals from the preliminary fit, > 1 median absolute deviation, also displaying a peak/inflection point) along with the base of the peaks (frequency bins with positive residuals adjacent to peaks).

In order to better estimate the aperiodic component, peaks and their base are subsequently excluded from a second power-law fit. The proportion of electrodes whereby a frequency bin was excluded, on average across patients, is displayed at the bottom of **Supplementary Figure C.** The residuals/deviations from the second power-law fit of the PSD, averaged across electrodes and patients via geometric mean (in line with the main **Figure 1C**) correspond to the estimated periodic component of the PSD (top of **Supplementary Figure C).** PSD residuals, after the removal of the aperiodic component (1/f-like trend), display peaks in the typical delta, theta, alpha and beta band, corresponding to periodic activity.


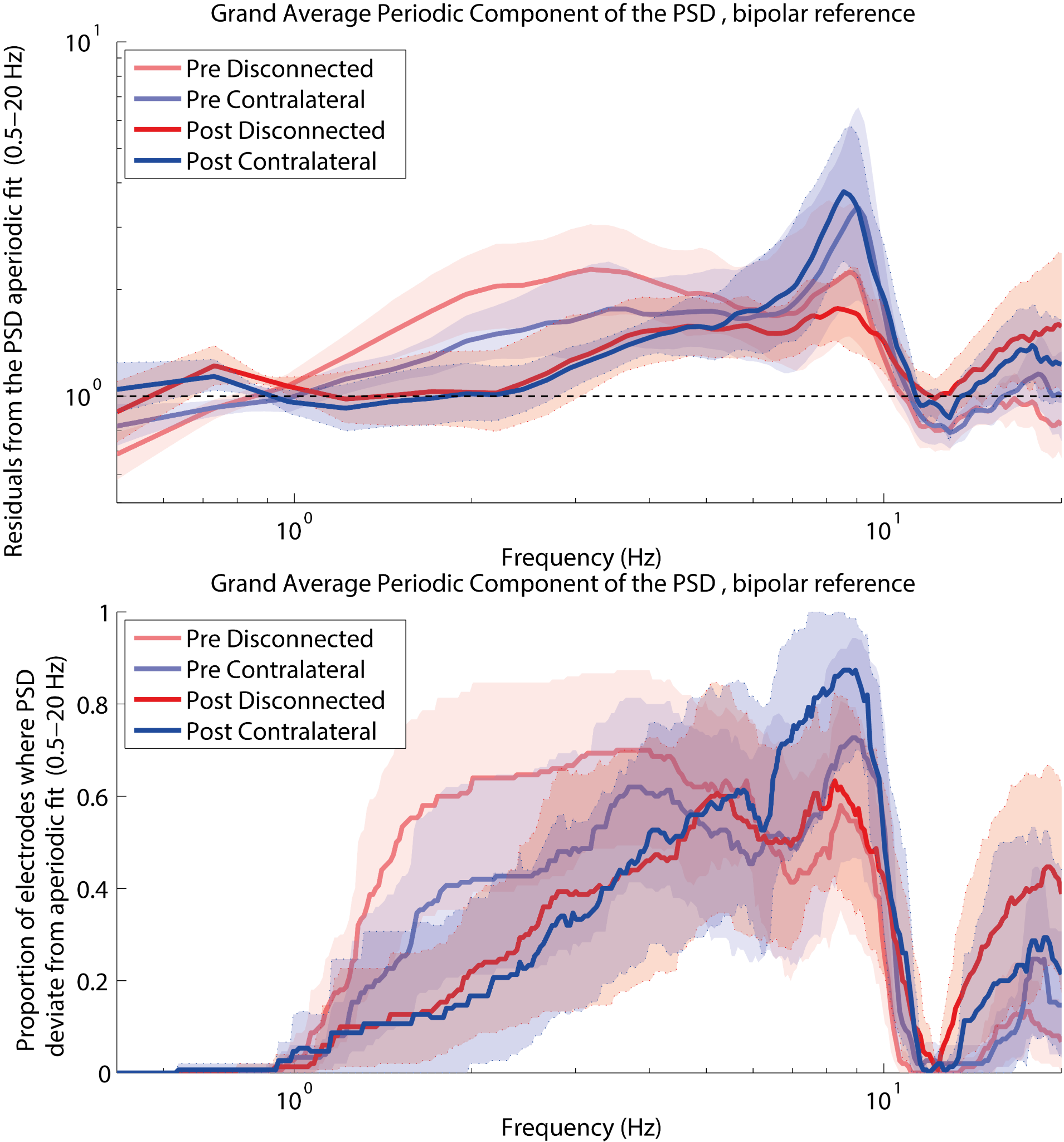


**Supplementary Figure C. Residuals/Periodic Component and Proportion of channels excluded from fit.** Inspection of the periodic component of the PSD, as well as of the proportion of electrodes across frequencies displaying peaks highlights the presence of delta, theta, and beta oscillatory activity. Sigma peaks, indexing a thalamocortical synchronization phenomenon typical of NREM sleep, could not be observed. See also **Supplementary Figure R** for an in-depth analysis of total and periodic sigma PSD in the patient and in the reference dataset.

The identified PSD trend corresponds to the aperiodic 1/f component (displayed in **Figure 1C**, and **Figure 2A**). This aperiodic component of the PSD is thus parameterized by two features: the slope (spectral exponent) and the intercept (spectral offset). While the slope reflects how power decreases with increasing frequency, the intercept captures the overall vertical shift of the PSD—providing an estimate of broadband power that is independent of specific oscillatory peaks and reflects the overall energy of the aperiodic activity across frequencies. Details of the procedure have been published in Colombo et al., 2019 and the Matlab code (and equivalent Python code) is available online at: <https://github.com/milecombo/spectralExponent/blob/master/README.md>.

The PSD aperiodic component consistently decays over frequencies, approximately according to an inverse power-law. Such decay can be characterized either by a negative spectral exponent value (as in Colombo et al., 2019; Colombo et al., 2023; Gao, 2016; Lendner et al., 2020), as we do in this study, or equivalently, by a positive value (Cellier et al., 2021; Schaworonkow and Voytek, 2021; Favaro et al., 2023), according to the formula used for fitting and depending on the use of the absolute value.

While the main text focuses on spectral trends occurring below 20 Hz, we present below in the **Supplementary Figure D** a broader frequency range (0.5-40 Hz) to show that the spectral decay continues above 20 Hz.


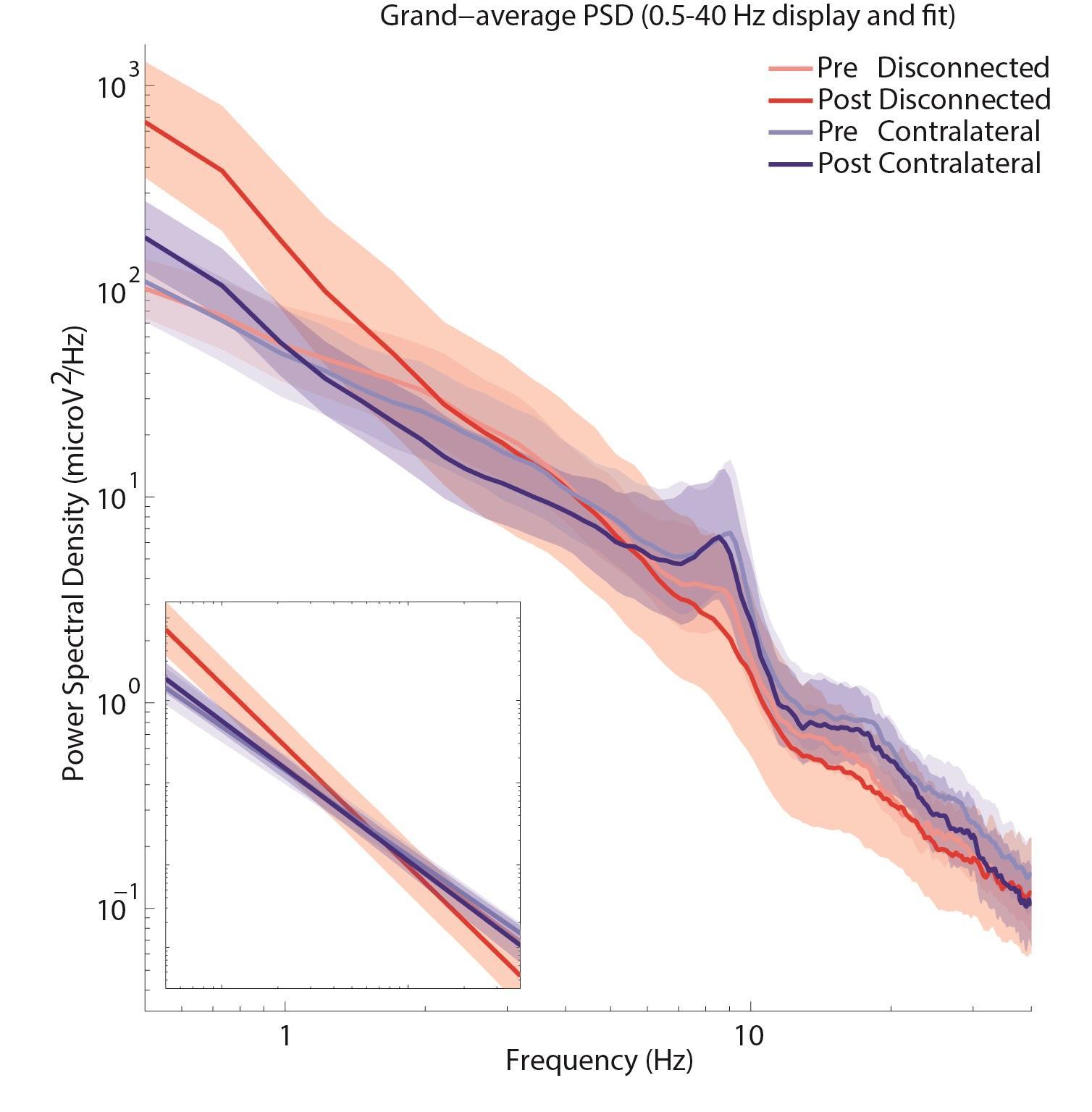

**Supplementary Figure D. Group-level broad-band PSD and aperiodic trend in hemispherotomy, estimated over a broader 0.5-40 Hz range.** The geometric mean of the PSD was taken first across electrodes, and subsequently across patients, for the observed PSD (main graph) as well as for the aperiodic fit of the PSD estimated in the 0.5-40 Hz range (corresponding to a straight line under logarithmic scale for both x and y axes, inset graph). The shaded area indicates the bootstrapped 95% confidence interval for the geometric mean across participants. This graph illustrates a broader frequency range to contextualize **Figure 2A** and shows that the broad-band spectral decay continues in the higher frequency range, above 20 Hz, in all conditions.

**SC.2 - Results in the patient dataset of the Spectral Exponent, estimated between 1-20 Hz**

In order to compare spectral exponent values across datasets, we considered a narrower frequency range than in the main section (**Figure 1, 2, 3**), and replicated the main findings over this frequency range in the patient dataset.

As mentioned in the Methods section of the main text, to directly compare spectral exponent values across datasets, we aligned the preprocessing pipeline of the patients with cortical disconnection to that of the previously published study on the reference pediatric sample (Favaro et al., 2023). Hence, in the patients’ dataset, we increased the Butterworth high-pass filter from 0.1 to 0.5 Hz, and consequently restricted the fitting range of the spectral exponent from 0.5-20 Hz to 1-20 Hz (i.e. with the lower bound set at a frequency where the filter effects were dissipated and the expected PSD decay was observed), according to the procedure used in the reference dataset (Favaro et al., 2023).

While in the main text we report the contrasts within the patient dataset on the spectral exponent estimated between 0.5-20 Hz (**Figure 1, 2, 3**), we here report the contrasts on the spectral exponent estimated between 1-20 Hz, after raising the cut-off frequency of the high-pass filter from 0.1 Hz to 0.5 Hz.

As shown in **Supplementary Figure E,** we observed a mild inter-hemispheric asymmetry before surgery, a marked inter-hemispheric asymmetry after surgery, and a decrease after surgery of the spectral exponent (1-20 Hz) to more negative values, localized in the disconnected cortex. Thus, even after restricting the frequency range under study (from 0.5-20 Hz to 1-20 Hz), all findings on the spectral exponent were consistently replicated in the patient dataset.


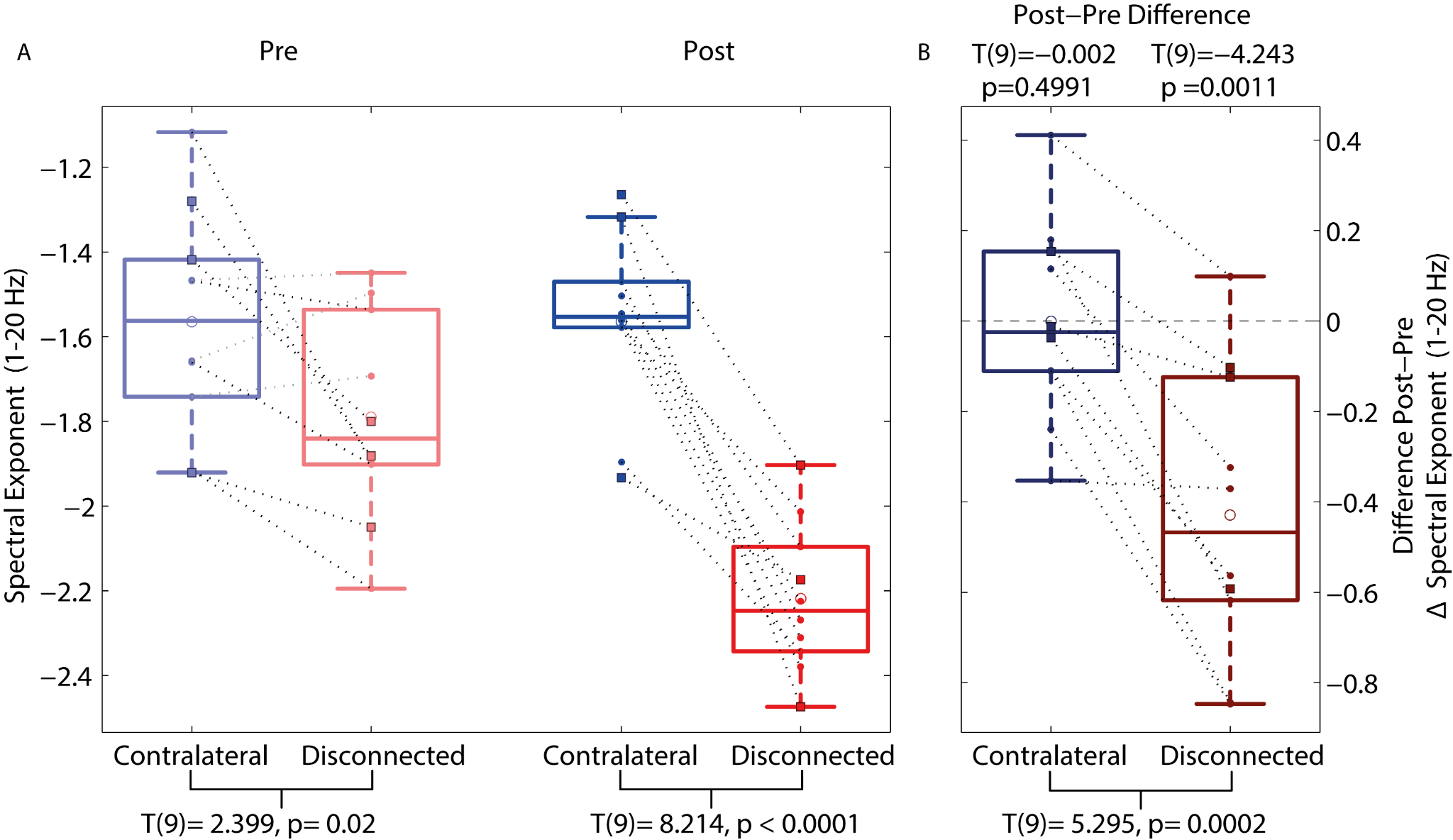


**Supplementary Figure E**. **Replication of the findings of Figure 2 in the patient dataset on the spectral exponent, estimated over the 1-20 Hz range.** A) The spectral exponent (1-20 Hz) was significantly more negative in the disconnected than in the contralateral cortex, in the session before surgery (Pre) and, particularly, in the session after surgery (Post), whereby consistent inter-hemispheric asymmetry was observed in all patients (statistics for the contrast between sites are shown at the bottom of the graph, separately for the pre- and post-session). B) Following surgery, the PSD became steeper in the disconnected cortex; as indexed by a significant pre-post decrease of the spectral exponent, observed in all patients (Additional statistics to contrast the difference against 0 are shown at the top of the graph, separately for each side). Patients who received a temporo-parieto-occipital disconnection (squares with a black outline) displayed an overall similar pattern to that of patients who received a hemispherotomy (small dots). The data underlying this figure are provided in the file FigS5_Data, publicly available in the repository at the following <https://doi.org/10.5281/zenodo.16936516>

**SD - Mixed effects models**First, two mixed effect models were built to assess whether each univariate EEG marker (spectral exponent and Slow Delta power) differed across spatial side (Disconnected and Contralateral side), temporal sessions (Pre and Post surgical disconnection) or due to possible spatio-temporal interaction effects, while controlling for the nesting of the spatial and temporal variables within participants. Specifically, each univariate marker was considered as a response variable, the spatial side, the temporal session as well as their interaction were entered as fixed effects, while spatial side and temporal session, grouped by subject, were entered as random effects.

Thus, the resulting formula was:

*EEG feature~ SpatialSide + Time + SpatialSide*Time + (1 + SpatialSide + Time | Subject)*F-tests were performed for each fixed-effects term, by means of a type-3 ANOVA for the linear mixed-effects model.

According to the ANOVA on the mixed effect models (**Supplementary Table B**), the Slow Delta power showed no significant effect of spatial side, a significant effect of time, and, importantly, a large, significant effect of the interaction between spatial side and time.

The spectral exponent showed a significant effect of spatial side, a large, significant effect of time, and, importantly, a large, significant effect of the interaction between spatial side and time.

The results from the mixed effects models pointed to a change in the degree of narrow- and broad-band slowing following surgical disconnection that differently affected each hemisphere. Thus, the statistical pattern was further explored by means of a series of pairwise contrasts across conditions (paired t-tests), under the hypothesis that that the degree of narrow-band and broad-band slowing would be larger in the disconnected cortex with respect to the contralateral, and post-surgery with respect to the pre-surgery session. Further, we hypothesized that the disconnected cortex would show a greater increase following surgery, in the degree of narrow- and broad-band slowing, with respect to the contralateral cortex.
Greater narrow-band slowing is indexed by larger Slow Delta Power values, while greater broad-band slowing is indexed by more negative Spectral Exponent values (i.e. larger in absolute values). The results are described in the main text and reported in **Supplementary Table C.** Briefly, following surgery, larger narrow-band and broad-band slowing was observed in the disconnected cortex–as compared to the pre-surgery session, and as compared to the contralateral cortex–leading to a significant inter-hemispheric asymmetry, which was larger relative to the pre-surgery session.

**Slow Delta Power (0.5-2 Hz)**

| Term | F-stat | DF1 | DF2 | p |
| --- | --- | --- | --- | --- |
| Intercept | 504,70 | 1 | 36 | **9,02E-23** |
| SpatialSide | 0,04 | 1 | 36 | 0,83 |
| Time | 9,21 | 1 | 36 | **0,0044** |
| SpatialSide*Time | 15,74 | 1 | 36 | **0,0003** |

**Spectral Exponent (0.5-20 Hz)**

| Term | F-stat | DF1 | DF2 | p |
| --- | --- | --- | --- | --- |
| Intercept | 878,15 | 1 | 36 | **6,99E-27** |
| SpatialSide | 4,16 | 1 | 36 | **0,04889** |
| Time | 22,04 | 1 | 36 | **3,8E-05** |
| SpatialSide*Time | 46,43 | 1 | 36 | **5,77E-08** |

**Supplementary Table B. ANOVA of mixed effect models reveal a significant interaction between time and spatial side, indexing that the surgical disconnection differently affected each hemisphere.** Degrees of Freedom relative to the ANOVA Fisher statistic (F-stat) are reported as DF1 and DF2. Significant p-values of the F tests, for each term of the linear mixed effect model, are highlighted in bold.

|  | **Paired Contrast** | | **Average values** | |  | **Stat.** | **p-values** | |
| --- | --- | --- | --- | --- | --- | --- | --- | --- |
| **Slow Delta Power (0.5-2 Hz)** | | |  |  |  |  |  |  |
| **Context** | **Group 1** | **Group 2** | **μ Group1** | **μ Group2** | **d** | **t** | **p** | **pFDR** |
| Difference Post-Pre | Contra  lateral | Disconnected | 0,129 | 0,601 | -1.020 | -3,146 | **0,01181** | **0,01969** |
| Pre | Contra  lateral | Disconnected | 1,750 | 1,770 | -0.069 | -0,228 | 0,82464 | 0,82464 |
| Post | Contra  lateral | Disconnected | 1,879 | 2,372 | -1.208 | -4,245 | **0,00216** | **0,01079** |
| Contralateral | Pre | Post | 1,750 | 1,879 | -0.454 | -1,462 | 0,17765 | 0,22206 |
| Disconnected | Pre | Post | 1,770 | 2,372 | -1.446 | -3,215 | **0,01058** | **0,01969** |
|  |  |  |  |  |  |  |  |  |
| **Spectral Exponent (0.5-20 Hz)** | | |  |  |  |  |  |  |
| **Context** | **Group 1** | **Group 2** | **μ Group1** | **μ Group2** | **d** | **t** | **p** | **pFDR** |
| Difference Post-Pre | Contra  lateral | Disconnected | -0,175 | -0,644 | 1.501 | 6,464 | **0,00012** | **0,00020** |
| Pre | Contra  lateral | Disconnected | -1,446 | -1,590 | 0.734 | 2,395 | **0,04022** | 0,05027 |
| Post | Contra  lateral | Disconnected | -1,621 | -2,234 | 2.050 | 7,118 | **0,00006** | **0,00020** |
| Contralateral | Pre | Post | -1,446 | -1,621 | 0.618 | 1,786 | 0,10769 | 0,10769 |
| Disconnected | Pre | Post | -1,590 | -2,234 | 2.962 | 6,455 | **0,00012** | **0,00020** |

**Supplementary Table C: Pairwise Contrasts across spatial and temporal conditions on EEG slowing features.** The context column specifies how the values are considered: either within a temporal condition (Pre, Post), within a spatial condition (Contralateral, Disconnected), or as a difference between the two temporal conditions (Post-Pre difference). The Paired Contrasts columns specify the groups (spatial or temporal) considered for the contrast. The ‘Average values’ display the values within each of the two groups (μ Group1, μ Group2). The ‘Stat.’ column displays Cohen’s d (a common measure of the effect size), the t-statistic for each contrast, resulting from a paired t-test across the two groups. For each feature (Slow Delta Power, top; Spectral Exponent, bottom), the p-values columns display the uncorrected one-sided p-values (p) from the t-test and the p-values after multiple comparison correction with a false discovery rate approach across the columns (p-FDR), by means of the linear-step up procedure originally introduced by Benjamini and Hochberg (1995). Significant p values (p <0.05), either before or after correction for multiple comparisons are highlighted in bold. Note the overall larger effect sizes for the spectral exponent, as estimated by Cohen's d values.

**S5 - Comparing narrow-band vs broad-band slowing in cortical isolation**

We adopted a bootstrap procedure to estimate whether the neurophysiological correlates of hemispherotomy were better captured by a narrow-band or by a broad-band slowing.
We considered four contrasts of interest: two spatial contrasts (Contralateral vs Disconnected, in both Pre and Post -session) and two temporal contrasts (Pre vs Post, for both the Contralateral and Disconnected side). Specifically, for each iteration of 1000 bootstrap resamples with replacement, we assessed a specific contrast by means of a paired t-test and considered the strength of statistical evidence for each of the two features (T statistic), and the difference in statistical evidence between the two features (ΔT statistic).
Thus, we obtained a bootstrapped distribution of T for each feature (displayed in **Supplementary Figure D**), and a bootstrapped distribution of ΔT indexing the difference in statistical evidence between features.
 Similarly, we derived the T and ΔT statistic for the observed sample, considered entirely.

We then considered the proportion of bootstraps in which the bootstrapped ΔT statistic had an opposite sign to the observed ΔT statistic, P(sign(ΔTboot ) ≠ sign(ΔTobserved)), denoted for brevity as Psign≠ ; e.g. for an observed negative difference in T statistics between the two features (ΔT <0), we report the proportion of positive ΔT across bootstraps, P(ΔT_boot_>0). Note that, for this specific supplementary analysis, in order to compare the size of statistical evidence of the spectral exponent with that of the Slow Delta Power, we considered the absolute value of the (always negatively signed) Spectral Exponent, resulting in T-statistics with a coherent sign across features.

The Spectral Exponent, as compared to Slow Delta Power, showed stronger statistical evidence in detecting inter-hemispheric differences in the pre-surgical disconnection session (Spectral Exponent: T(9)=2.395; Slow Delta Power: T(9)=0.228, Difference in T-statistics between features: ΔT=2.167, P_sign≠_ = 0.032, top-left panel).
Further, the Spectral Exponent, as compared to Slow Delta Power, showed stronger statistical evidence in detecting the change due to hemispherotomy in the disconnected hemisphere (Spectral Exponent: T(9)=6.455; Slow Delta Power: T(9)=3.215, difference in T-statistics between features: ΔT=3.24, P_sign≠_ =0.042, bottom-right panel).

**
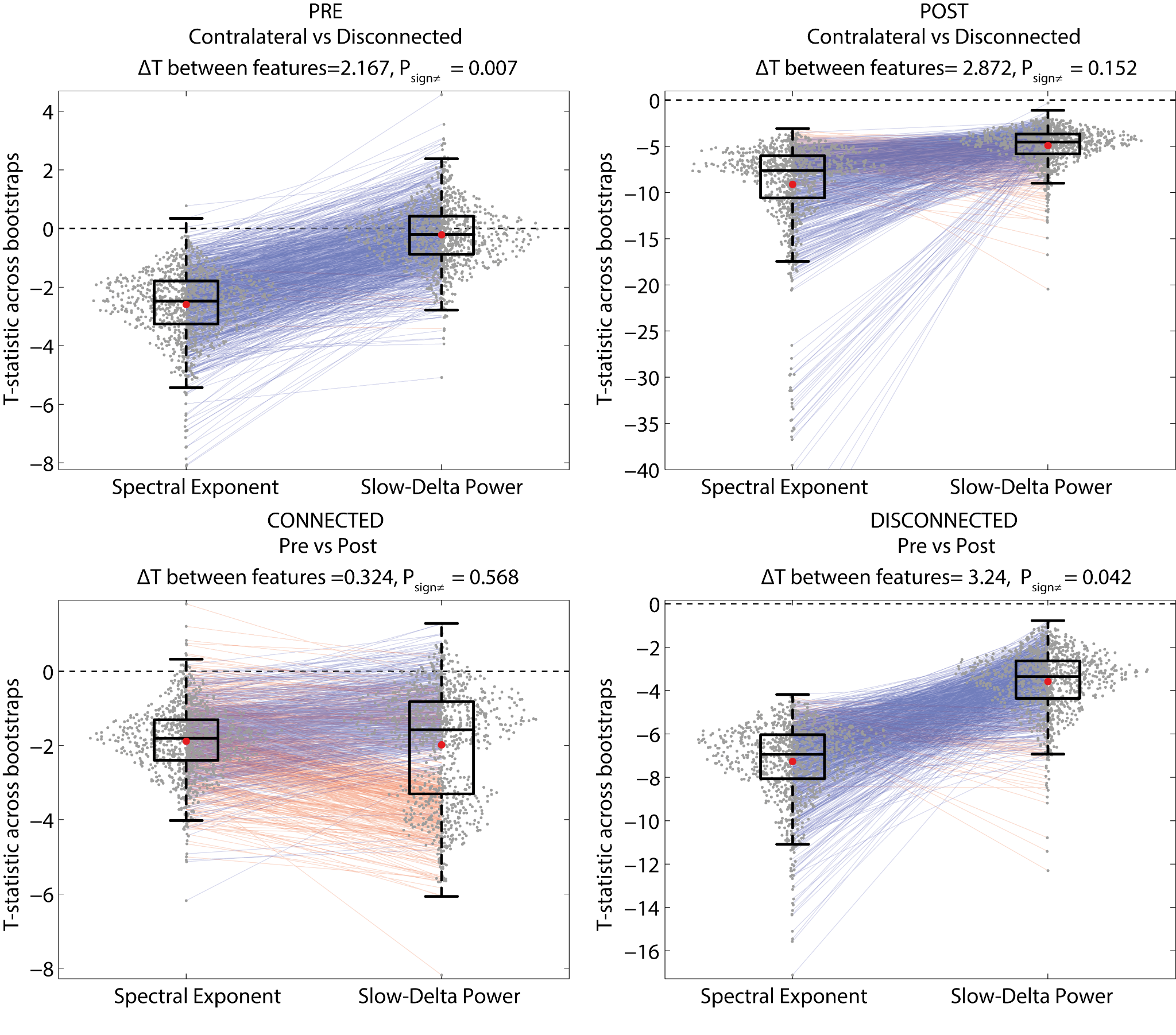
**

**Supplementary Figure F. Bootstrap analysis revealed that the Spectral Exponent showed stronger statistical effects than Slow Delta Power in detecting the electrophysiological effect of cortical isolation on the disconnected hemisphere**. The features’ distributions of T-statistics, drawn pairwise across bootstraps, revealed that the Spectral Exponent, as compared to Slow Delta Power, showed stronger statistical evidence in detecting inter-hemispheric differences in the pre-surgical disconnection session (**top-left** panel, PRE, contralateral vs disconnected contrast), as well as in detecting the change due to hemispherotomy in the disconnected hemisphere (**bottom-right** panel, Pre vs Post difference, DISCONNECTED). The mean of each distribution is shown as a red dot; bootstrap iterations whereby the difference in T-values between features (ΔT) showed an opposite sign are plotted in different colors (transparent blue and red lines); the title reports the observed ΔT between features relative to the whole sample, and the proportion of bootstraps in which the bootstrapped ΔT statistic had an opposite sign to the observed ΔT statistic (P_sign≠_).

**SF - Controlling for Etiology and Type of Hemispherotomy**

We report below the ratio of patients in several subgroups that followed the pattern described in the main text, namely, the finding of an inter-hemispheric spatial asymmetry after surgery and a pre-post temporal change within the pathological cortex (see main results). Moreover, we display in **Supplementary Figure G** the EEG features (Slow Delta power and spectral exponent) of the patient dataset (in the disconnected and contralateral cortex), along with their pre-post change (as in main Figure 2), while also revealing at once the type of etiology (cortical malformation, perinatal ischemic stroke) and the type of hemispherotomy (whole-hemisphere, temporo-parieto-occipital) for each patient.

**SF.1 - Etiology**

Considering the amount of Slow Delta power (0.5-2 Hz), 4/4 patients with a cortical malformation displayed spatial asymmetry after surgery, and 4/4 patients showed a pre-post increase. Further, 5/6 patients with pre or perinatal ischemic stroke displayed spatial asymmetry after surgery and a 5/6 showed a pre-post increase.

Considering the spectral exponent (0.5-20 Hz), 4/4 patients with a cortical malformation displayed spatial asymmetry after surgery and 4/4 patients showed a pre-post decrease. Further, 6/6 patients with pre or perinatal ischemic stroke displayed spatial asymmetry after surgery and a 6/6 showed a pre-post increase.

**SF.2 - Type of hemispherotomy**

Considering the amount of Slow Delta power (0.5-2 Hz), 3/3 patients with temporo-parieto-occipital disconnection displayed spatial asymmetry after surgery and a 3/3 showed a pre-post increase. Further, 6/7 patients with hemispherotomy of a whole-hemisphere displayed spatial asymmetry after surgery, and 6/7 patients showed a pre-post increase.

Considering the spectral exponent (0.5-20 Hz), 3/3 patients with a temporo-parieto-occipital disconnection displayed spatial asymmetry after surgery and 3/3 patients showed a pre-post decrease. Further, 7/7 patients with hemispherotomy of a whole-hemisphere displayed spatial asymmetry after surgery and a 7/7 showed a pre-post increase.


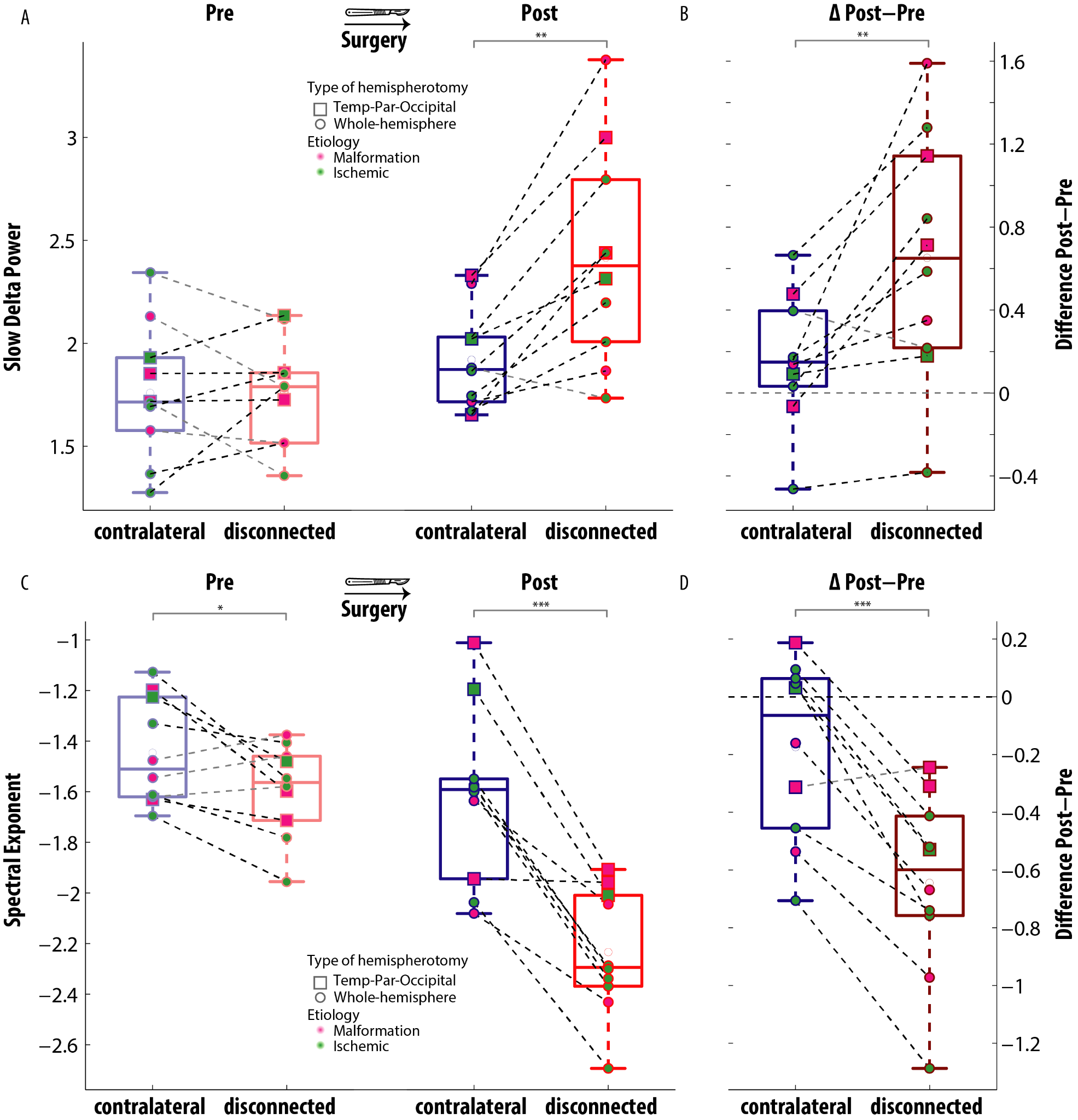


**Supplementary Figure G. Narrow- and broad-band spectral changes after surgery reveal a marked EEG slowing of the isolated cortex, robust across etiology and type of hemispherotomy.** This figure mirrors **Figure 2** from the main text, graphically highlighting the different patients in terms of etiology and type of disconnection. Specifically, patients who underwent complete hemispherotomy are marked with a circle, while those who underwent temporo-parieto-occipital disconnection are marked with a square. Additionally, patients with a cortical developmental malformation as the underlying etiology are shown in red, whereas those with pre- or perinatal stroke sequelae are shown in green. **A)** Slow Delta Power (0.5-2 Hz) was significantly larger in the disconnected than in the contralateral cortex only in the session after surgery (**Figure 2A**). This pattern was observed in 4/4 patients with a cortical malformation, 5/6 patients with pre/perinatal ischemic stroke, 3/3 patients with temporo-parieto-occipital disconnection, 6/7 patients with hemispherotomy **B)** Slow Delta Power increased significantly pre to post surgery only in the disconnected cortex (**Figure 2B**); this pattern was observed in the same subset of patients described above, across the subgroups of etiology and type of hemispherotomy. **C)** The spectral exponent (0.5-20 Hz) was significantly more negative in the disconnected than in the contralateral cortex in the session after surgery (**Figure 2C**). This pattern was observed in all conditions: in 4/4 patients with a cortical malformation, 6/6 patients with pre/perinatal ischemic stroke, 3/3 patients with temporo-parieto-occipital disconnection and 7/7 patients with hemispherotomy. **D)** The PSD became significantly steeper from pre to post surgery only in the disconnected cortex, as indexed by the spectral exponent (**Figure 2D**); this pattern was observed in all the patients, across the subgroups of etiology and type of hemispherotomy. The data underlying this figure are provided in the file FigS7_Data, publicly available in the repository at the following <https://doi.org/10.5281/zenodo.16936516>

**SF.3 - Comparing EEG slowing after cortical disconnection between patients with whole Hemispherotomy and those with Temporo-Parieto-Occipital disconnection**

In our study, we included patients with a lateralized surgical disconnection of different extents, from a large cortical portion (temporo-parieto-occipital disconnection, TPO, 3 patients), to a whole hemisphere (hemispherotomy, 7 patients). Thus, we here ascertained whether the two sub-groups showed a similar narrow-band and broad-band EEG slowing induced by the surgical disconnection; while in the main text we focused on the overall pattern observed across both sub-groups. As reported in the Methods section of the main text, for each individual patient we considered only the EEG channels placed over the scalp above the disconnected cortex post-surgery (6 nearest neighbor bipolar derivations for TPO, and 15 for hemispherotomy), and over the homologous contralateral region, then considered the median value across electrodes of each EEG feature (Slow Delta power and spectral exponent). A close inspection of the boxplots in Figure 3 reveals that patients who received a TPO (displayed as square markers) display the same qualitative pattern reported in the main text across all patients (hemispherotomy patients are displayed as circles). All three TPO patients showed an inter-hemispheric asymmetry, and a change in the disconnected cortex following surgery, that was coherent with what was observed in the whole sample and in hemispherotomy patients, for both the narrow-band and broad-band slowing EEG features.

Specifically, TPO patients showed larger slowing (i.e. larger delta power and more negative spectral exponent) in the disconnected cortex following surgery as compared to the contralateral (3/3 patients), and as compared to the same cortex before surgery (3/3 patients). Similarly, hemispherotomy patients showed a larger degree of slowing in the disconnected cortex following surgery as compared to the contralateral (6/7 showed larger Slow Delta power and 7/7 more negative spectral exponent), and also as compared to the same cortex before surgery (6/7 showed increased Slow Delta power and 7/7 decreased spectral exponent to more negative values).

Further, pairwise t-tests were performed in the sub-group of hemispherotomy patients only (7 patients, leading to contrasts with sufficient statistical power). In this subgroup, we could replicate the effects of surgery reported in the main text across the whole sample. Hemispherotomy patients showed a larger degree of slowing in the disconnected hemisphere following surgery as compared to the contralateral (larger Slow Delta power: t(6)= -2.933, p=0.013; more negative negative spectral exponent: t(6)=9.452, p<.00001), and also as compared to the same hemisphere before surgery (increased Slow Delta power: t(6)= 2.365, p = 0.028; decreased spectral exponent to more negative values: t(6)=6.956, p=0.0002). Moreover, the disconnected hemisphere underwent a greater change following surgery as compared to the contralateral hemisphere, leading to increased inter-hemispheric asymmetry following surgery (Slow Delta power: t(6)= -2.431, p = 0.026; decreased spectral exponent to more negative values: t(6)=8.832, p =0.0001).
In sum, we ascertained that cortical disconnection resulted in a degree of neurophysiological slowing (both narrow-band and broad-band) localized in the disconnected cortex that was similar between patients with TPO disconnection and those with hemispherotomy of a whole hemisphere. Studies with a larger sample size of patients with a TPO disconnection are needed to better observe whether these patients present subtle neurophysiological specificities.

**S6.4 - Mixed effect models including ‘type of hemispherotomy’ as a covariate**

To account for the potential contribution of lesion extension, we re-estimated the mixed-effects models for both spectral exponent and Slow Delta power including type of hemispherotomy (whole hemisphere vs temporo-parieto-occipital disconnection) as a covariate, and assessed fixed effects using marginal ANOVA.

For Slow Delta power, the inclusion of the type of hemispherotomy did not alter the pattern of effects observed previously. The updated model revealed no significant main effect of spatial side (F(1, 32) = 0.25, p = 0.617), nor of the type of hemispherotomy (F(1, 32) = 1.74, p = 0.197), but confirmed a significant main effect of time (F(1, 32) = 8.11, p = 0.0076). Critically, the interaction between spatial side and time remained significant and robust (F(1, 32) = 13.80, p = 0.0008), consistent with the previous model. No significant interactions were observed involving type of hemispherotomy (all p > 0.45), indicating that lesion extension did not modulate the spatial or temporal dynamics of Slow Delta activity.

Similarly, for the spectral exponent, the inclusion of the type of hemispherotomy did not alter the pattern of effects observed previously. As in the original model, we found a significant main effect of spatial side (F(1, 32) = 5.41, p = 0.026), a strong effect of time (F(1, 32) = 18.03, p < 0.001), and a large, highly significant interaction between side and time (F(1, 32) = 51.89, p < 0.000001). The type of hemispherotomy itself had no significant main effect (F(1, 32) = 0.39, p = 0.538), and its interactions with side and time did not reach significance (all p > 0.07), although the interaction with time showed a statistical trend (F(1, 32) = 3.44, p = 0.073).

Altogether, these findings indicate that using type of hemispherotomy as a covariate in a mixed effect model does not alter the core spatiotemporal effects observed for each of the two EEG spectral features; hence lesion size is not a major contributor to the interindividual variance in this context. Nonetheless, future studies incorporating larger sample sizes and a broader range of disconnection types with different lesion volumes and locations may uncover subtle modulatory effects on the electrophysiological slowing induced by hemispherotomy.

**S7 - Comparison of spectral exponent with the reference sample distribution of wake and NREM sleep**

In order to compare the spectral exponent values of the patients with cortical disconnection to those of the reference pediatric sample, we estimated the typical difference between pairs of conditions across the datasets, through bootstrap analysis. Specifically, bootstrap analysis estimated the distribution of pairwise differences between the mean spectral exponent values in healthy pediatric participants (during wakefulness or N1, N2 or N3 sleep) on the one hand, and the mean values in the isolated or contralateral cortex (before or after surgery) on the other hand. Results are shown in **Supplementary Figure H.**

Accordingly, the disconnected cortex after surgery had mean spectral exponent values resembling those of deep sleep (similarly close to N2 and N3 sleep), but considerably distinct from those of N1 sleep and, even more, from those of wakefulness, as shown by the large positive differences. The same cortex before surgery showed mean values closest to N1 sleep. The contralateral cortex, both before and after surgery, showed mean values that were close to those of wakefulness, albeit on the lower percentiles, and considerably distinct from those of N2 and N3 sleep, as shown by the large negative differences.


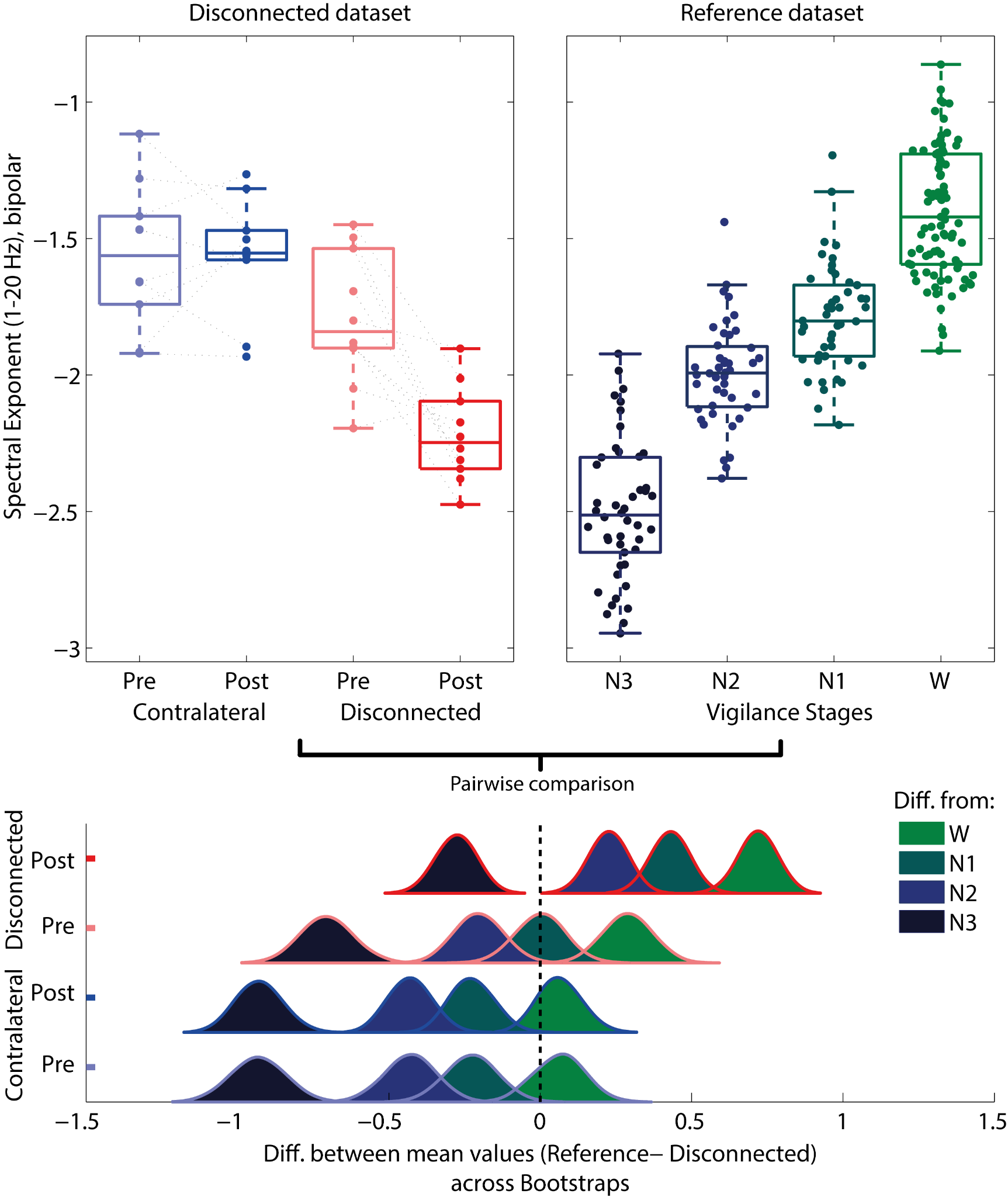


**Supplementary Figure H. Comparing the Spectral Exponent in hemispherotomy and in a reference pediatric sample across Wake and Sleep revealed that the values of the isolated hemisphere are compatible with those of deep NREM sleep, between N2 and N3.** The figure extends **Figure 3** of the main text. The top panel displays spectral exponent (1-20 Hz) values in the two datasets: in patients before and after cortical isolation (the disconnected dataset, top-left panel), and in a reference pediatric sample across wake and NREM sleep (reference sleep dataset, top-right panel). Bootstrap analysis estimated the distribution of pairwise differences between the mean of conditions, across the two datasets. A mean difference of zero corresponds to the central dotted line, with positive differences on the right. The distribution that is closest to the zero- mean difference line corresponds to the pair of states that show the smallest mean difference.

**SH - Correlation analysis between age and EEG features among hemispherotomy patients.**


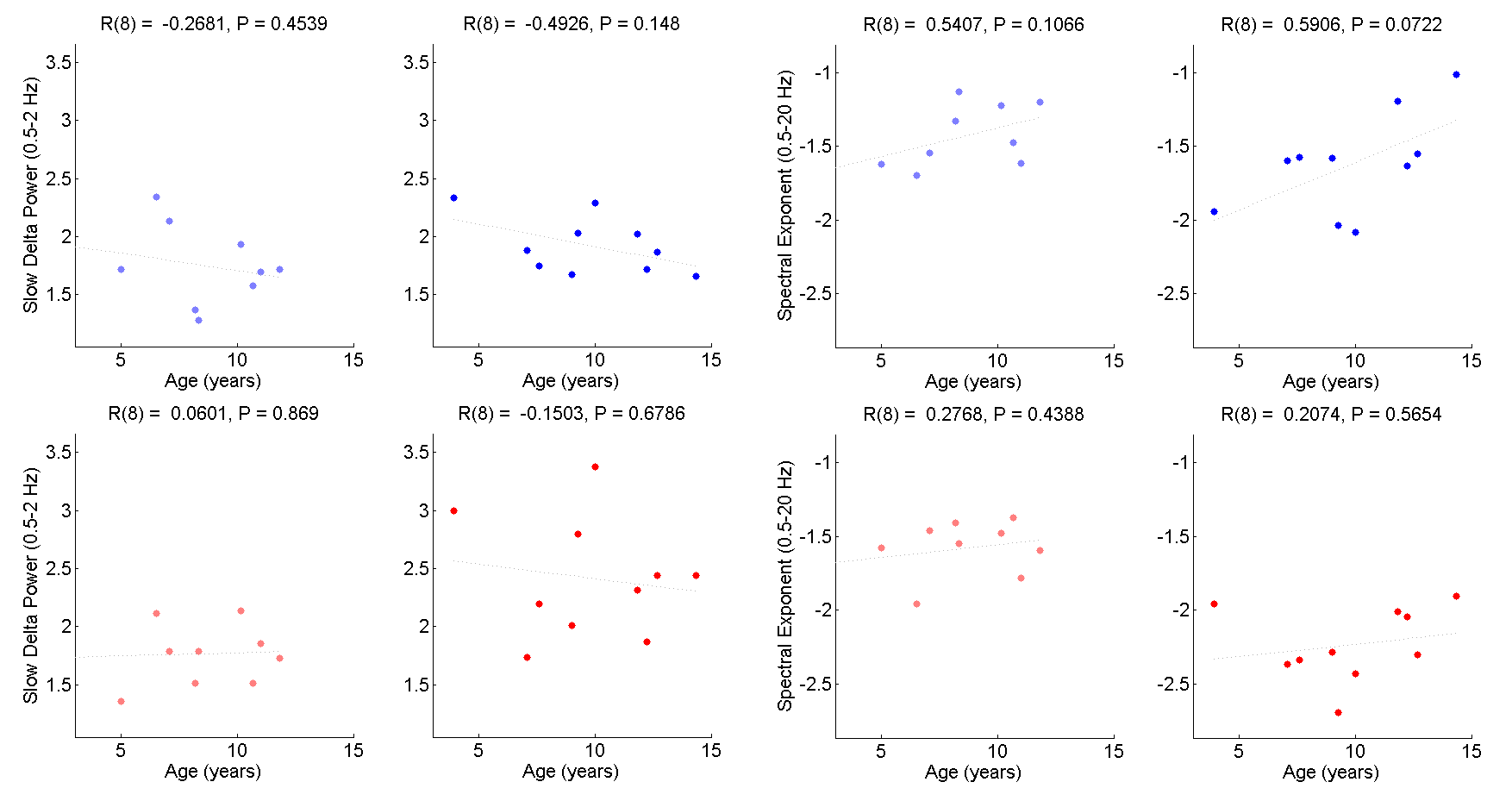


**Supplementary Figure I. Correlation analysis among hemispherotomy patients revealed the absence of significant correlations between age and EEG features, either Slow Delta power or spectral exponent.** The left panel of the figure shows that both before (top row) and after (bottom row) disconnection, in both the disconnected hemisphere (right) and the contralateral one (left), there is no correlation between age and spectral exponent. The right panel similarly shows that neither before nor after disconnection, in either hemisphere, is there a correlation between age and the Slow Delta band envelope. Beyond marginal trends observed for the spectral exponent in the contralateral cortex, no significant correlations with age were found in the disconnected hemisphere, likely due to the high inter-individual variability typical of severe epilepsy—particularly evident in the pathological cortex. Please see **Favaro et al., 2023** for a detailed analysis of the relation between maturation and EEG aperiodic activity in neurotypical pediatric children.

**SI - Supporting age-matched comparison of spectral exponent between Patient and Reference datasets**

Given the well-known impact of age on EEG features (Kurth et al., 2010; Gorgoni et al., 2020; Favaro et al., 2023), we conducted supplementary analyses to factor out the effect of age in our findings related to the comparison between the patient and the pediatric reference dataset. Primarily, we sought to verify if the electrophysiological profile of the disconnected cortex resembled deep NREM sleep (main **Fig. 3**), beyond any potential effects of age. Accordingly, we applied a pairwise age-matching approach (illustrated in **Supplementary Figure J**) to compare the spectral exponent of the patient dataset across conditions (pre-post, disconnected-contralateral) to those of the pediatric reference dataset across wakefulness and NREM sleep stages (**Supplementary Figure K**). Moreover we applied the same age-matched approach to compare slow wave properties of the disconnected cortex of the patient dataset to those of the pediatric reference dataset in wakefulness and deep NREM sleep (N2 - N3) (**Supplementary Figure O**).


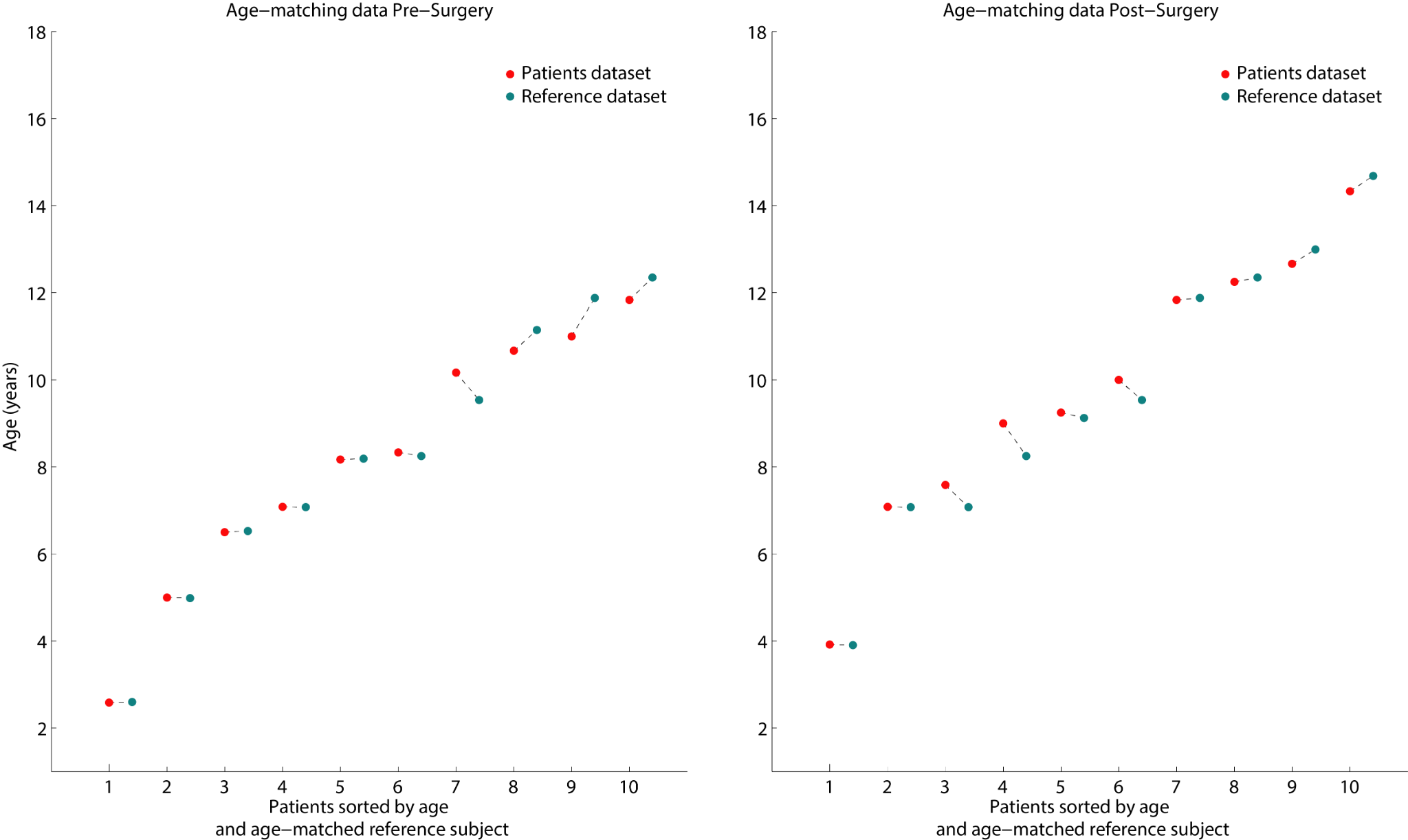


**Supplementary Figure J. Matching subjects by age across the Patient and the Reference dataset.** The figure shows age-matching across the patient dataset and a subset of the reference dataset; with each subplot comparing the age of patients to that of matched control subjects from the pediatric reference dataset, separately for pre- and post-surgery. The x-axis represents subject number (sorted by age), and the y-axis shows age in years. In both panels (Pre-Surgery and Post-Surgery), each red dot (patient) is paired with a nearby blue dot (reference subject), indicating that age-matching was performed on an individual basis. The dashed lines connecting each pair indicate pairwise matching, and the vertical distance shows the age difference between each pair. All patients were age-matched within approximately 1 year or less, which supports a robust age-controlled comparison. Such tight age matching was intended to ensure that any differences observed between patients and the whole reference data (e.g., in spectral slope or slow-wave activity) would not be driven by age-related changes. Once age-matched subjects were identified, we compared the spectral exponent values between the patients dataset (in the disconnected or contralateral cortex, before or after surgery) and the the subsample of the pediatric reference dataset (either during wakefulness, N1, N2, or N3 sleep) (**Supplementary Figure K**), by computing the distribution of pairwise differences between age-matched subjects. We then identified the vigilance states showing the greatest similarity through a bootstrap analysis of the mean pairwise differences across conditions (violin plots in **Supplementary Figure K, panels B,D,F,H therein**).

We first qualitatively assessed how many hemispherotomy cases showed a steeper spectral decay than their age-matched counterparts during deep NREM sleep in the neurotypical reference dataset. Thus, we considered the proportion of age-matched cases in the patient dataset that showed a negative difference relative to N2 and N3 sleep of the pediatric reference dataset, indexing a steeper spectral decay in patients. The contralateral cortex did not exhibit a steeper spectral decay relative to either N2 or N3 sleep in any case (**Supplementary Figure K, panels F,H**). Before surgery, the pathological cortex showed a steeper spectral decay—indexed by negative differences—relative to N2 sleep in 2/10 cases and to N3 sleep in 0/10 cases (**Supplementary Figure K, panel B**). After surgery, the disconnected cortex showed a steeper spectral decay relative to N2 sleep in 9/10 cases and to N3 sleep in 3/10 cases (**Supplementary Figure K, panel D**). Conversely, the disconnected cortex showed a steeper spectral decay in all 10 cases compared to their age-matched counterparts during wakefulness or N1 sleep.

For the contralateral cortex, before and after surgery alike, the distribution of pairwise differences was centered between wakefulness and N1 sleep values; the bootstrap distribution of mean pairwise differences indicated larger similarity with wakefulness and N1 sleep (**Supplementary Figure K, panel F and H**), revealing a mild slowing (as qualitatively noted in previous literature by Smith et al., 1991) but still distinct from deeper NREM stages.

For the pathological cortex before surgery, the distribution of pairwise differences was centered tightly around zero relative to N1 sleep values; the bootstrap distribution of mean pairwise differences indicated the greatest similarity to light (N1) sleep (**Supplementary Figure K, panel B**).

For the disconnected cortex after surgery, the distribution of pairwise differences was centered between N2 and N3 sleep values; the bootstrap distribution of mean pairwise differences indicated larger similarity with deep (N2-N3) sleep (**Supplementary Figure K, panel D**).

**
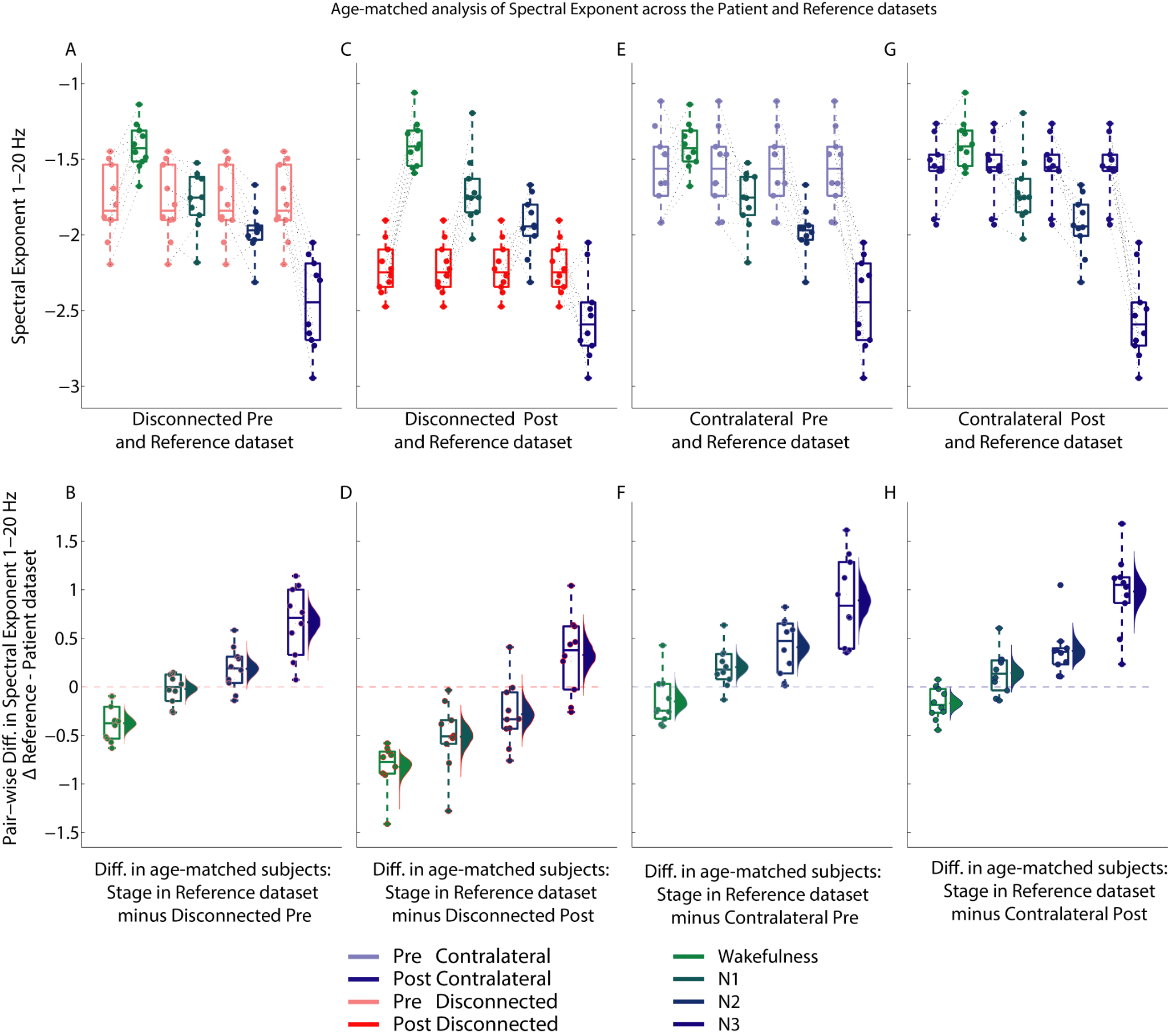
**

**Supplementary Figure K. Age-matched analysis comparing spectral exponent values in hemispherotomy to the pediatric reference dataset across wakefulness and NREM vigilance stages.** Each column presents two plots corresponding to a condition of the Patient dataset (Left to Right: Disconnected cortex pre- and post-surgery, followed by Contralateral cortex pre- and post-surgery), compared to each vigilance stage of the pediatric Reference dataset (wakefulness, N1, N2, N3, colored in shades spanning from green to blue). The first row (**A,C,E,G**) shows the absolute spectral exponent values, with lines connecting age-matched subjects across the Patient and Reference datasets. The second row (**B,D,F,H**) displays pairwise differences in spectral exponent: each dot represents the difference for a subject pair, calculated by subtracting the value of a subject in a given vigilance stage of the Reference dataset from that of the corresponding subject in the Patient dataset. Violin plots depict the bootstrap distribution of mean pairwise differences across subjects, thereby providing an estimate of the expected mean distance between paired conditions.
Overall, age-matched results revealed a mild slowing in the contralateral cortex, both pre-surgery (**E,F**) and post-surgery (**G,H**), with spectral exponent values intermediate between Wakefulness and N1. Importantly, age-matched results replicated the main findings (**Fig. 3**): spectral exponent values in the Disconnected cortex pre-surgery closely aligned with those observed during N1 sleep (**A,B**), while post-surgery values were intermediate between N2 and N3 sleep (**C,D**). The data underlying this figure are provided in the file FigS11_Data, publicly available in the repository at the following <https://doi.org/10.5281/zenodo.16936516>

**SJ - Slow Delta Amplitude Envelope**

We further estimated the amplitude envelope in the Slow Delta frequency band (0.5-2 Hz), obtained a density distribution across amplitude values, considered the median value across channels, and displayed the overall grand-average distribution across subjects.

As described in the main text, we first low-pass filtered the EEG signal (IIR forward and reverse Butter filter, 5th order) with a cutoff at 2 Hz, in alignment with the spectral domain analysis (Figure 1-2), then considered the absolute value of the Hilbert transform.

This procedure is illustrated graphically in **Supplementary Figure L.**


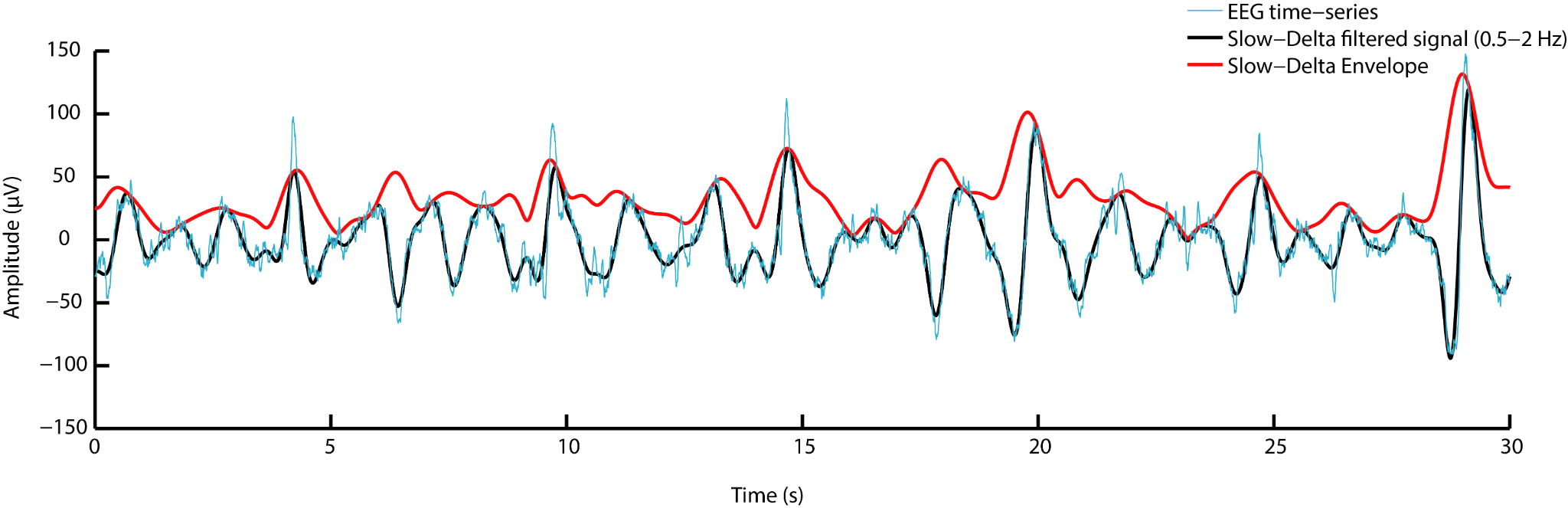


**Supplementary Figure L.** The EEG signal (cyan trace) is first band-pass filtered in the Slow Delta band (0.5–2 Hz; black trace). The envelope of the oscillation is then computed as the absolute value of the Hilbert transform of the filtered signal (red trace). This represents the instantaneous amplitude, calculated as the vector length in the complex plane, combining the real and imaginary components of the oscillatory dynamics (Cohen MX, 2014). Finally, the average value over time is retained, for subsequent statistical analyses.

A density plot of the typical span of the amplitude values across subjects is shown for both the patient and reference dataset (**Supplementary Figure M**).


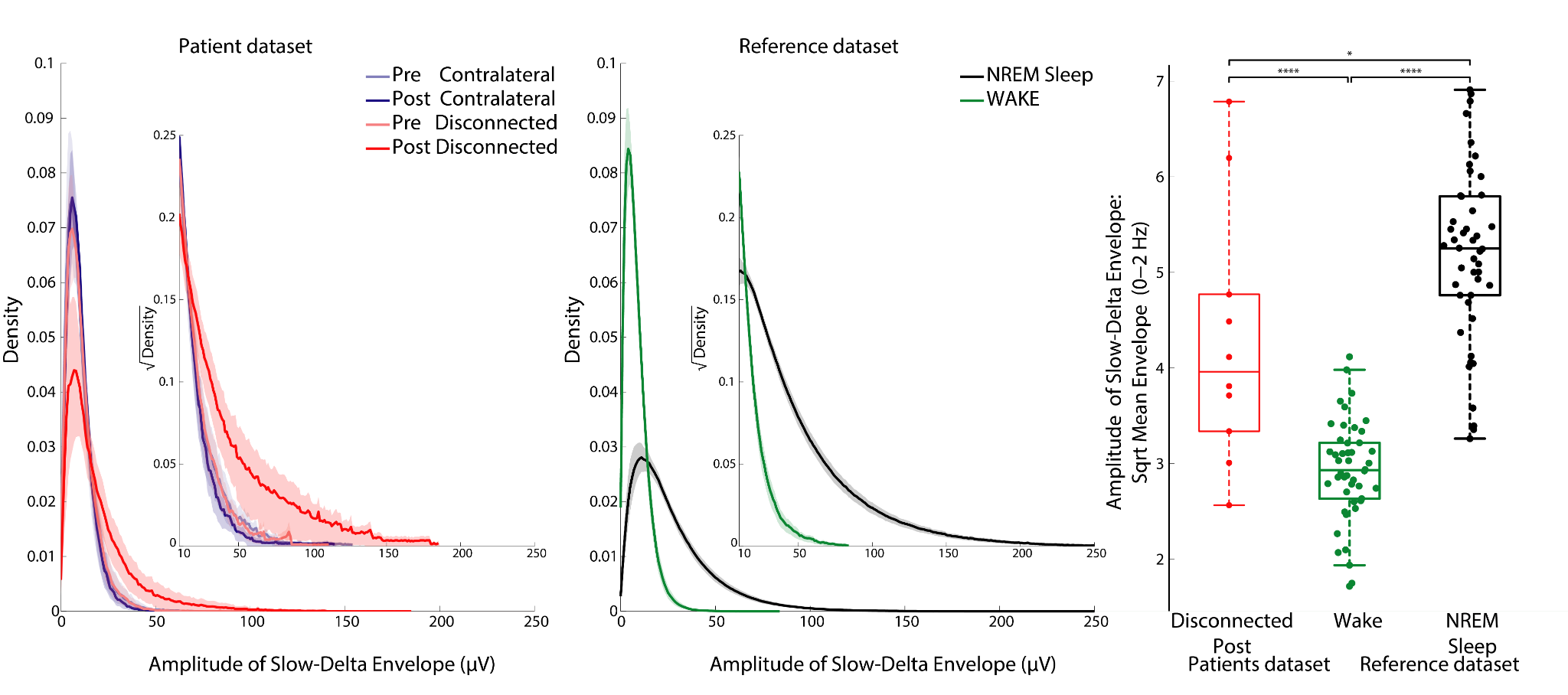


**Supplementary Figure M**. **Following surgery, the disconnected cortex displays a higher amplitude envelope of Slow Delta oscillations with respect to those observed in physiological wakefulness, yet smaller than that observed during physiological NREM sleep.** The first two panels display the typical density of observations for each value of the amplitude of the EEG activity, filtered in the Slow Delta band (0.5-2 Hz), in each condition. The median density over channels is computed; then, the grand-average across subjects is shown in bold, along with its confidence interval in a shaded area, for the patient dataset (**Left panel**), and for the reference pediatric dataset (**Middle panel**). The inset displays the square root of the density of amplitude values larger than 10 μV (i.e. approximately the modal value of all conditions), characterized by fat-tailed distributions; thus highlighting how often EEG filtered in the Slow Delta band displayed large amplitude excursions. The density plot reveals a high prevalence of large Slow Delta oscillations (high amplitude envelope of EEG filtered in the Slow Delta band) in the disconnected cortex, well above those observed in physiological wakefulness, yet smaller than those of physiological sleep. **Right panel**) The disconnected cortex displays large mean amplitude values of Slow Delta oscillations with respect to physiological wakefulness, but smaller than those of physiological sleep. Each dot corresponds to the mean amplitude, median across channels, of one subject (as in Figure 4, left panel).

**SJ.1 - Slow Delta PSD**

To corroborate the analysis on the amplitude Envelope of Slow Delta activity (0.5-2 Hz, see main **Results**), we performed a spectral analysis, comparing the strength of the Slow Delta oscillations of the disconnected cortex with those of sleep and wakefulness. Accordingly, we considered the mean PSD in the Slow Delta band (0.5-2 Hz), subsequently transformed by log10, and retained the median value across channels. The Slow Delta power of the disconnected cortex was larger than that of wakefulness (T(54)=−5.0034, P=6.3329e−06), yet smaller than that of sleep (T(54)=2.7612, P=0.0078515). Similar findings were obtained considering the amplitude envelope of Slow Delta oscillations **(**see main **Results)**.

**SK - Slow Delta Period**

**
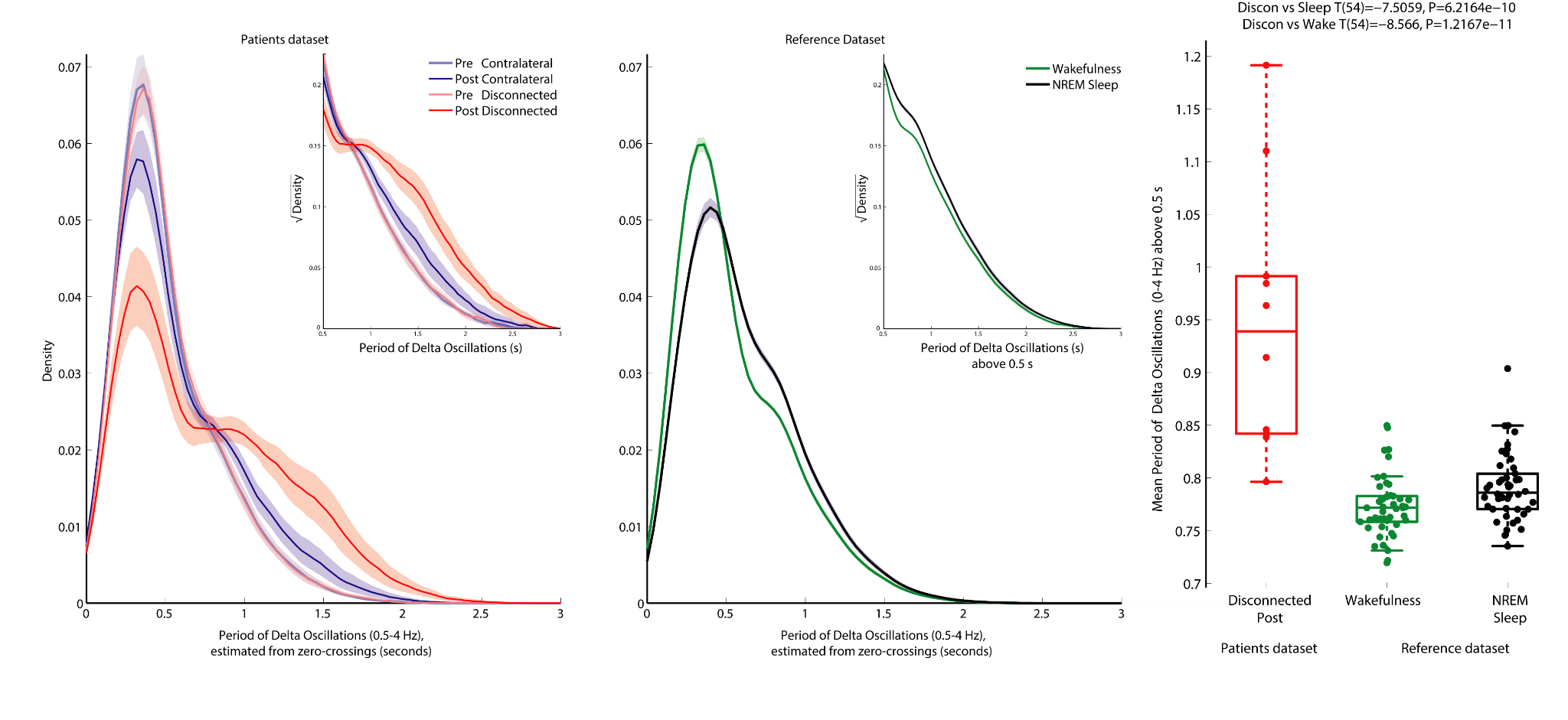
**

**Supplementary Figure N. Following surgery, the disconnected cortex displays a longer period of Slow Delta oscillations with respect to those observed in both physiological wakefulness and NREM sleep.** The first two panels display the typical density of observations for each value of the period of EEG activity filtered in the delta band (0.5-4 Hz), in each condition. The median density over channels is computed; then, the grand-average across subjects is shown in bold, along with its confidence interval in a shaded area, for the patient dataset (**Left panel**), and for the reference pediatric dataset (**Middle panel**). The inset displays the square root of the density of period values larger than 0.5 seconds (corresponding to 1/ 2 Hz); thus highlighting how often EEG activity corresponded to Slow Delta oscillations. The density plot reveals a high prevalence of long-lasting periods in the disconnected cortex, well above those observed in both physiological wakefulness and sleep. **Right panel)** The disconnected cortex displays a major slowing of mean periods corresponding to Slow Delta oscillations, with respect to both physiological wakefulness and sleep. Each dot corresponds to the mean of the periods above 0.5s, median across channels, of one subject (as in **Figure 4, right panel)** .

To estimate the typical period of all Slow Delta oscillations, we filtered activity in the delta band and discarded faster delta waves (filtering below 4 Hz and discarding waves shorter than 0.5 seconds, corresponding to 1/2 Hz), similar to standard practice in slow wave detection. The mean period of Slow Delta oscillations of the disconnected cortex was notably larger than that of wakefulness (T(54)=−8.566, P=1.2167e−11) as well as than that of sleep (T(54)=−7.5059, P=6.2164e−10). Overall, the analysis of Slow Delta activity revealed that the disconnected cortex showed slow waves with lower amplitude and longer period, with respect to those of physiological sleep.

**SL - Supporting age-matched comparison of slow wave properties between Patient and Reference datasets**

To further validate the analysis on slow wave properties of the disconnected cortex, we adopted the same age-matching approach detailed above (**Supplementary Figure J**) and applied it to the analysis presented in **Figure 4** (Mean amplitude of the Slow Delta envelope and period of Slow Delta oscillations in the disconnected hemisphere, compared with respect to wakefulness and NREM sleep in the reference pediatric sample). This analysis corroborated the main results on slow wave properties of the disconnected cortex in relation to pediatric reference values, by controlling for the effects of age.

The amplitude of the Slow Delta envelope of the disconnected cortex showed intermediate values between those of wakefulness and NREM (N2 and N3 jointly considered, **Supplementary Figure O, panel A**, consistently with the main results in **Figure 4A**); through age-matching its values resulted significantly larger than those of wakefulness ( T(9)=3.741, p = 0.0046, with 9/10 subjects showing a consistent pairwise difference) and not significantly different from those of NREM sleep (T(9)=-1.633, p = 0.13, with 8/10 subjects showing a consistent pairwise difference, **Supplementary Figure O, panel B**).

Further, the period of Slow Delta oscillations of the disconnected cortex showed values higher than those of both wakefulness and NREM (**Supplementary Figure O, panel C**, consistently with the main results in **Figure 4B**), through age-matching its values resulted significantly larger than those of both wakefulness (T(9)=4.783, p = 0.001, with 10/10 showing a consistent pairwise difference) and NREM sleep (T(9)= 3.686, p = 0.005, with 10/10 showing a consistent pairwise difference, **Supplementary Figure O, panel D**).

This analysis replicated the core results concerning the properties of Slow Delta activity reported in **Figure 4**: the disconnected cortex showed amplitude values intermediate between wakefulness and NREM sleep–with larger differences observed relative to wakefulness than to sleep–and significantly longer oscillation period than either condition, independently of age. Together, these results strengthen the age-independent validity of our findings.

**
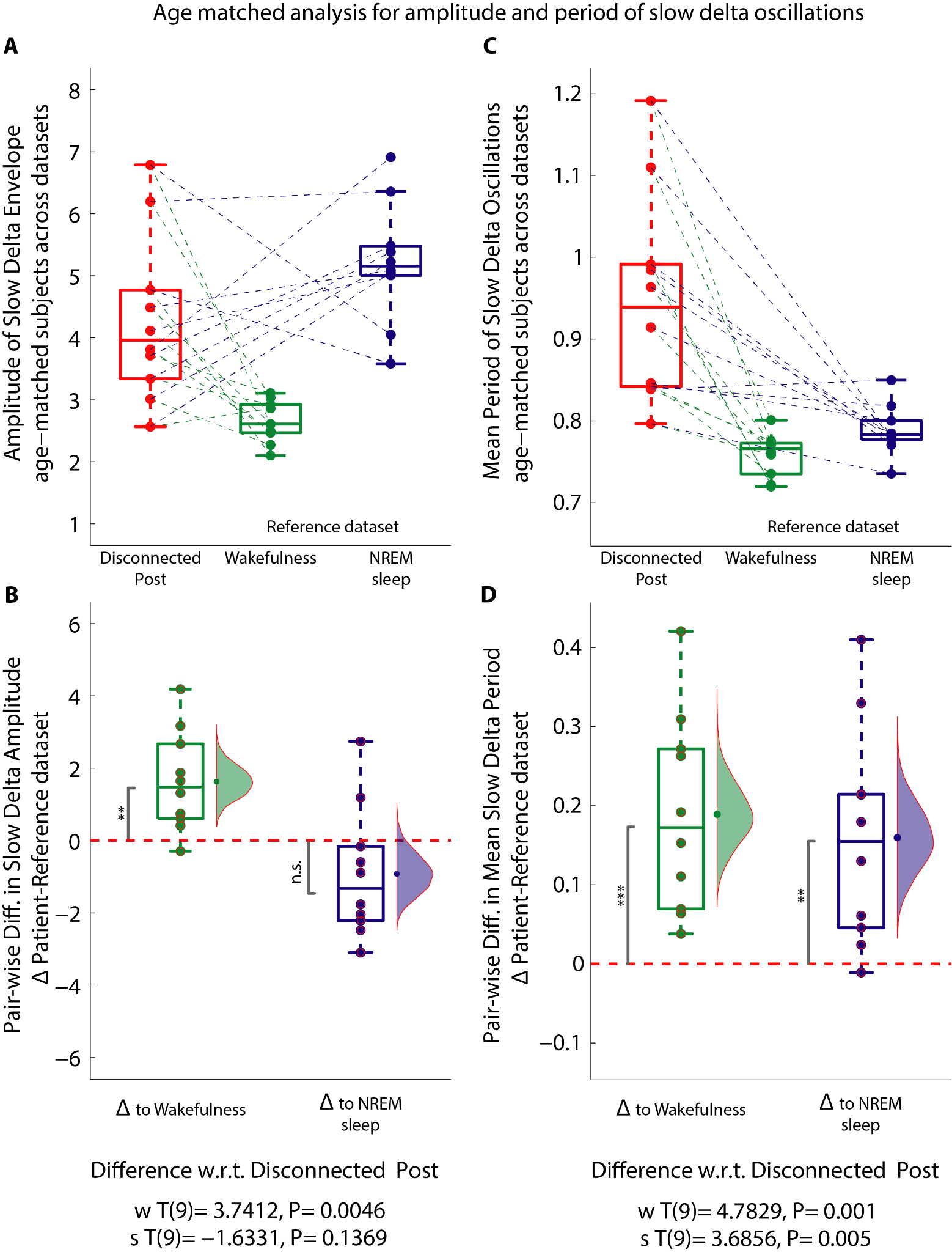
**

**Supplementary Figure O. Age-matched analysis of amplitude and period of Slow Delta oscillations in the disconnected cortex compared to the reference pediatric dataset during wakefulness and NREM sleep.** Results are drawn from the disconnected cortex in all 10 hemispherotomy patients age-matched to a subset of subjects from the reference dataset. (A–B) Slow Delta envelope amplitude comparison across datasets. (A) Boxplots show the amplitude of the Slow Delta envelope in the disconnected cortex compared to wakefulness and NREM sleep in the reference dataset, with lines connecting age-matched subjects across datasets.(B) Pairwise differences after age-matching, relative to wakefulness and NREM sleep. (C–D) Mean period of Slow Delta oscillations comparison across datasets. (C) Boxplots show the mean period in the disconnected cortex compared to the reference dataset, with lines connecting age-matched subjects across datasets. (D) Pairwise differences after age-matching. Overall, the disconnected cortex exhibited Slow Delta amplitude values intermediate between wakefulness and NREM sleep, and period values significantly longer than both wakefulness and NREM sleep, consistent with the findings reported in **Figure 4**. The data underlying this figure are provided in the file FigS15_Data, publicly available in the repository at the following <https://doi.org/10.5281/zenodo.16936516>

**SM - Regional aspects of EEG features among hemispherotomy patients.**

Given that the hotspot with maximal slow wave activity migrates from posterior to anterior regions in healthy children (Kurth et al., 2010) one may ask if the main findings can be observed even within two regions of interests, respectively considering a subset of electrodes in the anterior and posterior regions, in the subset of patients that received a hemispherotomy of a whole-hemisphere (N=7), excluding those with a hemispherotomy of the parieto-occipito-temporal area (N=3). In our low-density montage set-up with bipolar electrodes, we thus selected four bipolar derivations for the anterior region (Fp1-F3, F3-F7, F7-Fp1, F3-C3) and for the posterior regions (O1-T5, O1-P3, P3-T5, C3-P3), and considered their median value, as in the main analysis.

Concerning spatial inter-hemispheric contrasts (contralateral vs disconnected hemisphere) after surgery, Slow Delta Power was larger in the disconnected hemisphere than in the contralateral hemisphere, within the posterior region (T(6)=-3.201, p= 0.0186), and within the anterior region (T(6)=-3.267, p= 0.0171) (**Supplementary Figure P**)

Concerning temporal contrasts (Post-Pre surgery) of the disconnected hemisphere, Slow Delta Power increased post-surgery with respect to pre-surgery, within the posterior region (T(6)=2.526, p= 0.0449), but not within the anterior region, which only showed a marginal trend (T(6)=1.937, p= 0.1008). (**Supplementary Figure P**)


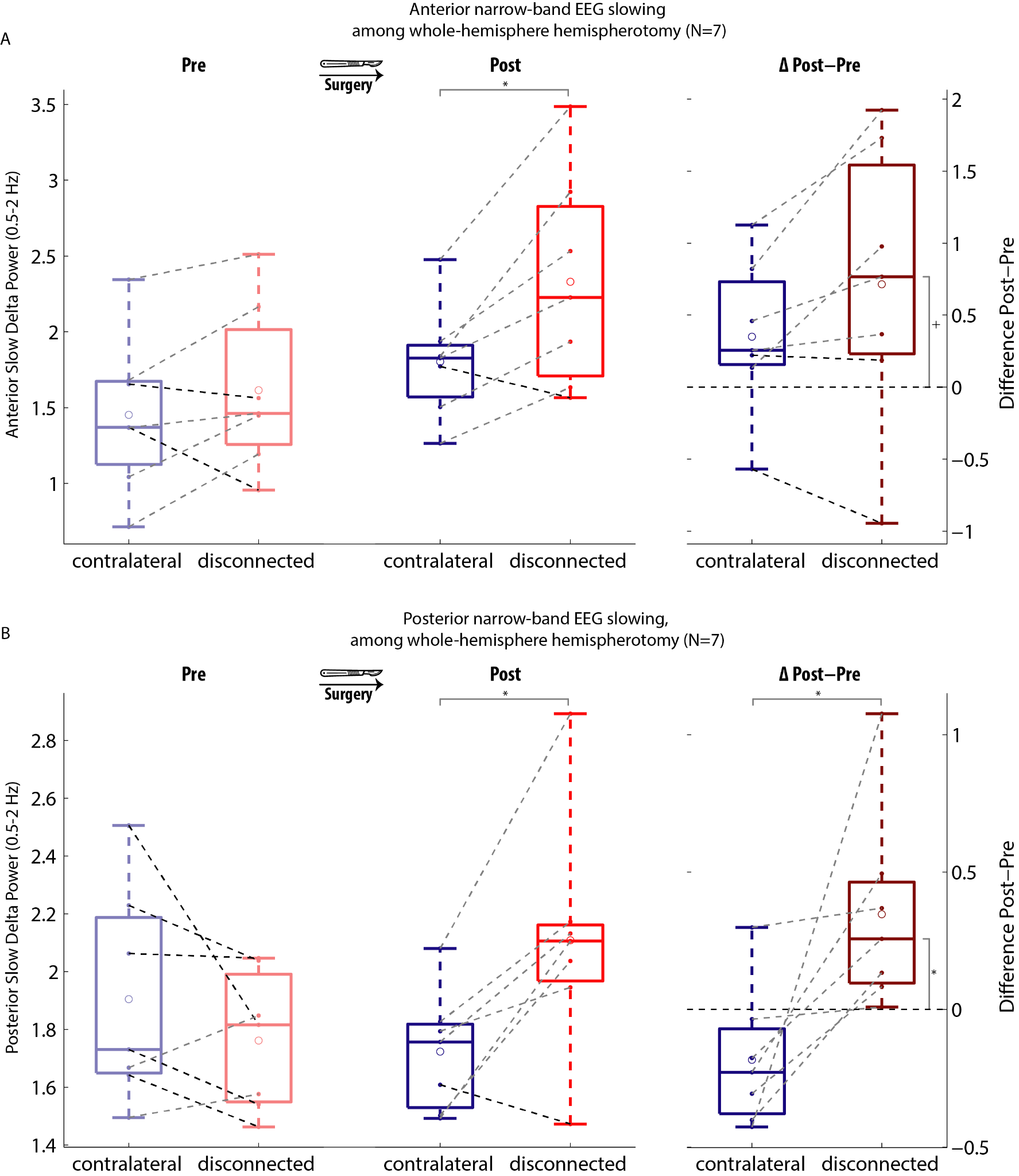


**Supplementary Figure P. Regional analysis of Slow Delta Power (0.5–2 Hz) following whole-hemisphere hemispherotomy revealed unihemispheric slowing after surgery, across anterior and posterior regions.** Boxplots show Slow Delta Power values of hemispherotomy patients for the contralateral and disconnected cortex, before (left-panel) and after surgery (middle panel), along with the temporal post-pre difference following surgery (right panel). Analyses were restricted to patients undergoing complete hemispherotomy (N=7) to enable separate analysis for the posterior and anterior regions. Each dot represents an individual patient; patients were connected by dashed lines across conditions. (A) Anterior region. After surgery, Slow Delta Power was larger in the disconnected hemisphere compared to the contralateral hemisphere (T(6) = –3.267, p = 0.0171); the temporal increase (Post vs. Pre) in the disconnected hemisphere revealed only a marginal trend (T(6) = 1.937, p = 0.1008). (B) Posterior region. After surgery, Slow Delta Power was significantly larger in the disconnected hemisphere compared to the contralateral hemisphere (T(6) = –3.201, p = 0.0186), and also increased significantly from pre- to post-surgery within the disconnected hemisphere (T(6) = 2.526, p = 0.0449). The data underlying this figure are provided in the file FigS16_Data, publicly available in the repository at the following <https://doi.org/10.5281/zenodo.16936516>

For consistency, we also performed the same regional analysis for the spectral exponent.

Concerning spatial inter-hemispheric contrasts (contralateral vs disconnected hemisphere) after surgery, the Spectral Exponent (0.5-20 Hz) was more negative in the disconnected hemisphere than in the contralateral hemisphere, within the posterior region (T(6)=9.172, p= 0.0001), and within the anterior region (T(6)=6.402, p= 0.0007). Concerning temporal contrasts (Post-Pre surgery) of the disconnected hemisphere, the Spectral Exponent (0.5-20 Hz) became more negative post-surgery with respect to pre-surgery, within the posterior region (T(6)=6.706, p= 0.0005), and within the anterior region (T(6)=4.61, p= 0.0037).

Overall, for Slow Delta power, both posterior and anterior regions showed a significant inter-hemispheric asymmetry after hemispherotomy of a whole-hemisphere; there was a pre-post change in the disconnected cortex that was significant only within the posterior region. Further, for the spectral exponent, posterior and anterior regions alike displayed both a significant inter-hemispheric asymmetry after hemispherotomy of a whole-hemisphere, and a significant pre-post change in the disconnected cortex. Thus, larger and more consistent effects across regions were observed with the spectral exponent than with Slow Delta power.

We reason that EEG measures derived from the amount of absolute power (such as Slow Delta power) are more affected by non-specific inter-individual variability than measures based on the relative distribution of power (such as the spectral exponent). This variability likely reflects differences in signal amplitude due to pathological heterogeneity (e.g. extent and localization of the epileptogenic zone, cortical atrophy) and surgical factors (e.g. individualized surgical approaches), which influence the amount of preserved gray matter and local conductivity. In contrast, relative measures—by capturing differences in spectral shape—retain inter-individual variability more closely associated with neurophysiological dynamics, while being less sensitive to confounds related to gray matter size and conductivity.

**SM.1 - Postero-Anterior specificities of Slow Delta Amplitude**

We explored potential regional specificities in the slow wave properties within the disconnected cortex, while accounting for age-specific effects. Thus, we performed an analysis of slow wave amplitude and period, separately in the anterior and posterior regions, in comparison to the reference dataset (**Supplementary Figure Q, top panels: A, C, E, G**), and applied an age-matching approach to control for the effect of age in each region (**Supplementary Figure Q, bottom panels: B,D,F,H**). In order to consider such regional differences, we restricted the analysis to hemispherotomy of a whole-hemisphere (N=7), thereby excluding those with a TPO hemispherotomy of (N=3), and considered median values over electrodes placed in an anterior region (Fp1-F3, F3-F7, F7-Fp1, F3-C3) and in a posterior region (O1-T5, O1-P3, P3-T5, C3-P3), for both the patient and the reference dataset.

*
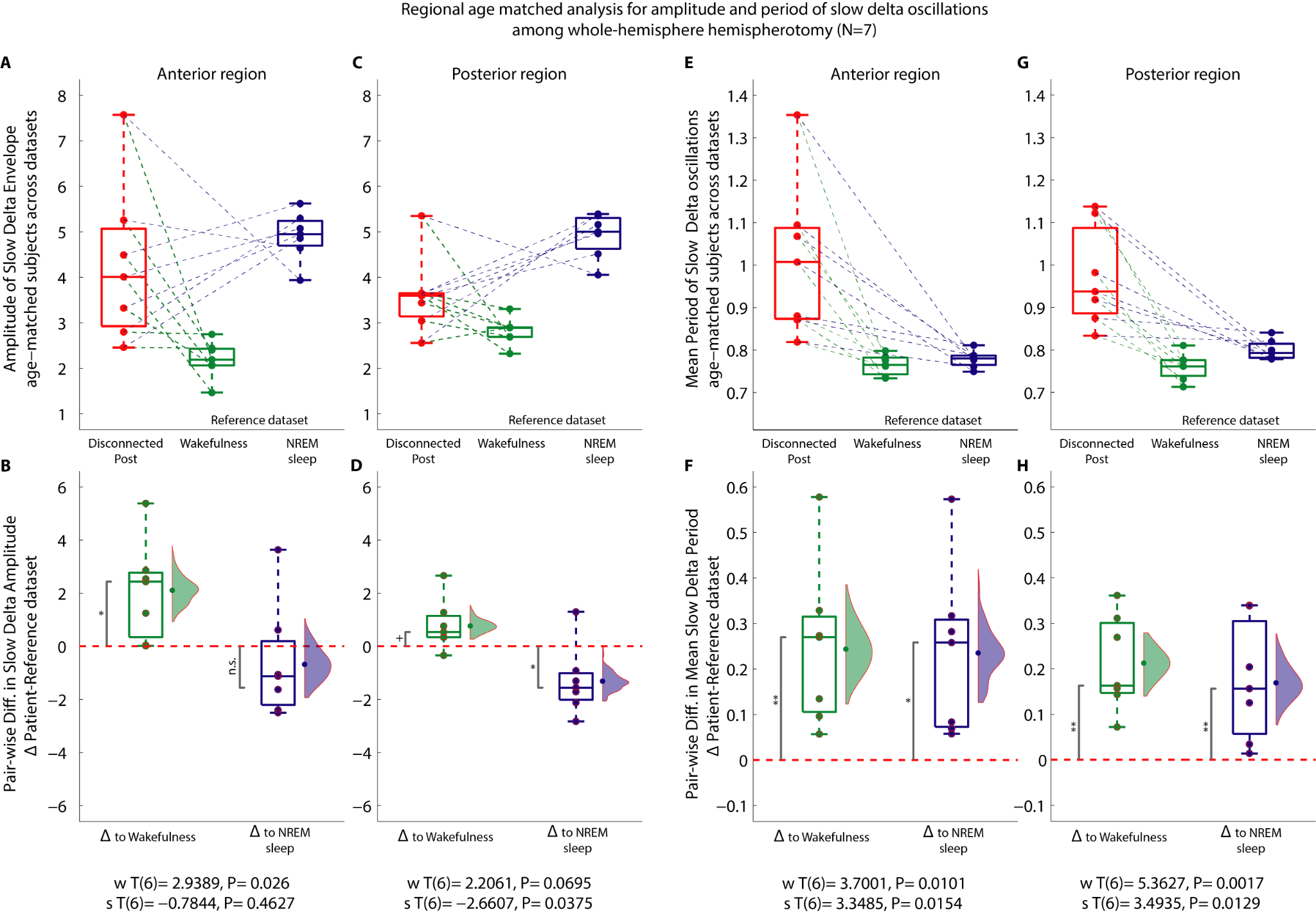
*

**Supplementary Figure Q. Regional age-matched analysis of amplitude and period of Slow Delta oscillations following whole-hemisphere hemispherotomy (N=7) in relation to the pediatric reference dataset across wake and NREM sleep.** (A–D) Slow Delta envelope amplitude comparison between the patient and reference datasets. (A,C) Boxplots show amplitude values for the disconnected cortex compared to the reference pediatric dataset during wakefulness and NREM sleep, separately for anterior (A) and posterior (C) regions. (B,D) Corresponding pairwise differences relative to wakefulness and NREM sleep, after age-matching. (E–H) Mean period of Slow Delta oscillations comparison between the patient and reference datasets. (E,G) Boxplots show period values for the disconnected cortex compared to the reference dataset during wakefulness and NREM sleep, separately for anterior (E) and posterior (G) regions. (F,H) Corresponding pairwise differences after age-matching.
Consistent with the main findings, both anterior and posterior disconnected regions showed intermediate amplitude values between wakefulness and NREM sleep; extending the main findings this analysis revealed that the anterior region more closely resembled NREM sleep reference values. Consistent with the main findings, both anterior and posterior regions exhibited Slow Delta periods substantially longer than those observed during wakefulness and NREM sleep. The data underlying this figure are provided in the file FigS17_Data, publicly available in the repository at the following <https://doi.org/10.5281/zenodo.16936516>

Overall, the amplitude of the Slow Delta envelope of the disconnected cortex showed intermediate values between those of wakefulness and NREM (N2 and N3 jointly considered), for both anterior and posterior regions (**Supplementary Figure Q, panel A,C**), in line with the main results obtained considering the median values over the whole disconnected cortex (**Figure 4A**). Specifically, age-matching revealed that the anterior disconnected cortex showed amplitude values significantly larger than those of wakefulness (T(6)=2.939, p = 0.026, with 7/7 subjects showing a consistent pairwise difference) and not significantly different from those of NREM sleep (T(9)=-0.764, p = 0.463, with 5/7 subjects showing a consistent pairwise difference), **Supplementary Figure Q, panel B.**Moreover, age-matching revealed that the posterior disconnected cortex showed larger amplitude values than those of wakefulness, albeit only with a non significant trend (T(6)=2.206, p = 0.0695, with 6/7 subjects showing a consistent pairwise difference), and significantly smaller than those of NREM sleep (T(6)=-0.764, p = 0.463, with 6/7 subjects showing a consistent pairwise difference), **Supplementary Figure Q, panel D.**

**S13.2 - Postero-Anterior specificities of Slow Delta Period**

Further, the period of Slow Delta oscillations of the disconnected cortex showed values higher than those of both wakefulness and NREM, for both anterior and posterior regions (**Supplementary Figure Q, panel E,G**), in line with the main results obtained considering the median values over the whole disconnected cortex (**Figure 4 B**).

Specifically, age-matching revealed that the anterior disconnected cortex showed period values significantly larger than those of both wakefulness (T(6)=3.7, p = 0.01, with 7/7 subjects showing a consistent pairwise difference) and NREM sleep (T(6)=3.349, p = 0.0154, with 7/7 subjects showing a consistent pairwise difference), **Supplementary Figure Q, panel F.**

Similarly, age-matching revealed that the posterior disconnected cortex showed period values significantly larger than those of both wakefulness (T(6)=5.363, p = 0.0017, with 7/7 subjects showing a consistent pairwise difference) and NREM sleep (T(6)=3.494, p = 0.0129, with 7/7 subjects showing a consistent pairwise difference), **Supplementary Figure Q, panel H.**

In sum, while both anterior and posterior regions of the disconnected cortex showed values of the amplitude of the Slow Delta envelope intermediate between wakefulness and NREM sleep, age-matching revealed that the anterior region showed values that resembled NREM sleep reference values more closely than the posterior region. Moreover, both anterior and posterior regions of the disconnected cortex showed values of the period of Slow Delta oscillations that substantially exceeded those of both wakefulness and NREM sleep, in both the anterior and posterior regions. This age-matched regional analysis expanded the analysis presented in **Figure 4** (Mean amplitude of the Slow Delta envelope and period of Slow Delta oscillations in the disconnected hemisphere, compared with respect to wakefulness and NREM sleep in the reference pediatric sample), revealing postero-anterior specificities in slow wave amplitude within the disconnected cortex, that could not be ascribed to an age-related effect. We reason that such postero-anterior differences in the amplitude of the Slow Delta envelope may be related to the specific structural patterns—particularly the location and extent of the lesions. In particular, post-ischemic damage may contribute to a reduced EEG signal amplitude.

**S14 - Sigma oscillations and spindle activity**

To determine whether the disconnected cortex (recorded when the patients were behaviourally awake) was devoid of spindle oscillations, a trained neurologist first inspected the EEG traces. Despite the abundance of slow oscillations resembling a sleep-like pattern, spindle grapho-elements were not identified and only sporadic, low-amplitude alpha (and not sigma) activity could be identified in the disconnected cortex, in line with the established thalamic origin of sleep spindles (Luthi, 2014; Fernandez and Luthi, 2020). Subsequently, quantitative spectral and temporal analyses were devoted to confirm this qualitative finding.

**S14.1 - Power of Sigma Periodic activity**

We aimed to estimate the portion of the PSD in the sigma band (~11-15 Hz, Purcell et al., 2017) generated by spindle activity, recognising that a strongly rhythmic signal contributes to a distinct narrow-band PSD peak, that broad-band changes can bias narrow-band PSD estimates, and finally, taking into account that the sigma band overlaps with the alpha and beta bands. Specifically, changes in broad band activity could differently affect the estimates of sigma power between conditions: the disconnected cortex displayed a notable broad-band rotation of the PSD and increase in low-frequency power following surgical disconnection (similarly to what was observed in the physiological wake-sleep transition, **Supplementary Figure R, panel A, B**), thus affecting the amount of narrow-band total power in the sigma band. Since the PSD of the EEG signal originates from a mixture of aperiodic and periodic activity, it can be accordingly decomposed as a sum of aperiodic and periodic PSD components (Palva and Palva, 2018; Donoghue et al., 2022), by modelling the aperiodic component as a 1/f-like distribution. Thus, to estimate the power of sigma activity corresponding to genuinely periodic activity (i.e. spindles), we considered only the power that exceeded the 1/f-like aperiodic trend (i.e. the positive PSD residuals after subtracting the 1/f-trend from the total PSD, **Supplementary Figure R, insets of panel A, B**).

During physiological sleep, the PSD displays a clear peak in the sigma band, a hallmark of the presence of spindles (**Supplementary** **Figure R, panel B**). Yet, the sigma band (~11-15 Hz, see Purcell et al., 2017) overlaps with both the alpha (~ 8-12 Hz) and beta band (~ 12-30 Hz) (**Supplementary Figure R, inset of panel B**). Thus, we restricted the sigma frequency range of interest to a band where the grand-average periodic PSD of sleep exceeded that of wakefulness in the reference sample, to obtain a more sleep-specific estimate of sigma power, with reduced influence from wake-like activity.

We thus estimated the amount of periodic sigma power by averaging the PSD in a sleep-specific sigma band, as determined from the overall sleep-wake difference **(**11.5-15.5 Hz, **Supplementary** **Figure R, inset of panel B)**. The strength of sigma oscillations – as defined above – in the disconnected cortex was then compared to that of sleep and wakefulness in the reference sample. We expected the disconnected cortex would show values considerably lower than those of sleep, and compatible with (or lower than) those of wakefulness.
Moreover, in the **Supplementary Material, section SN.2** we detected bursts of sigma oscillations, following a time-domain approach commonly employed in spindle detection during sleep (Ferrarelli et al., 2007; Andrillon et al., 2011; Sarasso et al., 2014; Hahn et al., 2020). If the properties of sigma bursts (such as their rate and amplitude) in the disconnected cortex differed significantly from those observed during physiological sleep and resemble those seen during physiological wakefulness instead – where spindles are notably absent – this would further support the notion that spindles are also absent in the disconnected cortex.

**
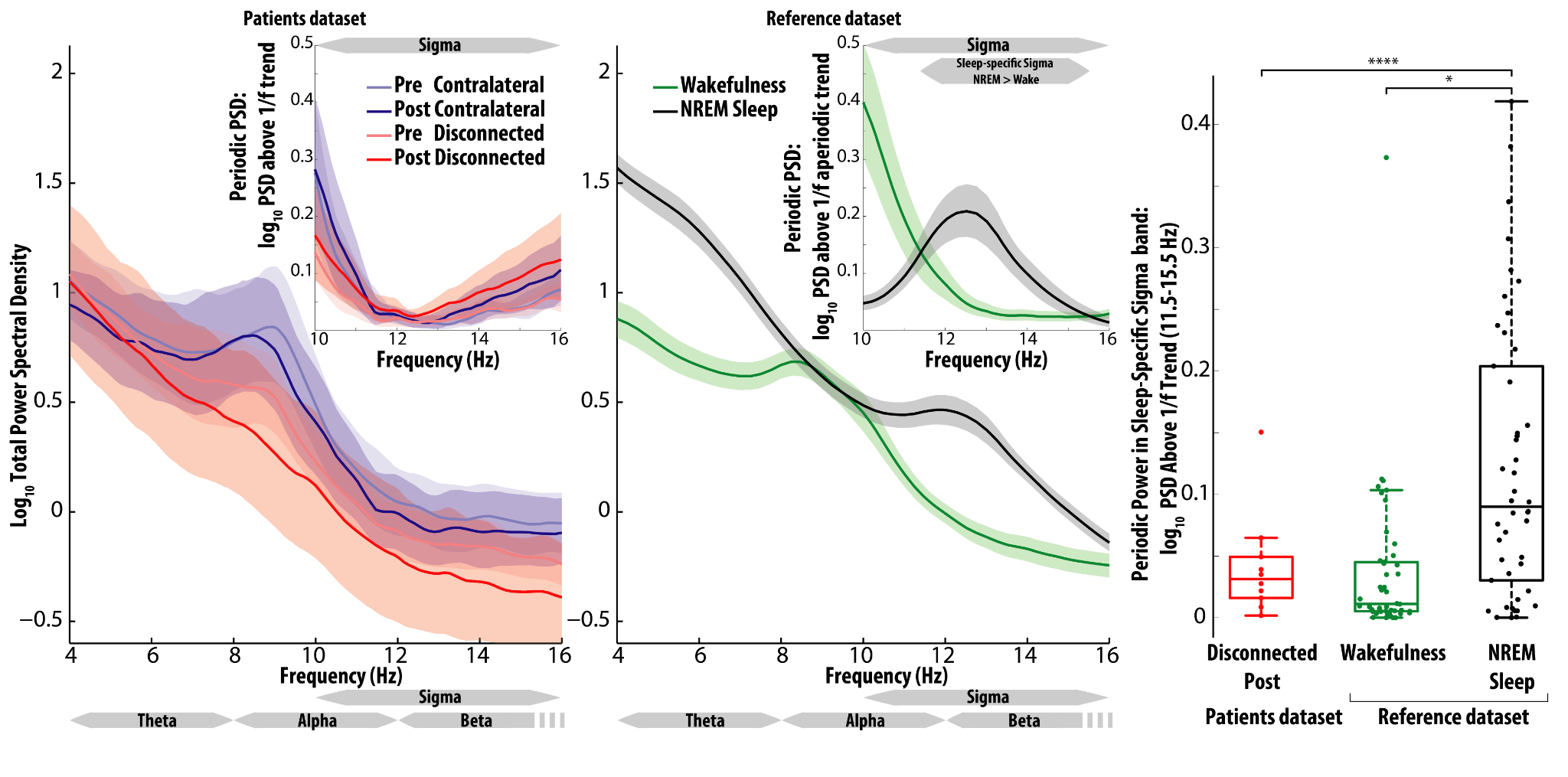
**

**Supplementary Figure R. Analysis of total and periodic PSD in the sigma band reveal the absence of rhythmic spindle activity in the disconnected cortex.**

**A)** Grand-average PSD in the patient dataset is shown for the disconnected and contralateral cortex, before and after surgery. A broad range of frequencies (4-16 Hz) spanning theta, alpha, and the lower range of the beta band is shown to provide a context for the PSD in the sigma band. The median values across channels is obtained, then the mean values across subjects is shown, along with its bootstrapped confidence interval. The inset displays the periodic PSD component, i.e. the PSD exceeding above the 1/f-like trend (displayed in **Figure 2**), within the sigma band (10-16 Hz). While the disconnected cortex displayed predominantly broad-band aperiodic activity with no clear peaks in the total PSD, its periodic PSD component displayed residual power pertaining to the neighboring alpha and beta bands; however no sigma peak occurred within the sigma band. **B)** Grand-average PSD is shown for physiological Wakefulness and NREM sleep (stages N2 and N3 combined) obtained from the pediatric cohort of the reference dataset. In the inset, the periodic PSD component of Wakefulness displays a downward inflection from the alpha band, while that of sleep displays a clear peak centered in the sigma band–reflecting spindle rhythmic activity. A thick grey line highlights the sleep-specific sigma band (11.5-15.5 Hz), where the grand-average periodic sigma PSD of NREM Sleep exceeds that of Wakefulness. **C)** The mean periodic component of the PSD in the sleep-specific sigma band (11.5-15.5 Hz), median across channels, is shown for each subject. The disconnected cortex showed values overlapping and consistent with those of Wakefulness, and substantially lower than those of NREM sleep. The data underlying this figure are provided in the file FigS18_Data, publicly available in the repository at the following <https://doi.org/10.5281/zenodo.16936516>

**SN.2 - Sigma burst detection**

After qualitative visual inspection of the EEG traces by a trained neurologist, and quantitative spectral analysis disentangling genuine oscillatory PSD from its aperiodic counterpart, we employed a time-domain approach to detect sigma bursts. This was done to further consolidate the absence of spindles in the disconnected cortex.

To achieve this, we applied a standard method for detecting sleep spindles to our specific context: in recordings of the disconnected cortex in behaviourally awake patients, and in recordings of a healthy pediatric cohort during both wakefulness and sleep.

The rate of sigma burst during sleep would provide a reference indicative of genuine spindle activity. On the other hand, since true spindles do not occur in healthy children during wakefulness, this state would provide a reference distribution for conditions where only spurious sigma bursts occur.

First, to estimate the amplitude of sigma oscillations, the EEG signal was band-pass filtered (IIR forward and reverse Butter filter, 5th order, cutoff set at 10-16 Hz), the absolute value of the Hilbert transform was obtained, and its time-course was temporally smoothed with a 200 ms moving average. From this signal, two adaptive thresholds were determined, for each subject and channel: an upper threshold (mean + 3 standard deviations) necessary to determine the presence of a sigma burst, and a lower threshold (mean + 1.5 standard deviations) to determine the duration of the sigma burst. Specifically, if the signal exceeded the upper threshold, a sigma burst was marked, and the sigma burst duration was considered as the lifespan where the signal exceeded the lower threshold in the surrounding time points **(Supplementary Figure S, panel A)**. Sigma bursts with a duration lower than 0.5 seconds and exceeding 3 seconds were discarded, since sleep spindles extend few oscillatory cycles and are short-lived. Finally, the rate of sigma bursts per minute was considered, as well as the mean amplitude within sigma bursts, averaged across sigma bursts. For statistical purposes, the median value across channels was obtained.

As expected, the sigma burst rate excellently discriminated wakefulness from NREM sleep (T(45)=16.7219, P=6.2394e−21), however, spurious sigma bursts were also observed in wakefulness, since alpha bursts have a frequency range that extends into that of sigma (**Supplementary Figure S, panel B**). The disconnected cortex displayed a sigma burst rate similar to that of wakefulness (T(54)=0.54047, P=0.5911), and substantially lower than that of sleep (T(54)=8.0062, P=9.6385e−11 ).

Similarly, the mean sigma burst amplitude well discriminated wakefulness from NREM sleep ( T(45)=9.1667, P=7.381e−12), however lower amplitude signal excursion in a frequency band extending in the sigma range were also observed in wakefulness (**Supplementary Figure S Sigma Burst, panel C**). The disconnected cortex displayed a mean sigma burst amplitude even lower than that of wakefulness (T(54)=2.633, P=0.011015), and substantially lower than that of sleep (T(54)=5.7994, P=3.5692e−07).

Hence, this analysis revealed that the disconnected cortex only generated few, small and short-lived energy bursts in the sigma band, whose rate and amplitude was substantially lower than those of sleep (about 4 times and 2 times lower respectively, **Supplementary Figure S)**; further, the rate was similar to that observed during normal wakefulness, and with even lower amplitude, consistent with the absence of any true spindle activity.

Let’s consider the occasional, small sigma bursts of uncertain/spurious origin obtained in the disconnected cortex and during wakefulness. In such conditions, aperiodic activity and alpha-beta oscillations filtered in the sigma band yielded sporadic bursts with a similar duration and frequency content to those of spindles. More generally, the EEG filtered in any narrow band displays a fat tailed distribution underlied by occasional high-amplitude bursts–due to the presence of fluctuating broad-band aperiodic activity, or by the waxing and waning of oscillatory activity in neighboring frequency bands–regardless of whether genuine oscillations exist in the unfiltered EEG, which are marked by a distinct peak in the PSD.

Overall, in the disconnected cortex the absence of any sleep-like sigma peak in the PSD (**Supplementary Figure R**), concurrently with the presence of occasional wake-like bursts of energy in the sigma band, highly different from those of sleep, confirmed that the disconnected cortex did not display any sleep spindles.

This negative finding in the disconnected cortex wells align with the established thalamic origin of spindles (Luthi, 2014; Fernandez and Luthi, 2020): since the disconnected cortex lacks thalamic input, it would be unable to support spindle activity.


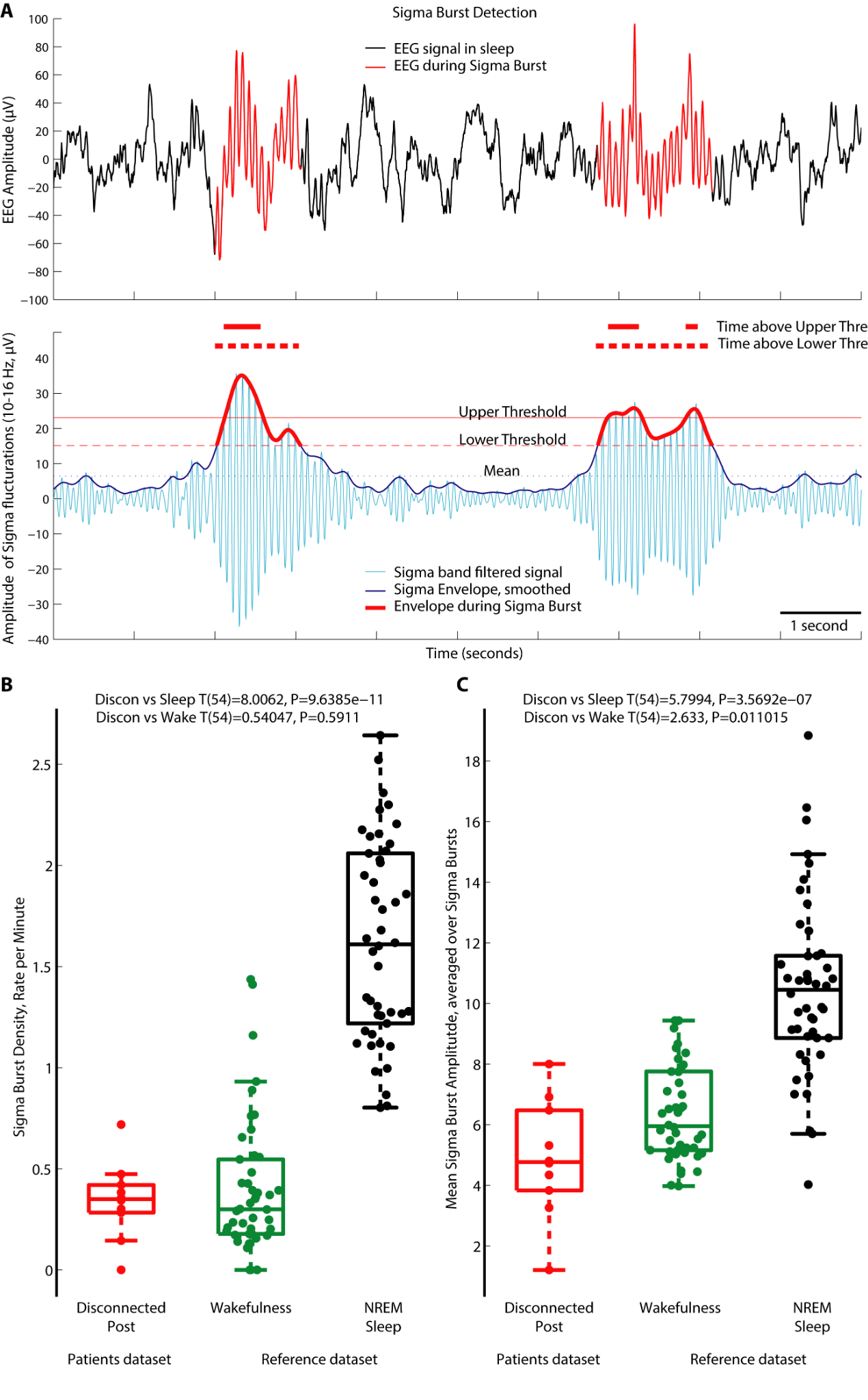


**Supplementary Figure S. Schematic depiction of the method employed for sigma bursts detection in a sleep recording, and observation of wake-like sigma burst properties in the disconnected cortex. A)** The EEG signal was filtered (10-16 Hz), its amplitude envelope was obtained, and subsequently temporally smoothed. A double threshold approach was employed for sigma burst detection: an upper threshold to assess the presence of a sigma burst, and a lower threshold to determine its duration. A sigma burst was confirmed if its duration lasted between 0.5 and 3 seconds. Here, representative EEG data of a healthy pediatric patient (12 y.o., male) during NREM sleep is shown, displaying two detected spindles. The properties of sigma bursts detected in the disconnected cortex are then compared to those obtained in the reference pediatric cohort during Wakefulness and NREM Sleep. These properties consist of: **B)** Sigma burst rate, and **C)** Mean Sigma Burst Amplitude. The data underlying this figure are provided in the file FigS19_Data, publicly available in the repository at the following <https://doi.org/10.5281/zenodo.16936516>

**SN.3 - Absence of spindles in the disconnected cortex**

During physiological NREM sleep, spindle activity, due to its narrow-band oscillatory property, is marked by a pronounced PSD peak in the sigma frequency band. The PSD of the disconnected cortex did not display any peak in the sigma band, similar to the reference sample during wakefulness (**Supplementary Figure R, panel A, B**).

We then extracted the periodic PSD component, by considering only the PSD that exceeded the 1/f-like aperiodic trend (shown in **Figure 1,** inset of panel A, **Figure 2**, **inset of panel B**), to better disentangle signs of periodic activity in the sigma frequency band generated by potential spindles. In the disconnected cortex, the periodic PSD component (i.e. that exceeding the aperiodic 1/f background) in the 10-16 Hz band originated from the upper-end of the alpha band and from the lower end of the beta band **(Supplementary Figure R, inset of panel A)**. The presence of genuine periodic activity in the sigma band, as marked by a distinct sigma peak, could thus be ruled out at the group level. This was corroborated by the inspection of individual periodic PSD components.

To obtain a marker specific for sleep-like sigma activity, we subsequently considered the portion of the sigma band that was specific for sleep spindles, i.e. whereby the periodic PSD in the sigma band of sleep exceeded that of wakefulness. The grand-average periodic sigma power of NREM sleep exceeded that of wakefulness in the 11.5-15.5 Hz range in the reference dataset (**Supplementary Figure R, inset of panel B**); we accordingly considered the mean periodic sigma power in this sleep-specific sigma band. In the disconnected cortex, the mean periodic power in the sleep-specific sigma band was remarkably lower than that of NREM sleep and similar to that observed during physiological wakefulness. Hence, spindle activity, clearly marked by pronounced periodic sigma power during physiological sleep, was absent in the disconnected cortex, similar to what was observed during physiological wakefulness.

Overall, the absence of any sleep-like sigma peak in the PSD **(Supplementary Figure R)**, concurrently with the presence of occasional bursts of energy in the sigma band similar to those of wakefulness, showed that the disconnected cortex did not display any sleep spindles. All the above quantitative analyses confirmed the visual assessment made by the neurologist.

**Supplementary references**

Andrillon T, Nir Y, Staba RJ, Ferrarelli F, Cirelli C, Tononi G, et al. Sleep spindles in humans: insights from intracranial EEG and unit recordings. J Neurosci. 2011;31(49):17821–34. <https://doi.org/10.1523/JNEUROSCI.2604-11.2011> PMID: 22159098

Avvenuti G, Handjaras G, Betta M, Cataldi J, Imperatori LS, Lattanzi S, et al. Integrity of corpus callosum is essential for the cross-hemispheric propagation of sleep slow waves: a high-density EEG study in split-brain patients. J Neurosci. 2020;40(29):5589–603. <https://doi.org/10.1523/JNEUROSCI.2571-19.2020> PMID: 32541070

Benjamini Y, Hochberg Y. Controlling the false discovery rate: a practical and powerful approach to multiple testing. J Roy Stat Soc Ser B Stat Methodol. 1995;57(1):289–300. <https://doi.org/10.1111/j.2517-6161.1995.tb02031.x>

Cohen MX. Analyzing neural time series data. The MIT Press; 2014. <https://doi.org/10.7551/mitpress/9609.001.0001>

Donoghue T, Schaworonkow N, Voytek B. Methodological considerations for studying neural oscillations. Eur J Neurosci. 2022;55(11–12):3502–27. <https://doi.org/10.1111/ejn.15361> PMID: 34268825

Fernandez LMJ, Lüthi A. Sleep spindles: mechanisms and functions. Physiol Rev. 2020;100(2):805–68. <https://doi.org/10.1152/physrev.00042.2018> PMID: 31804897

Ferrarelli F, Huber R, Peterson MJ, Massimini M, Murphy M, Riedner BA, et al. Reduced sleep spindle activity in schizophrenia patients. Am J Psychiatry. 2007;164(3):483–92. <https://doi.org/10.1176/ajp.2007.164.3.483> PMID: 17329474

Gao R. Interpreting the electrophysiological power spectrum. J Neurophysiol. 2016;115(2):628–30. <https://doi.org/10.1152/jn.00722.2015> PMID: 26245320

Hahn MA, Heib D, Schabus M, Hoedlmoser K, Helfrich RF. Slow oscillation-spindle coupling predicts enhanced memory formation from childhood to adolescence. Elife. 2020;9:e53730. <https://doi.org/10.7554/eLife.53730> PMID: 32579108

Lendner JD, Helfrich RF, Mander BA, Romundstad L, Lin JJ, Walker MP, et al. An electrophysiological marker of arousal level in humans. Elife. 2020;9:e55092. <https://doi.org/10.7554/eLife.55092> PMID: 32720644

Lüthi A. Sleep spindles: where they come from, what they do. Neuroscientist. 2014;20(3):243–56. <https://doi.org/10.1177/1073858413500854> PMID: 23981852

Purcell SM, Manoach DS, Demanuele C, Cade BE, Mariani S, Cox R, et al. Characterizing sleep spindles in 11,630 individuals from the National Sleep Research Resource. Nat Commun. 2017;8:15930. <https://doi.org/10.1038/ncomms15930> PMID: 28649997

Sarasso S, Proserpio P, Pigorini A, Moroni F, Ferrara M, De Gennaro L, et al. Hippocampal sleep spindles preceding neocortical sleep onset in humans. Neuroimage. 2014;86:425–32. <https://doi.org/10.1016/j.neuroimage.2013.10.031> PMID: 24176868

Smith SJ, Andermann F, Villemure JG, Rasmussen TB, Quesney LF. Functional hemispherectomy: EEG findings, spiking from isolated brain postoperatively, and prediction of outcome. Neurology. 1991;41(11):1790–4. <https://doi.org/10.1212/wnl.41.11.1790> PMID: 1944910

|  | |  |
| --- | --- | --- |
|  |  |  |
