# Supplementary material for: Hemispherotomy leads to persistent sleep-like slow waves in the isolated cortex of awake humans
Source: PLoS Biol. 2025 Oct 16;23(10):e3003060. doi: 10.1371/journal.pbio.3003060 (PMC12530565; doi:10.1371/journal.pbio.3003060)
Supplement: Attachment — Submitted filename: Response to Reviewers_revision.docx [file pbio.3003060.s002.docx]

**REBUTTAL LETTER**

Reviewer #1
**General comment and recommendation**

This work by Colombo, Favaro and colleagues presents an interesting investigation of a fascinating patient population, and asks a timely question: Do we have reason to believe a disconnected hemisphere may support consciousness despite being isolated from the world (including the rest of the body and brain)? The authors use methods and measures that are as well suited to answer the question as any we currently have available, and carry out their analyses meticulously. Their results are well presented and the article (largely) is written in an understandable and straightforward manner. That said, I find the conclusions drawn and the interpretations provided to be lacking. They seem to take their findings to be evidence against the presence of consciousness, while it seems a more prudent conclusion is that it does not provide (strong) evidence for the presence of (wakelike, rich, vivid, narrative etc) consciousness we are used to in normal waking life. To support their conclusion, they repeatedly claim that conditions such as sleep and anesthesia are unconscious, even though these are well known to be associated with dreams (albeit often vague or strange ones). Furthermore, the manuscript seems lacking in its presentation of the EEG literature on the relevant patient population and appears to present itself as the first of its kind. While it may very well be novel in the particular question it poses, it is by no means the first of its kind more broadly speaking wrt the methodology and patient population. In sum, I think the manuscript definitely deserves a chance of being published, but I strongly recommend rather extensive revision.

>> We thank the reviewer for his thoughtful and constructive feedback. We particularly appreciate the recognition of the interest and relevance of the patient population and the central question addressed by our study. We have carefully and extensively revised the manuscript to address the reviewer’s concerns which were organized along three levels (general comments, main comments and detailed comments). To facilitate the readability of our response, we follow the same organization as follows. First we provide a high-level **overview of the changes** undertaken, then we address the reviewer’s **main comments** (**a.1 to a.6**), finally we address in a detailed manner each and every comment raised by the reviewer (detailed **comments 1 to 33**).

**Overview of changes prompted by reviewer 1**

Steering away from a simplistic binary distinction between conscious and unconscious states, we refrain from categorizing conditions such as sleep and anesthesia as entirely unconscious. Instead, we adopt a graded and more nuanced perspective, recognizing that although an EEG dominated by slow-wave activity is widely accepted as a clinical marker of reduced consciousness, it does not necessarily indicate a complete loss of awareness (Fröhlich et al., 2021; Sevenius Nilsen et al., 2022).

In line with this view, we explicitly state in the Discussion that our findings do not allow us to definitively rule out the presence of conscious experience in the isolated hemisphere. Rather, we interpret the data as indicative of a unihemispheric sleep-like state intruding into wakefulness, and provide a detailed analysis of its similarities and differences with physiological sleep, including a discussion of the potential underlying mechanisms.

Accordingly, we propose that the observed state is consistent with diminished or absent conscious experiences, without claiming conclusive proof of unconsciousness. This interpretation is grounded in the observation that the isolated cortex displays a unihemispheric steep spectral profile, which shares key features with patterns observed in general anesthesia and the vegetative state in adults, and—upon direct comparison—closely matches that seen during deep NREM sleep in neurotypical children.

Furthermore, we have thoroughly reviewed the existing literature on EEG changes in cortica**l** disconnections. We confirm that our study represents the first exploration of the non-epileptic EEG background of the isolated cortex in awake human patients. We have nonetheless extended the description of previous literature in the introduction, highlighting that it was primarily concerned with assessing epileptic activity for clinical purposes (prognosis, patient selection), or performed in hemispherectomy (where the hemisphere is removed), or during anesthesia, which does not allow any reliable consciousness assessments.

Moreover, we have strengthened, as suggested, the rationale linking slow wave activity to cortical disconnection in the introduction, and clarified our aims and hypotheses.
Finally, we have moved the section on spindles to the Supplementary Material, as also suggested by **reviewer 3**.

**Main comments**

**a.1)** In particular, the introduction needs to more properly cover the literature on how EEG is affected by epilepsy and hemispherotomy (independent of whether the comparisons are done within subjects from pre to post surgery)
>> We appreciate the reviewer’s suggestion, which prompted us to more clearly contextualize our study in relation to prior literature on EEG alterations in epilepsy and hemispherotomy. This led to a deeper revision of the literature, confirming the novelty of the present study that we now thoroughly highlight in both the introduction and discussion sections. See answers to detailed **comments 3,** and **7**.

**a.2)** alternative measures of consciousness should be discussed and justifications for the choice made here should be improved,
>> We have extensively revised the Introduction to better justify our selection of EEG markers for assessing cortical slowing, from both narrow-band and broad-band perspectives. Specifically, we emphasize the empirical and theoretical rationale for focusing on slow oscillations—given their prominence following cortical deafferentation and their established role in clinical evaluations of consciousness (e.g., in anesthesia and disorders of consciousness)—and on the spectral exponent, a robust marker of the level of consciousness across physiological, pharmacological, and pathological conditions. Importantly, we have systematically studied this measure in a pediatric population across sleep and wakefulness (Favaro et al., 2023), providing a meaningful reference framework for the context of hemispherotomy. See answers to detailed **comments 8, 9** and related **comment 6**.

**a.3)** the (pre-stated) hypotheses must be more concretely formalized to give a better understanding of which analyses were planned ahead of seeing the data,

>> We thank the reviewer for this important point. In response, we have clarified and strengthened the study’s rationale in the revised Introduction and formalized clear hypothesis in the Methods sections. See answers to detailed **comments 8** and **10**.

**a.4)** a more proper discussion of the presence of conscious experiences in (so-called) "unconscious" states must be provided and used to contextualize what we can say about the presence of "islands of awareness" in this population (and a concrete definition of this phrase must be given—are dreams sufficient?),

>> Acknowledging the reviewer’s important point, we have thoroughly revised the manuscript to move away from a binary (ON/OFF) framing of consciousness and now describe deep sleep and general anesthesia as states of **absent or *reduced*** consciousness, rather than completely and unequivocally unconscious, as pointed out in the discussion paragraph **Limitations and Future directions** (Frohlich et al., 2021; Blackburne et al., 2024; Siclari et al., 2013; Siclari et al., 2017; Nir and Tononi, 2010; Sanders et al., 2012; Bonhomme et al., 2019; Sevenius Nilsen et al., 2022).

We have also clarified the meaning of “islands of awareness,” defining it more explicitly as the potential for internally generated experiences (e.g., dreams) within disconnected cortical regions. While we cannot entirely rule out such experiences, we now discuss that our findings are consistent with potentially absent or reduced conscious experiences and with a reduced probability of vivid dream-like experiences  rather than constituting definitive evidence against any form of conscious experience.

This perspective is clearly articulated in the revised version of the dedicated discussion paragraph. See answers to detailed **comments 5, 21, 25, 27, 32.**

**a.5)** a reevaluation of the need for the "spindle analysis" is required, as it currently takes up too much space for my liking
>> In response to the reviewer’s suggestion, we have moved all spindle-related analyses and figures to the Supplementary Material to streamline the main text. See answers to detailed **comments 13** and **19**.

**a.6)** the phrasing of what this study actually gives evidence for (and not) must be improved (i.e. is this evidence AGAINST, or LACK of evidence FOR, islands of awareness (and why)?).
>> We have refined the phrasing throughout the manuscript to clarify that our findings do not support the hypothesis that hemispherotomy may result in cortical ‘islands of awareness’ (detailed **comment 26**). Rather, our findings support the inference that the disconnected cortex may not sustain vivid, dream-like experiences (detailed **comments 28, 31**)--based on the presence of prominent slow waves, and on a steep spectral decay, closely aligned with deep NREM values.

In sum, our findings of a unihemispheric electrophysiological spectral profile resembling deep-sleep are consistent with absent, or reduced conscious experiences, rather than constituting definitive evidence against any form of conscious experience. This perspective is clearly articulated in the section **Limitations and Future directions.**

This interpretation aligns with the data and takes into account the more nuanced perspective highlighted by the reviewer. A detailed explanation follows in the detailed **comments 5, 26, 28, 29, 31**.

Below are all the comments I made for myself while reading through the manuscript. While all of them do not need explicit answers, they are hopefully useful for guiding the authors in their revisions. Best of luck!

Below, we reply extensively to all of the detailed comments. Along this rebuttal letter, our replies appear in blue, the quoted text of the manuscript (main or supplementary) appears in indented *italic* text as follows:

*Entirely new paragraphs are in italic.*

***title appears in bold, italic, and underlined,*** *where a whole new section has been added.*

*In the case of minor edits to some original text (in italic), ~~old removed content appears as strikethrough text~~, the new content is marked in italic, and underlined.*

**Detailed Comments**

**Title and highlights**

- **1)** The colon title makes it seem as though Hemispherotomy somehow is a cortical island. This is clearly not the intent. Perhaps adding a phrase like "evidence for" after the colon would help?

>> We appreciate the suggestion, although we agree it would be more explicit to use longer phrasing, we decided to keep the colon for conciseness

- **2)** The emphasis on "sleep-like", which is then immediately undermined by the lack of spindles etc seems odd to put in the title. Maybe rather emphasize slow waves or the steeper spectral slope? I'd recommend reworking the title.

>>We appreciate the reviewer’s suggestion to refine the title for greater specificity and clarity. The use of the term *"sleep-like"* was intended to convey a state resembling, but not identical to, physiological sleep—precisely due to its distinct features, such as the absence of spindles. We have further specified that the sleep-like only refers to the presence of slow wave activity and broad-band slowing, and have revised the title to:

**"Hemispherotomy: a cortical island of sleep-like slow waves ~~activity~~ in awake humans."**

**Abstract**

- **3)** Authors state that they explore "for the first time the electrophysiological state of the isolated cortex before and after [hemispherotomy]". This does not seem to be the case. A quick google search yielded what looked like many studies employing similar strategies (although not to answer the same question). E.g The significance of bilateral EEG abnormalities before and after hemispherectomy in children with unilateral major hemisphere lesions - PubMed and EEG lateralization and seizure outcome following peri-insular hemispherotomy for pediatric hemispheric epilepsy | Child's Nervous System. These are just two examples, from a fast search. I urge the authors to properly review the literature on this. I may well be misunderstanding, and be mistaken in that the author's claim is false, but it seem very surprising to me that no studies comparing EEG before and after hemispherotomy have ever been published.

>> We thank the reviewer for this comment, which offers an opportunity to clarify an important point in the section of the Introduction addressing prior literature. Let’s first consider a fundamental distinction between hemispherectomy and hemispherotomy. Hemispherectomy involves both functional disconnection and surgical removal of the pathological hemisphere (in whole or in part), whereas hemispherotomy entails disconnection without resection, preserving the structural and vascular integrity of the disconnected hemisphere (Lew, 2014; Pinto et al., 2014; de Ribaupierre & Delalande, 2008). While several previous studies have examined post-operative EEG in patients undergoing hemispherectomy, often focusing on residual epileptic activity or lateralization (including the example noted by the reviewer), these are conceptually and methodologically distinct from our investigation. In hemispherectomy, it is generally not possible to assess the electrophysiological state of the disconnected cortex, since the tissue is resected.
In contrast, hemispherotomy uniquely enables direct EEG recording from the disconnected cortex, offering a rare window into the neurophysiological state of an anatomically intact but functionally isolated hemisphere. The sole EEG studies conducted thus far that have investigated lateralized changes in background regional EEG activity in hemispherotomy patients were performed under general anesthesia, which restricts the capacity to explore markers of consciousness (Wennberg et al., 1997; Hawasli et al., 2017).

To the best of our knowledge, no previous study has systematically investigated the spontaneous, non-epileptic background EEG activity of the isolated cortex in awake pediatric patients, both before and after hemispherotomy. Unlike previous literature on the topic of hemispherotomy, our approach is not focused on epileptiform discharges or seizure outcomes, but rather on characterizing the background neurophysiological dynamics of the disconnected hemisphere. We accordingly specify in the revised **abstract**:

*We address these questions by exploring for the first time the electroencephalographic (EEG) ~~the electrophysiological~~ state of the isolated cortex during wakefulness before and after surgery in ten ~~awake~~ pediatric patients, focusing on non-epileptic background activity.*

Similarly, we now better specify the novelty aspects of our study in relation to previous studies in the revised **Introduction:**

*Here, we fill this gap by ~~studying for the first time the regional EEG patterns~~ analyzing the EEG background–i.e. free of epileptiform discharges–recorded during wakefulness in ten pediatric participants who had undergone a complete cortical disconnection, of either an entire hemisphere (~~complete hemispherotomy~~ in 7 cases) or a large portion of it (temporo-parieto-occipital disconnection in 3 cases).*

To avoid any confusion, we have clarified the distinction between studies on hemispherotomy and on hemispherectomy and between studies focusing on background and those focusing on epileptic EEG activity for clinical purposes (e.g. patient selection, prognosis) in the revised **Introduction**:

*The disconnected cortex is not surgically removed, as in hemispherectomy (Lew, 2014; Pinto et al., 2014), but is left inside the cranial cavity with preserved vascular supply (de Ribaupierre and Delalande, 2008), completely devoid of communication with the external environment – except for cortical afferents from the olfactory bulb, in some cases. Hemispherotomy, currently preferred over hemispherectomy due to fewer clinical complications (Karagianni et al., 2023), ~~This neurosurgical procedure~~* *is effective in blocking the spread of epileptic seizures from the pathological cortex to the rest of the brain, allowing patients to lead satisfying lives (Marras et al., 2010; Delalande et al. 2007; de Palma et al., 2019) [...]*

*One way to clarify this complex landscape would be to assess the background ~~regional~~ electrophysiological states of the brain of awake subjects both before and after hemispherotomy. Beyond older studies on the background EEG of the remaining hemisphere after hemispherectomy (Marshall and Walker, 1950; Cobb and Sears, 1960; Ravenna and Beneditti, 1965, Obrador et al., 1950) and of partially disconnected prefrontal lobe during lobotomy (Echlin et al., 1952; Henry and Scoville, 1952), the literature on EEG activity in patients with hemispherotomy focused primarily on interictal epileptiform activity and clinical outcome (Abraham et al., 2019; Greiner et al., 2011; Pepi et al., 2022; Alzahrany et al., 2022; Pollo et al., 2008; Limbrick et al., 2009; Marchi et al., 2019), proving crucial for patient selection and prognostic assessment (Lew, 2014; Kim et al., 2010; Lopez et al., 2021; Hartlieb et al., 2021). To date, the only  studies examining lateralized changes in regional EEG   ~~study conducted on background~~ activity in hemispherotomy patients were ~~was~~ performed under general anesthesia, which precludes reliable inferences about the state of consciousness ~~addressing this question~~ (Wennberg et al., 1997; Hawasli et al., 2017).*

Moreover, we found a recent case report that described intracranial EEG recordings after surgical disconnection of a portion of the frontal lobe (Joshi et al., 2025). This peculiar study was only possible given that the surgery was ineffective (which was an exclusion criterion in our case) and the patient required a second in-depth assessment. They observed localized burst suppression intracranially occurring under scalp EEG slow waves. Particularly, this case illustrates that scalp slow waves can coincide with mixed or dissociated intracranial patterns. We consider this single evidence, along with other evidence of different regional patterns of local cortical wakefulness occurring in a state of global sleep  (e.g., Nobili et al., 2012; Terzaghi et al., 2012) to enrich the discussion of future research avenues. Accordingly, we now report the following new text in the revised **Discussion**:

*Further, the slowing observed at the scalp level should be characterized with intracranial recordings—in cases in which clinical outcomes require postoperative invasive monitoring. Such exploration would be very informative in this context because slow waves recorded at the scalp level can mask the presence of specific regional patterns that may be relevant for consciousness. For example, intracranial recordings have shown that local wake-like intrusions can occur during NREM parasomnias, remaining largely undetected by scalp EEG (Nobili et al., 2012; Terzaghi et al., 2012). In contrast, a recent case report involving the surgical isolation of a portion of the frontal lobe (Joshi et al., 2025) revealed an EEG dominated by slow waves, along with a localized intracranial pattern of burst suppression—a hallmark of the deepest stages of anesthesia and profound coma (Steriade et al., 1994; Niedermeyer, 2009; Amzica 2009; Purdon et al., 2015).*

With regard to detailed arguments concerning the epileptological aspects, we refer to our response to the subsequent specific **comment 7** from the reviewer.

- **4)** This statement seems imprecise: " a broad-band shift in power spectral density from high to low frequencies." I assume the intent is to show the change in slope. But looking at the PSDs, the claim does not seem supported. There is an increase in low delta, and that's it**.** Consider rephrasing to explicitly mentioning the spectral slope.

**>>** The comment provides us an opportunity to clarify how the observed spectral changes are closely linked to a redistribution of the Power Spectral Density towards lower frequencies.

We first provide some general considerations. The EEG PSD typically follows a 1/f-like distribution, whereby lower frequencies dominate in power relative to higher ones. This spectral decay is a robust feature of electrophysiological signals across physiological, pharmacological, and pathological states [Shen et al., 2003; Miskovic et al., 2019; Bódizs et al., 2021; Bódizs et al., 2024; Zilio et al., 2021; Zilio et al., 2023; Schneider et al., 2022; Horváth et al., 2022; Alnes et al., 2023; Rosenblum et al., 2024; Colombo et al., 2019; Colombo et al., 2023; Maschke et al., 2023a; Maschke et al., 2023b; Casey et al., 2023], and becomes especially evident when muscular and ocular artifacts are minimized–which we specifically addressed in our study.

A steeper spectral decay—i.e., a more negative spectral exponent—reflects a relative redistribution of power from higher to lower frequencies, even if total power would remain constant. Importantly, this kind of broad-band redistribution can occur without corresponding significant changes in absolute power in narrow frequency bands, due to inter-individual variability in total power or spectral intercept [Donoghue et al., 2022]. Conversely, a narrow-band power increase (e.g., in Slow-Delta) can occur without a change in the spectral slope, when a spectral peak, exceeding the aperiodic trend, increases. Thus, narrow-band and broad-band spectral changes are related but dissociable phenomena.

In our data, both effects were present: a narrow-band increase in Slow-Delta power (**Fig. 2B**), and a steepening of the spectral slope (**Fig. 2C**), consistent with a more pronounced 1/f decay.

Specifically, the overall spectral decay of the PSD is evident in each of the conditions (**Fig 2A**). This decay follows a 1/f-like spectral trend, depicted in the inset in **Fig. 2** (peak-shaped deviations from the trend corresponding to periodic activity are depicted in **Supplementary Fig. 3)**. Accordingly, the disconnected hemisphere (thick red line in **Fig 2A**, reported below) exhibits an overall steepening of the aperiodic spectral trend compared to all other conditions.

(We report above an extract from **Figure 2A**)

Notably, the spectral exponent showed stronger and more consistent effects than Slow-Delta power—in terms of statistical evidence and individual consistency, for both the pre-post change and the asymmetry post-surgery. This is documented in **Supplementary Tables 1–2** and **Supplementary Figure 6.** The observed spectral changes entail a redistribution of power from higher to lower frequencies, in keeping with our original claims in the abstract.

See also answer to **comment 10**, for a discussion of how the inter-related narrow-band and broad-band changes entail a spectral redistribution.

To describe the observed findings with better clarity and specificity, we have updated the **abstract**:

*"Post-surgery, the isolated cortex exhibited prominent slow oscillations (<2 Hz) and a steeper broad-band spectral decay, entailing a redistribution of power towards lower frequencies ~~shift in power spectral density from high to low frequencies~~*

This revised formulation explicitly refers to the steepening of the spectral slope, as suggested by the reviewer, and avoids ambiguity regarding the nature of the spectral change. From a technical standpoint, we have also chosen to avoid the term ‘shift’, which could misleadingly imply a translation (e.g., an isolated change in offset), and instead use the more neutral term ‘redistribution’ in the abstract and in the main text. A more technical characterization— describing the observed effects as a clockwise rotation of the PSD trend in log-log space—is provided in the **Supplementary Material** (as noted in detailed **comment 10)**.

Alnes SL, Bächlin LZM, Schindler K, et al. Neural complexity and the spectral slope characterise auditory processing in wakefulness and sleep. *European Journal of Neuroscience*. Published online March 1, 2023. doi:10.1111/ejn.16203

Bódizs R, Szalárdy O, Horváth C, et al. A set of composite, non-redundant EEG measures of NREM sleep based on the power law scaling of the Fourier spectrum. *Sci Rep*. 2021;11(1). doi:10.1038/s41598-021-81230-7

Bódizs R, Schneider B, Ujma PP, et al. Fundamentals of sleep regulation: Model and benchmark values for fractal and oscillatory neurodynamics. *Prog Neurobiol*. 2024;234. doi:10.1016/j.pneurobio.2024.102589

Casey CP, Tanabe S, Farahbakhsh ZZ, et al. Evaluation of putative signatures of consciousness using specific definitions of responsiveness, connectedness, and consciousness. *Br J Anaesth*. Published online October 30, 2023. doi:10.1016/j.bja.2023.09.031

Colombo MA, Napolitani M, Boly M, et al. The spectral exponent of the resting EEG indexes the presence of consciousness during unresponsiveness induced by propofol, xenon, and ketamine. *Neuroimage*. 2019;189:631-644. doi:10.1016/j.neuroimage.2019.01.024

Colombo MA, Comanducci A, Casarotto S, et al. Beyond alpha power: EEG spatial and spectral gradients robustly stratify disorders of consciousness. *Cerebral Cortex*. 2023;33(11):7193-7210. doi:10.1093/cercor/bhad031

Donoghue T, Schaworonkow N, Voytek B. Methodological considerations for studying neural oscillations. *Eur J Neurosci*. 2022;55(11-12):3502-3527. doi:10.1111/ejn.15361

Horváth C, Szalárdy O, Ujma PP, et al. Overnight dynamics in scale-free and oscillatory spectral parameters of NREM sleep EEG. *Sci Rep*. 2022;12(1). doi:10.1038/s41598-022-23033-y

Maschke C, Duclos C, Owen AM, et al. Aperiodic brain activity and response to anesthesia vary in disorders of consciousness. *Neuroimage*. 2023a;275. doi:10.1016/j.neuroimage.2023.120154

Maschke C, O'Byrne J, Colombo MA, et al. Criticality of resting-state EEG predicts perturbational complexity and level of consciousness during anesthesia. Preprint. *bioRxiv*. 2023b;2023.10.26.564247. Published 2023 Oct 31. doi:10.1101/2023.10.26.564247

Miskovic V, MacDonald KJ, Rhodes LJ, et al. Changes in EEG multiscale entropy and power-law frequency scaling during the human sleep cycle. *Hum Brain Mapp*. 2019;40(2):538-551. doi:10.1002/hbm.24393

Rosenblum Y, Esfahani MJ, Adelhöfer N, et al. Fractal cycles of sleep: a new aperiodic activity-based definition of sleep cycles. *bioRxiv*. Published online January 1, 2024:2023.07.04.547323. doi:10.1101/2023.07.04.547323

Schneider B, Szalárdy O, Ujma PP, et al. Scale-free and oscillatory spectral measures of sleep stages in humans. *Front Neuroinform*. 2022;16. doi:10.3389/fninf.2022.989262

Shen Y, Olbrich E, Achermann P, et al. Dimensional complexity and spectral properties of the human sleep EEG. Electroencephalograms. *Clin Neurophysiol*. 2003;114(2):199-209. doi:10.1016/s1388-2457(02)00338-3

Zilio F, Gomez-Pilar J, Cao S, et al. Are intrinsic neural timescales related to sensory processing? Evidence from abnormal behavioral states. *Neuroimage*. 2021;226. doi:10.1016/j.neuroimage.2020.117579

Zilio F, Gomez-Pilar J, Chaudhary U, et al. Altered brain dynamics index levels of arousal in complete locked-in syndrome. *Commun Biol*. 2023;6(1). doi:10.1038/s42003-023-05109-1

- **5)** This claim is too strong: "challenging the possibility that hemispherotomy might lead to inaccessible "islands of awareness." Given that conditions with comparable slopes and EEG characteristics are commonly associated with dreams, there may very well still be "islands of awareness" (insofar as "awareness" is not taken to strictly be referring to conscious experiences of external stimuli or "process" data coming in from outside).
  >> We recognize the importance of avoiding overstatements, particularly given the complexity of detecting potential awareness in the impossibility of obtaining self-reports. In response, we have revised the original language to avoid categorical claims and now frame our interpretation more cautiously. Specifically, we write:

*The findings of prominent slow oscillations and broad-band slowing support inferences of absent, or reduced awareness in the isolated cortex*. *~~However, spindles did not emerge in the isolated cortex due to the lack of subcortical inputs, constituting a fundamental difference from physiological sleep. These findings demonstrate a unihemispheric sleep-like state during wakefulness,.challenging the possibility that hemispherotomy might lead to inaccessible "islands of awareness~~."*

This revised phrasing intentionally avoids categorical or definitive conclusions and instead reflects the limited scope of what can be inferred from EEG features alone. By using the formulation “support inferences”, we aim to acknowledge the uncertainty inherent in such conclusions—grounding them in empirical evidence, while recognizing that they do not necessarily exclude the possibility of residual or covert awareness. While the findings are consistent with absent or reduced awareness, the possibility of residual consciousness is explicitly acknowledged and further addressed in the discussion sections **“The problem of inaccessible awareness”** and **“Limitations and Future Directions”**.

**Introduction**

- **6)** You say, " More broadly, the activity patterns that extensive regions of the human brain can sustain following massive deafferentation remain unknown", but I feel some things Can be assumed given work on isolated cortical slices (e.g timofeev et al 2000 and Sanchez-Vives and McCormick 2000). I feel the assumption that slowing might be expected (e.g. given the senior author's recent perspective "Shaping the Default Activity Pattern of the Cortical Network") should be briefly discussed or introduced here to help the reader understand the context, and choices of measures applied.

**>>** We appreciate the reviewer’s suggestion, which allowed us to better contextualize our study within the broader literature on experimental deafferentation in animal models. Below, we report the revised text where we reference animal studies on cortical slabs and slices to support the rationale that cortical slowing is expected following deafferentation. Hereby an excerpt of the revised **Introduction**:

*First, converging evidence suggests that cortical deafferentation can lead to resting electrophysiological patterns dominated by prominent slow oscillations (around 1 Hz), resembling those typically observed during deep sleep. (...) Additionally, pronounced sleep-like slow oscillations represent the dominant activity pattern in experimental models of complete cortical disconnection (Sanchez-Vives et al., 2017), such as isolated cortical gyri in animal models (i.e., cortical slabs) (Timofeev et al., 2000) and cortical slices in vitro (Capone et al., 2019).*

To better appreciate the context for this passage, please see the answer to **comment  9.**

Sanchez-Vives M V., Massimini M, Mattia M. Shaping the Default Activity Pattern of the Cortical Network. Neuron. 2017;94(5):993-1001. doi:10.1016/j.neuron.2017.05.015

Timofeev I, Grenier F, Bazhenov M, et al. Origin of slow cortical oscillations in deafferented cortical slabs. Cereb Cortex. 2000;10(12):1185-1199. doi:10.1093/cercor/10.12.1185

Capone C, Rebollo B, Munoz A, et al. Slow waves in cortical slices: How spontaneous activity is shaped by laminar structure. Cerebral Cortex. 2019;29(1):319-335. doi:10.1093/cercor/bhx326

- **7)** The introduction goes on to claim that there is little to no relevant studies using EEG. I find this to be strange, as there is a long tradition of using EEG to monitor epilepsy patients, both for diagnosis, localization of seizure foci, prognosis for surgery, and evaluation of effectiveness of surgery. This literature needs to be covered in the introduction, or a strong argument for why it can be bypassed needs to be presented.

**>>** We thank the Reviewer for this comment, as it allows us to clarify the context of our study in relation to studies that focused on epileptic activity after hemispherotomy.

We fully acknowledge that epileptiform EEG activity in patients undergoing hemispherotomy has been extensively studied. In particular, several studies have investigated the pre-operative scalp EEG, focusing on bilateral abnormalities (Abraham et al., 2019; Limbrick et al., 2009) and lateralized epileptiform discharges (Greiner et al., 2011) and also pre-operative ictal SEEG (Marchi et al., 2019). Moreover, one recent study applied machine learning approaches to predict post-surgical seizure freedom (Pepi et al., 2022). Other works have examined the post-operative EEG, including the persistence of epileptiform discharges in the disconnected hemisphere (Alzahrany et al., 2022), sometimes even employing intraoperative EEG monitoring (Pollo et al., 2008; Kim et al., 2010). This strong focus on epileptic activity has been clinically driven, given its relevance for patient selection and prognostic assessment, as also emphasized by **Reviewer #2** (Lew, 2014; Lopez et al., 2021; Hartlieb et al., 2021).

However, despite the breadth of clinical research into epileptic discharges, the background EEG activity of the disconnected hemisphere—particularly following hemispherotomy—remains poorly characterized. Yet, this aspect offers valuable insight into the functional state of the isolated human cortex, beyond its epileptogenic potential.

In light of the reviewer’s comment, we have revised the **Introduction** to acknowledge this existing body of work and to clearly distinguish prior epilepsy-focused EEG studies from the aims of our investigation.

*Beyond older studies on the background EEG of the remaining contralateral hemisphere after hemispherectomy (Marshall and Walker, 1950; Cobb and Sears, 1960; Ravenna and Beneditti, 1965, Obrador et al., 1950) and of partially disconnected prefrontal lobe during lobotomy (Echlin et al., 1952; Henry and Scoville, 1952), the literature on EEG activity in patients with hemispherotomy focused primarily on interictal epileptiform activity and clinical outcome (Abraham et al., 2019; Greiner et al., 2011; Pepi et al., 2022; Alzahrany et al., 2022; Pollo et al., 2008; Limbrick et al., 2009; Marchi et al., 2019), aiming at patient selection and prognostic assessment (Lew, 2014; Kim et al., 2010; Lopez et al., 2021; Hartlieb et al., 2021). To date, the only ~~EEG  study conducted on background~~ studies examining lateralized changes in regional EEG activity in hemispherotomy patients ~~was~~ were performed under general anesthesia, which precludes reliable inferences about the state of consciousness ~~addressing this question~~ (Wennberg et al., 1997; Hawasli et al., 2017).*

**8)** The paragraph starting with "After hemispherotomy we observed…" seems to be a mix of results and discussion, before going back to justifying methodological choices without first clearly stating the aims and hypotheses of the study. While I understand what the authors are doing in this study, I think the introduction could be greatly improved.

>> We appreciate the reviewer’s input. We have thoroughly revised the introduction and clarified the aims and hypothesis of the study. Please see the detailed answer to detailed **comment 9**.

**9)** In addition to the missing discussion of EEG-studies in relevant patient populations, I think there should be a better introduction of available methods for assessing consciousness objectively using EEG, providing strong justification for why the two main measures were chosen.
>> We thank the reviewer for this insightful comment, which prompted us to revise and strengthen the Introduction section. The original statement reflected clinical impressions of EEG slowing after hemispherotomy, which we formally tested using narrow- and broad-band spectral measures widely used in consciousness research. In response to the reviewer’s suggestion, we have extensively restructured the Introduction to first articulate the rationale for investigating EEG slowing in the isolated cortex, before introducing methodological details or findings. This rationale is now better articulated in the revised **Introduction**. The revised section now reads:

*Specifically, we investigated whether hemispherotomy led to the emergence of slow wave activity and to an overall broad-band slowing in the isolated cortex. The rationale for this exploration rests on two fundamental considerations drawn from the literature on experimental models and clinical populations. First, converging evidence suggests that cortical deafferentation can lead to resting electrophysiological patterns dominated by prominent slow oscillations (around 1 Hz), resembling those typically observed during deep sleep. Local intrusions of sleep-like slow waves during wakefulness have been documented in the cortex following focal brain injuries that disrupt both ascending and lateral connections (Massimini et al., 2024). This phenomenon has been observed in both animal models (Gloor et al., 1977) and human patients (Nuwer et al., 1987). Additionally, pronounced sleep-like slow oscillations represent the dominant activity pattern in experimental models of complete cortical disconnection (Sanchez-Vives et al., 2017), such as isolated cortical gyri in animal models (i.e., cortical slabs) (Timofeev et al., 2000) and cortical slices in vitro (Capone et al., 2019).*

*Second, evaluating slow waves in the background EEG is a widely used and practical approach for assessing the level of consciousness in clinical settings. While the presence of slow waves may not necessarily indicate a complete loss of consciousness (Fröhlich et al., 2021; Sevenius Nilsen et al., 2022), a background EEG dominated by slow-wave activity is broadly recognized as a key factor in clinically and ethically significant decision-making. This includes critical applications such as the stratification of patients with disorders of consciousness (Estraneo et al., 2016; Schiff, 2016; Comanducci et al., 2020) and the adjustment of anesthetic depth during surgery (Ní Mhuircheartaigh et al., 2013; Warnaby et al., 2017).*

*To characterize background EEG activity before and after surgery, we employed not only a classical narrow-band spectral analysis (0.5-2 Hz) but also the spectral exponent, which reflects the broad-band 1/f-like decay of the Power Spectral Density (PSD). This quantitative measure offers several advantages in our context. First, by indexing the relative distribution of power across low and high frequencies, the spectral exponent is less influenced by potential reductions in cortical volume that could affect absolute EEG low-frequency power [Ní Mhuircheartaigh et al., 2013]. Second, research on serial awakenings during NREM sleep has shown that the likelihood of dream reports can be inferred from the balance between low- and high-frequency power (Siclari et al., 2017).*

*Most importantly, the spectral exponent has been validated as a reliable EEG marker of the level of consciousness across a range of physiological (Shen et al., 2003; Miskovic et al., 2019; Bódizs et al., 2021; Zilio et al., 2021; Schneider et al., 2022; Horváth et al., 2022; Alnes et al., 2023; Rosenblum et al., 2024; Bódizs et al., 2024), pharmacological (Zilio et al., 2021; Colombo et al., 2019; Maschke et al., 2023a; Maschke et al., 2023b; Zilio et al., 2023; Casey et al., 2023), and pathological states (Colombo et al., 2023; Maschke et al., 2023a; Zilio et al., 2023), including conditions of disconnected consciousness such as ketamine-induced dreaming and locked-in syndrome. Crucially, the spectral exponent has also been extensively studied in large pediatric samples, both during wakefulness (Favaro et al., 2023; Cellier et al., 2021; McSweeney et al., 2023; Schaworonkow and Voytek, 2021; Cross et al., 2024) and NREM sleep (Schneider et al., 2022; Favaro et al., 2023). Leveraging this quantitative EEG analysis and its established reference values in a pediatric population, we investigated where the electrophysiological state of the isolated hemisphere falls along the spectrum between states associated with vivid awareness and those largely characterized by reduced consciousness.*

We then operationalized the hypothesis in the **Methods** section, for clarity and specificity:

*We estimated narrow-band and broad-band EEG slowing, by means of Slow-Delta power and of the spectral exponent, before and after surgical disconnection, in both the disconnected and in the homologous contralateral cortex. We hypothesized that the disconnected cortex would display a larger degree of narrow- and broad-band slowing with respect to the contralateral cortex, and to its state before surgery, leading to larger spatial asymmetry after surgery.*

We also acknowledge that further measures of consciousness may have been employed. In fact, in recent years, numerous EEG-based markers of consciousness have been proposed. In selecting the most appropriate ones for our population, we carefully considered both methodological and clinical constraints. First, markers derived from Event-Related Potentials (ERPs), such as mismatch negativity or P300, are not applicable in our context, as sensory stimuli are unlikely to reach the isolated cortex in the post-surgical phase due to thalamocortical disconnection. An alternative approach would involve direct perturbation of the cortex, with techniques like Transcranial Magnetic Stimulation (TMS) combined with EEG, which allow the estimation of the capacity for consciousness through the Perturbational Complexity Index (Casali et al., 2013; Comolatti et al., 2019). However, despite recent advancements in TMS-EEG applications in epilepsy (Rossi et al., 2021; Pang et al., 2022), this approach raises significant safety concerns in pediatric populations, especially in patients with severe epileptic encephalopathies. Critically, the retrospective design of our study precluded the acquisition of perturbational or task-based data.

We therefore focused on features extractable from spontaneous EEG recordings collected during standard clinical evaluations, with a low number of electrodes. This led us to exclude connectivity-based metrics (e.g., Bourdillon et al., 2020) which require high-density EEG to be reliable (Asayesh et al., 2024). Further, connectivity measures are sensitive to volume conduction and would require an adequate reference choice and an appropriate reconstruction of cortical sources, potentially leading to spurious inter-hemispheric connectivity in the absence of any structural connectivity. Concerning other metrics, including complexity measures (see Sarasso et al., 2021 for a review), they would require careful adaptations to minimize the influence of epileptogenic segments and muscular noise, and, most crucially, their use would require first careful investigation in pediatric cohorts to provide a meaningful reference for interpretation.

Given the already substantial length of the main text, we opted to condense the above discussion and direct the reader to the relevant references. We nonetheless acknowledge the possibility that alternative markers may better characterize the isolated cortex, and we now explicitly address this in the '**Limitations and Future Directions**' section as follows:"

*Finally, additional EEG-based markers of consciousness could be employed (Sitt et al., 2014; Sarasso et al., 2021; Maschke et al., 2023b; Sevenius Nilsen et al., 2020) and assessed against an appropriate normative reference, to further characterize the state of the isolated cortex.*

**10)** In particular, the introduction suffers from a lack of clear statements of aim/hypotheses. Given this, it is unclear what were the pre-planned analyses, and which were descriptive/post-hoc. For example, the claim "We accordingly quantified slow wave activity by narrow band spectral analysis within the SlowDelta band (0.5-2 Hz)," indicates that it was preplanned, but i get the feeling (from looking at the figures) that this range is rather post-selected since it is the only region with significant differences. Please specify and make explicit what the concrete hypotheses were (prior to looking at the data).

**>>** We appreciate the feedback regarding the narrow band spectral analysis in the previous version of the manuscript and recognize that it could benefit from a better introduction.

For the narrow-band analysis, we chose to target Slow Wave Activity in the spectral domain, given that this activity is the typical result of structural or functional deafferentation of the cortex. Several studies showed that the deafferentation of cortical tissue, such as in isolated cortical gyri (i.e., cortical slabs) (Timofeev et al., 2000) and cortical slices in vitro (Capone et al., 2019), results in the emergence of slow waves, which end up dominating the time series and show the largest power across frequencies (Sanchez-Vives et al., 2017). These studies were properly mentioned by the reviewer in the above **comment 6**.

Moreover, other conditions such as NREM sleep (Steriade, Nunez, & Amzica, 1993; Achermann and Borbély 1997; Csercsa et al., 2010; Molle and Born, 2011; Staresina et al., 2015; Bersagliere et al., 2018), and general anesthesia (Nì Mhuircheartaigh et al., 2013; Warnaby et al., 2017), result in the emergence of slow waves in the cortex with a frequency of ~1 Hz.
We selected the 0.5-2 Hz range, since slow waves are usually visually detected employing a corresponding period of 0.5 to 2 seconds, according to the AASM manual for sleep scoring (Iber et al., 2007; Troester et al., 2023).

We now clarify the **Methods** accordingly:

*Since slow wave activity has a frequency of ~1 Hz both in sleep [Steriade, Nunez, & Amzica, 1993; Achermann and Borbély 1997; Csercsa et al., 2010; Molle and Born, 2011; Staresina et al., 2015; Bersagliere et al., 2018] and in general anesthesia [Nì Mhuircheartaigh et al., 2013; Warnaby et al., 2017] and is described as EEG oscillations with a period between 0.5 -2 seconds by the AASM manual for sleep scoring (Iber et al., 2007; Troester et al., 2023], we accordingly estimated Slow Delta power as the mean absolute PSD between 0.5 and 2 Hz, subsequently transformed by log10 (results for absolute total power are shown in* ***Supplementary Figure 2 )****.*

However, we would like to stress that the effect at hand is not a result of a post-hoc fiddling with the data, but something really macroscopic and robust. In fact, not only the effect can be seen with the naked eye on the raw non-preprocessed recordings (see one instance below), but **such effect can be seen** in the overall delta band 0.5-4 Hz (Group-level statistics: asymmetry after surgery T(9)= -3.287, p = 0.004; disconnected cortex change pre-post T(9)=-2.81, p <0.02), and even **irrespectively of the frequency band,** by considering the total power (i.e. summing the power spectral density across all frequencies, which we report below and in a new **Supplementary Figure 2**) in a new **supplementary section** (**S2**)

An example screenshot of an EEG trace, obtained before the preprocessing steps, recorded 7 months after left hemispherotomy in a 9-year-old girl with drug-resistant epilepsy of structural etiology, due to the sequelae of a perinatal stroke involving the left middle cerebral artery. X-axis ticks are placed every 2 seconds. The tracing clearly reveals a prominent slowing over the isolated left hemisphere. To clarify this effect we added a new section to the Supplementary Material:

***S2 The isolated cortex shows an increase in total power, in the context of a broad-band power redistribution***

*While the results in the patient dataset for the narrow-band analysis of the PSD (in the Slow Delta band) are presented in the main text, we here report the results on total absolute power (i.e., the sum of the power spectral density across all frequencies). Regarding spatial inter-hemispheric contrasts (contralateral vs. disconnected hemisphere), before surgery there was no significant difference in total power between the disconnected cortex and the contralateral cortex (T(9) = 0.828, p = 0.4293, Supplementary Figure 2, left panel). After surgery, a significant difference in total power emerged between the disconnected cortex and the contralateral cortex (T(9) = –2.917, p = 0.0171, Supplementary Figure 2, middle panel). Regarding temporal contrasts (Difference Post-Pre surgery), total power increased post-surgery relative to pre-surgery within the disconnected cortex (T(9) = 2.577, p = 0.0298), but not within the contralateral cortex (T(9) = 0.541, p = 0.6019), , Supplementary Figure 2, right panel.*

***Supplementary Figure 2. Surgical disconnection increases overall total EEG power in the isolated hemisphere with respect to the contralateral hemisphere after surgery and to the state of the isolated cortex prior surgery.*** *Total absolute EEG power in the disconnected and contralateral cortex before and after surgery. Boxplots show total power values for the disconnected cortex (light red: pre-surgery; red: post-surgery) and the contralateral cortex (light blue: pre-surgery; blue: post-surgery).*

*Left panel: Before surgery, total power did not differ significantly between the two hemispheres. Middle panel: After surgery, total power was significantly higher in the disconnected cortex compared to the contralateral side. Right panel: Change in total power from pre- to post-surgery. A significant increase was observed in the disconnected cortex (red boxplot), but not in the contralateral cortex (blue boxplot). Patients who underwent complete hemispherotomy were marked with a dot, while those who underwent temporo-parieto-occipital disconnection were marked with a square.*

*The disconnected cortex showed a robust increase in power, primarily due to low-frequency activity; this effect remained statistically significant even when considering total absolute power across all frequencies (see* ***Supplementary Figure 2)****. This increase in power was most pronounced in the Slow Delta band (0.5–2 Hz), as shown in* ***Figures 1 and 2****. However, the rise in Slow Delta power was not only an isolated narrow-band effect; rather, it occurred in the context of a broad-band spectral redistribution. The slope of the PSD increased, as well as its offset (****Figure 2****, inset of panel A), according to an overall clockwise rotation of the PSD profile. Specifically, while the spectral offset can change independently of the slope (reflecting a vertical shift in the PSD), any change in the spectral exponent necessarily leads to a change in offset when the PSD rotates around a pivot point at a non-zero frequency     (for a visual explanation see:     https://github.com/fooof-tools/Visualizers#spectral-rotation; see also* ***section S3****, and Favaro et al., 2023, for a discussion). This rotation reflects a global steepening of the PSD and a redistribution of power from higher to lower frequencies, quantified by a more negative spectral exponent (****Figure 2D****). Notably, changes in the spectral exponent showed stronger statistical evidence than changes in Slow Delta power (see* ***Supplementary Figure 6****). These joint spectral changes point to a single underlying transformation in cortical dynamics.*

Note, spectral redistribution is also discussed in the answer to the related **comment 4**.

**Methods**

- **11)** You say "We retrospectively selected our participants". This, along with the inclusion criteria, indicates that all recordings were already done, but it is not obvious (as you also say "Ten participants were recruited"). Please be a bit more specific about this. I need to be spoon fed :)

>> We thank the reviewer for the comment, as it gives us the opportunity to clarify the methods.

We retrospectively searched for recordings of patients referred to the Munari Center for Epilepsy Surgery of Niguarda Hospital in Milan that met several inclusion/exclusion criteria. We specify accordingly the sentence:	*~~Ten participants were recruited~~ The recordings from ten patients were selected on the basis of the following inclusion criteria (...)*

- **12)** You say "which required relatively high spatial specificity". What do you mean by this? Please clarify.
  >> We appreciate the reviewer’s input, which gave us the opportunity to clarify how we ensured compatibility of the EEG montage across datasets. To analyze the EEG of hemispherotomy patients, we did not use an average reference as in Favaro et al., 2023–which would smear large voltage deflections across the scalp–but rather we used bipolar derivations between all pairs of nearest neighboring electrodes, excluding the midline (hence the phrasing ‘which required relatively high spatial specificity’). Accordingly, we re-referenced the EEG data of the pediatric reference dataset as we did for the patients dataset. We make explicit the location of such bipolar derivations in the **Methods**, where spatial specificity was first considered.

*Finally, to observe lateralized effects with high spatial specificity under our low-density EEG montage, bipolar derivations between all pairs of nearest neighboring electrodes (excluding the midline, Left hemisphere: {'Fp1-F3'  'F3-C3'  'C3-P3'  'P3-O1'  'F7-T3'  'T3-T5'  'F7-F3'  'T3-C3'  'T5-P3'  'Fp1-F7'  'F7-C3'  'F3-T3'  'T3-P3'  'C3-T5'  'T5-O1'}, Right hemisphere: {'Fp2-F4'  'F4-C4'  'C4-P4'  'P4-O2'  'F8-T4'  'T4-T6'  'F8-F4'  'T4-C4'  'T6-P4'  'Fp2-F8'  'F8-C4'  'F4-T4'  'T4-P4'  'C4-T6'  'T6-O2'} ) were considered for subsequent analyses.*

The specific sentence mentioned by the reviewer, has been now rephrased for better clarity, explicitly mentioning to the bipolar montage:

*Furthermore, we digitally re-referenced the EEG recordings from the pediatric reference dataset to match the bipolar montage used in the patient dataset, ensuring methodological consistency for comparative analyses (see section* ***Data Acquisition and Preprocessing****).
~~Finally we adapted the virtual EEG reference of the pediatric reference dataset to that of the patient dataset, which required relatively high spatial specificity.~~*

- **13)** There is a notable change in the narrative structure of the methods in the "Sigma periodic activity" section. That is, there is much more background and justification for the analysis than in the rest of the methods. In particular, given that there is little reason to believe there will be spindle activity in the disconnected cortex (arent spindles largely driven by thalamic activity?), it seems rather lopsided. Please reconsider whether this analysis deserves so much space. You also go on to repeat much of the justification and explanation in the results section.

>> We thank the reviewer for this valuable feedback. Our analysis was confirmatory in nature; we did not expect to observe spindles, but rather we aimed to demonstrate that their absence represents a significant and notable distinction from NREM sleep. After thoughtfully considering the reviewer’s comment, we agree that this analysis is not central to the main findings of the paper; thus, we decided to follow the reviewer’s advice and moved the section to the **Supplementary Material.** In doing so, we have condensed the lengthy sections in the main text. Specifically, we now state in the main methods section:

***Sigma oscillations and spindle activity***
*Given the established thalamic origin of sleep spindles, we confirmed their absence in the disconnected cortex through multiple assessments: visual inspection by a trained neurologist, analysis of total and periodic power in the sigma band, and automated detection of sigma-band bursts (****Supplementary Material, section S14).’***

In the **Result** section:

*‘Overall, the disconnected cortex in awake patients showed a remarkable emergence of Slow Delta activity, leading to a state departing from wakefulness, yet different from full-fledged NREM sleep. Specifically, the disconnected cortex lacked spindle activity (****Supplementary Material, section S14****) and showed slow waves with reduced amplitude and longer period compared to NREM sleep, coherent with the absence of thalamic synchronizing input.*

In the **Discussion** section:

*As expected under conditions of thalamic disconnection, post-surgery slow waves were not accompanied by spindle activity (according to visual inspection, sigma burst detection, and spectral analysis of total and periodic power,* ***Supplementary Material, section S14)***

**Results**

- **14)** The large range of ages in the sample is a bit worrisome, given that there are large changes in EEG patterns that happen in this age range (see for example the review by Gorgoni et al (2020) in Sleep Medicine). Consider providing some supplementary figures showing how the key markers used here (and differences in them) are influenced by age.

**>>** We thank the reviewer for this important observation. In response, we conducted additional age-matched analyses to verify that our main findings were not confounded by developmental differences in EEG. Specifically, we sought to confirm that the electrophysiological profile of the disconnected cortex resembled deep NREM sleep (main **Figure 3**), beyond any potential effects of age. We thus applied a pairwise age-matching approach (illustrated in **Supplementary Figure 10**) to compare spectral exponent values of the patient dataset to those of the pediatric reference dataset across wakefulness and NREM sleep stages (**Supplementary Figure 11**). This analysis confirmed that the spectral exponent values of the disconnected cortex resembled N1 sleep before surgery and shifted toward values characteristic of deeper NREM stages (N2–N3) after surgery, independently of age.

Moreover, we extended the same age-matching approach to the analysis of the amplitude and period of slow-delta oscillations, comparing the disconnected cortex to pediatric reference values during wakefulness and NREM sleep (**Supplementary Figure 15**). This analysis replicated the core results reported in **Figure 4**, showing that the disconnected cortex exhibited amplitude values intermediate between wakefulness and NREM sleep (with larger differences observed relative to wakefulness), and significantly longer oscillation period than either condition, independently of age.
Together, these results strengthen the age-independent validity of our findings. Below we report the two new sections (**S9 and S12**) and the three new **Supplementary Figures** **(10, 15  11)** in the Supplementary material dedicated to age-matched comparison.

***S9 - Supporting age-matched comparison of spectral exponent between Patient and Reference datasets***

*Given the well-known impact of age on EEG features (Kurth et al., 2010; Gorgoni et al., 2020; Favaro et al., 2023), we conducted supplementary analyses to factor out the effect of age in our findings related to the comparison between the patient and the pediatric reference dataset. Primarily, we sought to verify if the electrophysiological profile of the disconnected cortex resembled deep NREM sleep (main* ***Fig. 3****), beyond any potential effects of age. Accordingly, we applied a pairwise age-matching approach (illustrated in* ***Supplementary Figure 10****) to compare the spectral exponent of the patient dataset across conditions (pre-post, disconnected-contralateral) to those of the pediatric reference dataset across wakefulness and NREM sleep stages (****Supplementary Figure 11)****. Moreover we applied the same age-matched approach to compare slow wave properties of the disconnected cortex of the patient dataset to those of the pediatric reference dataset in wakefulness and deep NREM sleep (N2 - N3) (****Supplementary Figure 15)****.*

**Supplementary Figure 10. *Matching subjects by age across the Patient and the Reference dataset.*** *The figure shows age-matching across the patient dataset and a subset of the reference dataset; with each subplot comparing the age of patients to that of matched control subjects from the pediatric reference dataset, separately for pre- and post-surgery. The x-axis represents subject number (sorted by age), and the y-axis shows age in years. In both panels (Pre-Surgery and Post-Surgery), each red dot (patient) is paired with a nearby blue dot (reference subject), indicating that age-matching was performed on an individual basis. The dashed lines connecting each pair indicate pairwise matching, and the vertical distance shows the age difference between each pair. All patients were age-matched within approximately 1 year or less, which supports a robust age-controlled comparison. Such tight age matching was meant to ensure that any differences observed between patients and the whole reference data (e.g., in spectral slope or slow-wave activity) would not be driven by age-related changes.*

*Once age-matched subjects were identified, we compared the spectral exponent values between the patients dataset (in the disconnected or contralateral cortex, before or after surgery) and the the subsample of the pediatric reference dataset (either during wakefulness, N1, N2, or N3 sleep) (****Supplementary Figure 11****), by computing the distribution of pairwise differences between age-matched subjects. We then identified the vigilance states showing the greatest similarity through a bootstrap analysis of the mean pairwise differences across conditions (violin plots in* ***Supplementary Figure 11*** ***B,D,F,H)****.*

*We first qualitatively assessed how many hemispherotomy cases showed a steeper spectral decay than their age-matched counterparts during deep NREM sleep in the neurotypical reference dataset. Thus, we considered the proportion of age-matched cases in the patient dataset that showed a negative difference relative to N2 and N3 sleep of the pediatric reference dataset, indexing a steeper spectral decay in patients. The contralateral cortex did not exhibit a steeper spectral decay relative to either N2 or N3 sleep in any case (****Supplementary Figure 11 F,H****). Before surgery, the pathological cortex showed a steeper spectral decay—indexed by negative differences—relative to N2 sleep in 2/10 cases and to N3 sleep in 0/10 cases (****Supplementary Figure 11 B****). After surgery, the disconnected cortex showed a steeper spectral decay relative to N2 sleep in 9/10 cases and to N3 sleep in 3/10 cases (****Supplementary Figure 11 D****).*

*For the contralateral cortex, before and after surgery alike, the distribution of pairwise differences was centered between wakefulness and N1 sleep values; the bootstrap distribution of mean pairwise differences indicated larger similarity with wakefulness and N1 sleep (****Supplementary Figure 11, F*** *and* ***H****), revealing a mild slowing (as qualitatively noted in previous literature by Smith et al., 1991) but still distinct from deeper NREM stages.*

*For the pathological cortex before surgery, the distribution of pairwise differences was centered tightly around zero relative to N1 sleep values; the bootstrap distribution of mean pairwise differences indicated the greatest similarity to light (N1) sleep (****Supplementary Figure 11  B****).*

*For the disconnected cortex after surgery, the distribution of pairwise differences was centered between N2 and N3 sleep values; the bootstrap distribution of mean pairwise differences indicated larger similarity with deep (N2-N3) sleep (****Supplementary Figure 11 D****).*

***Supplementary Figure 11. Age-matched analysis comparing spectral exponent values in hemispherotomy to the pediatric reference dataset across wakefulness and NREM vigilance stages.*** *Each column presents two plots corresponding to a condition of the Patient dataset (Left to Right: Disconnected cortex pre- and post-surgery, followed by Contralateral cortex pre- and post-surgery), compared to each vigilance stage of the pediatric Reference dataset (wakefulness, N1, N2, N3, colored in shades spanning from green to blue). The first row* ***(A,C,E,G)*** *shows the absolute spectral exponent values, with lines connecting age-matched subjects across the Patient and Reference datasets. The second row* ***(B,D,F,H)*** *displays pairwise differences in spectral exponent: each dot represents the difference for a subject pair, calculated by subtracting the value of a subject in a given vigilance stage of the Reference dataset from that of the corresponding subject in the Patient dataset. Violin plots depict the bootstrap distribution of mean pairwise differences across subjects, thereby providing an estimate of the expected mean distance between paired conditions.
Overall, age-matched results revealed a mild slowing in the contralateral cortex, both pre-surgery (****E,F****) and post-surgery (****G,H****), with spectral exponent values intermediate between wakefulness (W) and N1. Importantly, age-matched results replicated the main findings (Fig. 3): spectral exponent values in the Disconnected cortex pre-surgery closely aligned with those observed during N1 sleep (****A,B****), while post-surgery values were intermediate between N2 and N3 sleep* ***(C,D)****.*

These supplementary results are briefly mentioned in the **results** section of the main text as:

*Age-matched analysis further strengthened this result, revealing that the disconnected cortex showed lower spectral exponent values in all 10 cases compared to their reference counterparts during both wakefulness and N1 (****Supplementary Material S9, Supplementary Figure 11****).  (...)*

*Supplementary age-matching analysis confirmed these findings (****Supplementary Material S9****,* ***Supplementary Figure 11*** *); regardless of age, while the spectral exponent values in the disconnected cortex resembled those of N1 sleep before surgery, they shifted toward values characteristic of deeper NREM stages (N2–N3) after surgery.*

Accordingly, in the **discussion** section, we now mention:

*Age-matched analysis further supported this finding, revealing lower spectral exponent values in all 10 cases compared to their reference counterparts during both wakefulness and N1 sleep (****Supplementary Figure 11****)*

We have included a new supplementary section to address potential age-related biases in the comparison of slow-wave properties with the reference dataset:

***S12 - Supporting age-matched comparison of slow wave properties between Patient and Reference datasets***
*To further validate the analysis on slow wave properties of the disconnected cortex, we adopted the same age-matching approach detailed above (****Supplementary Figure 10****) and applied it to the analysis presented in* ***Fig 4*** *(Mean amplitude of the slow-delta envelope and period of slow-delta oscillations in the disconnected hemisphere, compared with respect to wakefulness and NREM sleep in the reference pediatric sample). This analysis corroborated the main results on slow wave properties of the disconnected cortex in relation to pediatric reference values, by controlling for the effects of age.*

*The amplitude of the slow-delta envelope of the disconnected cortex showed intermediate values between those of wakefulness and NREM (N2 and N3 jointly considered,* ***Supplementary Figure 15A****, consistently with the main results in* ***Fig 4A****); through age-matching its values resulted significantly larger than those of wakefulness ( T(9)=3.741, p = 0.0046, with 9/10 subjects showing a consistent pairwise difference) and not significantly different from those of NREM sleep (T(9)=-1.633, p = 0.13, with 8/10 subjects showing a consistent pairwise difference,* ***Supplementary Figure 15B)****.*

*Further, the period of slow-delta oscillations of the disconnected cortex showed values higher than those of both wakefulness and NREM* ***(Supplementary Figure 15C****, consistently with the main results in Fig 4 B), through age-matching its values resulted significantly larger than those of both wakefulness (T(9)=4.783, p = 0.001, with 10/10 showing a consistent pairwise difference) and NREM sleep (T(9)= 3.686, p = 0.005, with 10/10 showing a consistent pairwise difference,* ***Supplementary Figure 15D****).*

*This analysis replicated the core results concerning the properties of Slow Delta activity reported in* ***Figure 4****: the disconnected cortex showed amplitude values intermediate between wakefulness and NREM sleep–with larger differences observed relative to wakefulness than to sleep–and significantly longer oscillation period than either condition, independently of age.
Together, these results strengthen the age-independent validity of our findings.*

 **Supplementary Figure 15. *Age-matched analysis of amplitude and period of slow-delta oscillations in the disconnected cortex compared to the reference pediatric dataset during wakefulness and NREM sleep.*** *Results are drawn from the disconnected cortex in all 10 hemispherotomy patients age-matched to a subset of subjects from the reference dataset. (****A–B****) Slow-delta envelope amplitude comparison across datasets. (****A****) Boxplots show the amplitude of the slow-delta envelope in the disconnected cortex compared to wakefulness and NREM sleep in the reference dataset, with lines connecting age-matched subjects across datasets.(****B****) Pairwise differences after age-matching, relative to wakefulness and NREM sleep.* *(****C–D****) Mean period of slow-delta oscillations comparison across datasets. (****C****) Boxplots show the mean period in the disconnected cortex compared to the reference dataset, with lines connecting age-matched subjects across datasets. (****D****) Pairwise differences after age-matching.*

*Overall, the disconnected cortex exhibited slow-delta amplitude values intermediate between wakefulness and NREM sleep, and period values significantly longer than both wakefulness and NREM sleep, consistent with the findings reported in* ***Figure 4***.

These findings are reported in the main **results** as follows:

*Supplementary age-matched analysis confirmed the key findings on the properties of slow-delta activity (****Supplementary section S12, Supplementary Figure 15****): regardless of age, in the disconnected cortex, amplitude values were intermediate between those observed during wakefulness and NREM sleep—with more pronounced differences relative to wakefulness—and the oscillation period was considerably longer than in either state.*

- **15)** Is the 11 year old girl really "A representative example?" Especially given that it is one of the older patients?

**>>** We acknowledge the reviewer’s concern. The subject was selected as a representative example based on the alignment of her spectral features with the group-level pattern, rather than on age, which is not a primary variable of interest in our analysis (and is now accounted for in a new supplementary analysis). As shown in **Figure 1**, the shape of the PSD from the pathological cortex, both before and after surgery, closely mirrors the grand-average presented in **Figure 2**, reflecting the typical spectral changes observed across the cohort.
Moreover, for the benefit of the reviewer, we show below the PSD from a younger subject than that of **Figure 1,** displaying similar spectral changes to the other representative subject.

**A display of the PSD and EEG features of a young representative subject.** This child underwent left hemispherotomy at the age of 5 due to drug-resistant epilepsy of structural etiology (post-stroke sequelae consisting of fronto-insular and perirolandic porencephaly on the left side). The age at EEG recordings was 5 years pre-surgery and 7 years post-surgery. Left panel: the EEG PSD, averaged by geometric mean across intrahemispheric bipolar derivations, reveal an increase of slow frequency activity (< 2 Hz) and a broad-band steepening of the PSD over the disconnected hemisphere post-surgery, which is evidenced in the inset graph by the the linear fit of the PSD under double logarithmic axes (power-law fit). The shaded area indicates the bootstrapped 95% confidence interval for the geometric mean across electrodes. Right Panel: for all the neighboring intrahemispheric bipolar derivations, we display the values of Slow-Delta power (0.5-2 Hz), and of the spectral exponent, indexing the slope of the PSD decay (0.5-20 Hz). 

- **16)** I am a bit confused as to why fig 2 is not given more space in the results section, as it seems to be the main figure/result pertinent to the central thesis of the paper.

>> We thank the reviewer for highlighting the central importance of **Figure 2**. We agree that this figure plays a key role in illustrating the core findings of the study, and we would like to clarify that it is already extensively described and integrated within the Results section, in the first version of the manuscript**.** We first mention **figure 2** at the end of the result section **Surgical disconnection and EEG slowing.**

*As shown in* ***Figure 2****, at the population level the disconnected cortex following surgery displayed increased Slow-Delta power and an overall PSD redistribution towards low frequencies and a steeper broad-band decay, as evident from the PSD and its 1/f-like fit, aggregated across patients.*

Further, we make extensive reference to **Figure 2** in the result section **Asymmetry of cortical states after disconnection.**

*Contrasting the pre-post difference between cortices (...) (* ***Figure 2 C, Figure 2 E****)*

*When assessing spatial inter-hemispheric contrasts (contralateral vs disconnected cortex), (...) (****Figure 2B, 2D****) , Assessing temporal contrasts (...) (Post-Pre surgery ,* ***Figure 2C, 2E****)*

We do not report the section in its entirety here, given its length; for each relevant contrast of each EEG feature, the results depicted in **Fig. 2** are described in terms of a T statistic, its p value, and a description of how many patients followed the described pattern. For instance, both the previous and current version of the article mention:

*the spectral exponent was significantly more negative in the disconnected cortex than in the contralateral, both pre-surgery (T=2.395, p=0.0402; more negative in 7/10 patients) and, especially, post-surgery (T=7.118, p<0.0001, more negative in  10/10 patients,* ***Figure 2D****).*

**Figures**

- **17)** Fig 1 and 2. The slopes may be significantly different, but the differences in the PSD are vastly smaller than those seen the original paper by Colombo et al. proposing the spectral slope as a measure of consciousness. E.g. the only significant difference in absolute powers is in the low delta range. This could be explained by the difference in age/recording equipment etc, but to me the rather minor changes in the actual PSD make me distrust strong claims about what we can say about the awareness of the underlying hemisphere. Just eye-balling it, the changes in the 1-20 hz seen in this study resemble more the changes previously reported to the ketamine state (see this from colombo et al. although, of course, I understand that "eye-balling" is insufficient :)).

>> We thank the reviewer for this thoughtful observation. While visual inspection of the grand-average PSD may suggest modest changes in absolute values, this impression is misleading—on a log scale, an average change in Slow-Delta Power from ~1.5 to ~2.5 represents a change of nearly an order of magnitude, indicating a substantial increase in low-frequency power. Further, we caution against relying solely on this approach, as it can obscure key relative changes—particularly in broadband spectral trends—due to substantial inter-individual variability in absolute power (see again Donoghue et al., 2022).

This is precisely why we included both narrow-band (Slow-Delta power) and broad-band (spectral exponent) measures: the spectral exponent captures global shifts in power distribution across frequencies, providing a more robust and interpretable metric of EEG slowing, especially in heterogeneous clinical populations. Indeed, the spectral exponent showed pronounced, robust changes (spatial asymmetry and lateralized change post-surgery was observed in all patients), which had stronger statistical evidence than Slow-Delta Power, and can provide a more meaningful ground for comparisons.

As for the comparison with ketamine-induced states, several distinctions are important:

First, due to the influence of neuronal mass and subcortical inputs on absolute power, Slow-Delta values in the isolated cortex are not directly comparable to those in anesthetized adults. The isolated hemisphere lacks interhemispheric connectivity and subcortical synchronizing input, leading to a ceiling in possible voltage excursion (see Discussion of the main text, paragraph **Properties of post-disconnection slow waves**). In this context, relative measures such as the spectral exponent provide a more meaningful basis for comparison.

Second, the spectral exponent in the 1–20 Hz range was considerably more negative in the disconnected cortex (mean: −2.22, SD: 0.18) than under ketamine anesthesia (mean: −1.40, SD: 0.43; Table 1 in Colombo et al., 2019). This reflects a much steeper spectral decay and a more pronounced clockwise rotation of the PSD, indicating overall slower dynamics—distinct from the ketamine pattern.

Third, a defining hallmark of the dissociative state induced by ketamine is the flattening of the high-frequency PSD slope, often described in the literature as ketamine-induced beta-gamma activity. This feature was entirely absent in our data. While our primary analyses focused on frequencies below 20 Hz to minimize EMG contamination in pediatric EEG (which could bias the slope towards flatter values), we nonetheless report for the reviewer’s benefit the spectral decay of the 20–40 Hz range. The 20-40 Hz spectral exponent in the isolated cortex remained clearly negative (mean: −1.40, SD: 0.93), in stark contrast to the near-flat slope reported under ketamine (mean: −0.08, SD: 0.50, Table 1 in Colombo et al., 2019). We also display the grand-average PSD across the full 0.5–40 Hz range in a new **Supplementary Figure 4,** specifically to illustrate that the spectral decay continues in the high-frequency range, revealing the absence of the ketamine high-frequency signature.

***Supplementary Figure 4.*** ***Group-level broad-band PSD and aperiodic trend in hemispherotomy, estimated over a broader 0.5-40 Hz range.*** *The geometric mean of the PSD was taken first across electrodes, and subsequently across patients, for the observed PSD (main graph) as well as for the aperiodic fit of the PSD estimated in the 0.5-40 Hz range (corresponding to a straight line under logarithmic scale for both x and y axes, inset graph). The shaded area indicates the bootstrapped 95% confidence interval for the geometric mean across participants. This graph illustrates a broader frequency range to contextualize Figure 2A and shows that the broad-band spectral decay continues in the higher frequency range, above 20 Hz, in all conditions.*

In sum, whereas ketamine shows only a mild steepening of the spectral decay in the 1–20 Hz range and a characteristic flat spectrum in the 20–40 Hz band—reflecting increased beta–gamma activity—the disconnected cortex exhibited a markedly steeper spectral decay in the analyzed 1–20 Hz range, and its spectrum continued to decay in the 20–40 Hz high-frequency region. This pattern clearly diverges from the spectral profile characteristic of the dissociated-anesthetized state induced by ketamine.

- **18)**  Figure2: Why is the statistical comparison swapped here, relative to fig 1? Why is not the pre vs post different directly shown in the figure? I recommend consistency

>> We thank the reviewer for this comment and the opportunity to clarify our graphical choices. First, we note that **Figure 1** does not include statistical comparisons, as it presents data from a single representative subject, to provide an intuitive overview of the key electrophysiological patterns. In this context, the pre- vs post-surgery values for the disconnected hemisphere are shown directly (**Fig. 1D**), along with values from the contralateral hemisphere for visual comparison.

In contrast, **Figure 2** presents group-level data, where the layout reflects the structure of the study (pre-post design). Specifically, panels B and D show the values for each hemisphere in the pre- and post-surgical conditions (first and second columns), while panels C and E explicitly display the computed pre-post differences.
In sum, these layouts were chosen to provide clarity and immediacy for the representative data, and to ensure completeness, without sacrificing interpretability, for group-level data.

- **19)** Fig 5: I recommend this be "demoted" to supplements, preferably together with the majority of the spindle discussion (as it seems more confirmatory of surgery success than anything else—im happy to be corrected on this point!)

>> We agree that the spindles analysis is confirmatory in nature, in fact we did not expect any spindles in the isolated cortex, given the thalamic disconnection. This analysis was meant to show the limits of the comparison with sleep. According to the reviewer’s suggestion, we moved the section to the Supplementary material entirely. Please also see the answer to **comment 13.**

**Discussion**

- **20)** The first sentence in the discussion is not correct. There are many studies (the earliest I found from a quick google search was from 1950) investigating how EEG changes following hemispherectomy in humans. Please edit to make the novel aspects of this study correctly reflected. The easiest would (probably) be to emphasize the context of consciousness studies. Or just remove it—no need for salesmanship.

>> Following the reviewer's observations, we clarified in the introduction the difference between hemispherectomy and hemispherotomy, and we contextualized our study within the existing literature on epileptic activity. We highlight that no previous study has focused on the background EEG of the isolated cortex in awake human patients following hemispherotomy.
To avoid redundancy with the subsequent text, we have removed a portion of the opening paragraph of the Discussion. This revision also satisfies the reviewer’s request for deletion. See also **comment 3.**

- **21)** Also in the first paragraph of the discussion, i think the following statement needs revision: "Such broad-band neurophysiological slowing, indexed by the spectral exponent, was similar to that previously found in the physiological [...], pharmacological [...] and pathological unconscious states." In particular, I think it is questionable to what extent changes seen here were "similar" (although the direction of change was the same) and i would be hesitant to calling them "unconscious states", as these states are all riddled with (albeit arguably "reduced" and disconnected) conscious experiences.

**>>** We thank the reviewer for this thoughtful comment, which led us to revise the original sentence to address the terminology. We acknowledge the concern regarding the use of the term “unconscious states”, given the growing evidence that some residual forms of experiences—albeit often fragmented, incoherent or atypical—may occur even in conditions traditionally labeled as unconscious. In line with changes made elsewhere in the manuscript, we now avoid this binary framing, instead referring to states where consciousness is absent, or less likely to occur, and reduced or degraded.

In this revision, the whole incipit of the discussion containing the quoted text, has been removed, for the sake of brevity and to avoid redundancy with the rest of the discussion. See also **comment 27.**

- **22)** Without also noting that the "pre surgery state" in the disconnected hemisphere resembled N1 sleep, I feel the following is over-selling: "Upon direct comparison with a reference pediatric sample, the disconnected cortex showed a broad-band spectral exponent resembling deep NREM sleep."

>> The reviewer is correct in noting that the pathological cortex exhibited some degree of slowing resembling N1 sleep prior to surgery. We have described this finding in its entirety in the **Results** section “**The awake spectral exponent of the disconnected cortex aligns with values of physiological NREM sleep”**; including the supporting new evidence stemming from age-matched analysis.

*Moreover, the disconnected cortex before surgery showed values similar to the N1 sleep distribution; crucially, after surgery the disconnected cortex showed values distinct from the wakefulness distribution and intermediate between those of the N2 and N3 sleep distributions.*

*Supplementary age-matching analysis confirmed these findings* ***(Supplementary Material S9, Supplementary Figure 11)****; regardless of age, while the spectral exponent values in the disconnected cortex resembled those of N1 sleep before surgery, they shifted toward values characteristic of deeper NREM stages (N2–N3) after surgery*

The section where the quoted text appeared (the incipit of the original discussion) has now been entirely removed for the sake of brevity and avoiding redundancy.
To properly address the findings of the pathological cortex before surgery in detail, we have dedicated a paragraph within the **Limitations and Future Directions** section (already in the first version of the submitted article), where we discuss possible interpretations of pre-surgical slowing—namely, gliosis, malformation, and interictal dynamics. For transparency, we report the relevant excerpt below.

*Even before surgery, the pathological cortex exhibited slightly lower mean spectral exponent values compared to its contralateral counterpart, with a distribution centered between those of wakefulness and N1 sleep in the reference pediatric sample (****Figure 3****). This relative slowing under baseline conditions may be partially influenced by interictal epileptiform discharges, although these pathological grapho-elements were meticulously excluded during data pre-processing by a trained neurologist (see* ***Supplementary Figure 1****). Additional contributing factors could include focal cortical malformations and gliosis (Panet-Raymond and Gotman, 1990; Vanrumste et al., 2005; Blümcke et al., 2011). Alternatively, some degree of slowing may be contributed by local slow waves that play a protective role against aberrant epileptiform activity (Sheybani et al., 2023). In this context, the role of the pathological hemisphere and its contribution to consciousness prior to surgery remain open questions.*

- **23)** I also, again, fail to see the relevance (or need for emphasis) of the following sentence, given that the hemisphere is disconnected from subcortical structures (or am I misunderstanding?). If anything, this seems to me like a basic confirmation that the surgery was successful, but I fail to see the reason for why it requires so much space throughout.
  >> We appreciate the reviewer’s comment and the opportunity to clarify the rationale behind this emphasis. The narrow-band and broad-band slowing we observed was largely confined to the disconnected cortex. This pattern is therefore more plausibly attributed to the loss of subcortical (thalamic) inputs, rather than to loss of interhemispheric cortico-cortical inputs alone—especially given that the contralateral cortex, which also undergoes a disconnection of interhemispheric inputs, does not show a comparable slowing (in this sense, the contralateral cortex may be compared to a hemisphere in a split-brain).
  While this finding may not be surprising in itself, it offers a valuable opportunity to clarify the mechanistic specificity of the slowing, distinguishing the role of subcortical versus cortical-cortical pathways. We have previously addressed this issue in the **Discussion** paragraphs ‘**Mechanisms of EEG slowing in cortical disconnection**’, now placed right at the incipit of the discussion without a header, and ‘**Properties of post-disconnection slow waves**’).
- **24)** You could add the Wada test to this: "A vast literature on acquired brain-injury [...], hemispherectomy [...] and split-brain patients [...] suggests that one hemisphere is sufficient to support consciousness." Perhaps also the fact that several animals (e.g. migratory birds and dolphins) can have half their brain asleep while remaining vigilant and behaviorally awake.

>> We really appreciate the reviewer’s suggestions. We have accordingly integrated the text adding the example of the Wada test. We acknowledge the relevance of evidence from the animal literature showing that some species can remain behaviorally awake and fully conscious while one hemisphere is in a sleep state. Actually, the comparison with dolphins helped us to informally conceptualize our study very early on in the process.

However, in this context we have chosen to focus our discussion on human data, particularly from clinical models such as the Wada test, which offer a direct and controlled way to assess hemispheric contributions to consciousness in humans. While the animal literature provides compelling support for the notion that one hemisphere can sustain awareness, we preferred to ground our reasoning in findings directly applicable to the human brain, given the clinical and translational nature of our study.

*A vast literature on acquired brain-injury (Gschaidmeier et al., 2021; Asaridou et al., 2020; Sebastianelli et al., 2017), hemispherectomy (Dandy, 1928; Crockett and Estridge, 1951; Peacock et al., 1996; Lew, 2014; Fisher et al., 2022) and split-brain patients (Schechter and Bayne, 2021; de Haan et al., 2018) as well as studies conducted on subjects during the WADA test (Wada and Rasmussen, 1960; Loring et al., 2012; Halder et al., 2021) suggests that one hemisphere is sufficient to support consciousness.*

- **25)** I urge you to avoid this formulation: "unconscious states such as sleep and anesthesia". Both of these states are well known to have a lot of dreams, despite it being difficult to obtain reports of rich reports (for different reasons). Even in deep sleep, and surgical anesthesia, there are reports of dream reports (albeit, arguably, of less rich experiences than normal wakefulness) in a large percentage of awakenings.

>>  We reformulated the expression to avoid using the term ‘unconscious’ for sleep and anesthesia, and instead expressed the idea that these states have a reduced likelihood of obtaining conscious reports and, when reports occur, the experiences are less rich, which we refer to, for simplicity, as diminished consciousness. Accordingly:

*Yet, resting state networks, including the DMN, can be disrupted in conscious psychedelic states (Gattuso et al., 2023) and, crucially, preserved in ~~unconscious~~ states where consciousness is lost, or diminished, such as sleep and general anesthesia (Boly et al., 2008; Horovitz et al., 2008; Vincent et al., 2007; Akeju et al., 2014) making it difficult to infer awareness solely based on fMRI patterns*.

- **26)** Unfortunately, I have to disagree with the following statement: "the disconnected cortex rests in an electrophysiological state that is not compatible with the presence of an island of awareness". There are many situations where the cortex is in a state of slow waves where there is provably (to the extent that it is possible, relying on immediate reports following abrupt awakening). This must be weakened, or rephrased. As a minimal change (which changes the emphasis of what we can get evidence for from observational studies such as this one) you might say something like "the disconnected cortex rests in an electrophysiological state that does not provide strong evidence for the presence of an island of awareness". Note the switch from evidence against consciousness to lack of evidence for consciousness.

**>>** We fully agree that slow-wave activity can occur in conditions where some form of conscious experience may be present, and that electrophysiological data alone cannot confirm or exclude awareness definitively. In response to this concern, we have removed the original claim that such a state is "not compatible with the presence of an island of awareness." We now take into account in the discussion a position similar to that proposed by the reviewer, stating:

*“The present findings of prominent slow waves and steep spectral decay do not support the hypothesis that hemispherotomy may result in cortical ‘islands of awareness’.”*

- **27)** Unsurprisingly, I think the following is much to strongly phrased: "First, EEG slow waves are canonically associated with unconscious conditions encompassing NREM sleep[...], general anesthesia[...] and the vegetative state [...]." Again, all of these states are well known to be associated with conscious experiences.

>> We have dropped the classification of such states as unconscious, and adopt a more nuanced description. We have modified the rhetoric accordingly, while also taking into account the variety of positions expressed in previous literature

We revised this sentence in the **Discussion**:

*First, EEG slow waves are canonically associated with ~~unconscious~~ conditions encompassing NREM sleep (Jouvet, 1967; Nir et al., 2011; Pigorini et al., 2015), general anesthesia (Brown et al., 2010; Purdon et al., 2015; Murphy et al., 2011; Lewis et al., 2012; Sarasso et al., 2015) and the vegetative state (Comanducci et al., 2020; Colombo et al., 2023; Estraneo et al., 2016; Schiff, 2016; Lechinger et al., 2013; Rosanova et al., 2018)~~.~~; whereby conscious experiences are abolished (Purdon et al., 2013; Purdon et al., 2015; Lewis et al., 2012) or less likely and less rich (Nir and Tononi, 2010; Sanders et al., 2012; Bonhomme et al., 2019; Siclari et al., 2017; Sevenius Nilsen et al., 2022)*

Bonhomme V, Staquet C, Montupil J, et al. General Anesthesia: A Probe to Explore Consciousness. *Front Syst Neurosci*. 2019;13:36. Published 2019 Aug 14. doi:10.3389/fnsys.2019.00036

Lewis LD, Weiner VS, Mukamel EA, Donoghue JA, Eskandar EN, Madsen JR, Anderson WS, Hochberg LR, Cash SS, Brown EN, Purdon PL: Rapid fragmentation of neuronal networks at the onset of propofol-induced unconsciousness. Proc Natl Acad Sci U S A 2012; 109:E3377–86

Nir Y, Tononi G. Dreaming and the brain: from phenomenology to neurophysiology. Trends Cogn Sci. 2010;14:88–100. doi: 10.1016/j.tics.2009.12.001.

Purdon PL, Pierce ET, Mukamel EA, et al. Electroencephalogram signatures of loss and recovery of consciousness from propofol. Proc Natl Acad Sci U S A. 2013;110(12):E1142-E1151. doi:10.1073/pnas.1221180110

Purdon PL, Sampson A, Pavone KJ, et al. Clinical Electroencephalography for Anesthesiologists: Part I: Background and Basic Signatures. Anesthesiology. 2015;123(4):937-960. doi:10.1097/ALN.0000000000000841

Sanders RD, Tononi G, Laureys S, Sleigh JW. Unresponsiveness ≠ unconsciousness. Anesthesiology. 2012;116(4):946-959. doi:10.1097/ALN.0b013e318249d0a7

Sevenius Nilsen A, Juel BE, Thürer B, Aamodt A, Storm JF. Are we really unconscious in "unconscious" states? Common assumptions revisited. Front Hum Neurosci. 2022;16:987051. Published 2022 Oct 5. doi:10.3389/fnhum.2022.987051

Siclari F, Baird B, Perogamvros L, et al. The neural correlates of dreaming. Nat Neurosci. 2017;20(6):872-878. doi:10.1038/nn.4545

- **28)** The following interpretation seems more valid, but is in contrast with the stronger claims brought elsewhere: "indication of the reduced likelihood of dream-like experiences in the isolated cortex."

>> We appreciate the reviewer’s support for this more nuanced interpretation. In this passage, we aimed to emphasize the *reduced likelihood* of vivid, dream-like experiences in the isolated cortex, rather than making definitive or categorical claims—such as fully ruling out the possibility of conscious experience. This cautious framing is consistent with the main interpretative stance exposed throughout the manuscript. Accordingly, we take into account this perspective in other key sections of the discussion:

*“The broadband slowing, as indexed by the spectral exponent, closely matches that observed in deep NREM sleep—a state typically associated with a reduced likelihood of vivid conscious experiences...”*

This approach reflects our effort to remain scientifically grounded while acknowledging the limits of inference based on physiological data alone. These limitations are thoroughly expressed in the discussion paragraph **Limitations and Future directions,** **comment 32.** See also **comment 31.**

- **29)** Again, I think the following has the wrong emphasis: "provide evidence against the possibility that hemispherotomy may result in cortical islands of awareness." It is not that there is evidence against, but (at best) no evidence for, vivid/rich/wakelike/narrative experiences. Better would be something like saying the likelihood of receiving reports of experience (if it could give such reports) would be reduced. Meaning that there is still a (high!) probability that there are experiences, but that the kind and frequency would be more like those from deep sleep, anesthesia etc.

**>>** We thank the reviewer for this thoughtful observation. As previously pointed out by the reviewer, our findings cannot rule out the presence of residual conscious experiences. Accordingly, we mention that if any residual experiences are present, they are unlikely to be vivid, coherent, or dream-like in character. To better reflect this, we have revised the text to adopt a softer and more appropriate formulation. Specifically, we now state:

*In good agreement with perturbational measures of brain complexity (the Perturbational Complexity Index, see Casali et al., 2013), the spectral exponent robustly discriminated conscious states, including neurotypical wakefulness, vivid physiological or pharmacological dreams and the minimally conscious state, from conditions of diminished consciousness such as deep NREM sleep, general anesthesia and the vegetative state ~~between conscious and unconscious states, across wakefulness, sleep, physiological or pharmacological dreams, anesthesia induced by different compounds, and severe brain injury~~ (Colombo et al., 2019; Colombo et al., 2023; Maschke et al., 2023b; Casey et al., 2023). Finding spectral exponent values consistent with those observed in these latter conditions supports the inference that the disconnected cortex may not sustain vivid, dream-like experiences. ~~Overall, detecting an electrophysiological pattern aligned with that observed in reference physiological, pharmacological and pathological conditions in which the global state of consciousness is abolished or greatly reduced, provide evidence against the possibility that hemispherotomy may result in cortical islands of awareness.~~*

We emphasize that the revised phrasing — *“supports the inference”* — in this key passage of the discussion is deliberately more cautious than the earlier formulation, and aims to reflect the limitations of the data in drawing definitive conclusions about subjective experience.

- **30)** The paragraph starting with "Crucially, the disconnected cortex" nicely sums up my issue with that analysis. First, the "crucially" indicates that this is an important finding to understand/interpret the rest of the findings, even though no justification for this is given. Then it ends with the sentence "This result is unsurprising, as spindles are generated in the thalamus[...]" which undermines the need for this analysis. Again, I recommend reassessing the need for this analysis, beyond just confirming the success of the surgery. To me, it just muddles the paper.

>> After reflecting on the reviewer’s feedback, we agree that this analysis does not play a central role in the primary findings of the paper. We followed the reviewer’s advice and, as previously mentioned in response to **comment 13**, we have opted to relocate this section to the **Supplementary Material**, in line with the reviewer’s suggestion.

- **31)** I think this is too strong: "aligns with patterns observed in unconscious reference conditions, alleviating concerns about the presence of an "island of awareness" within the disconnected hemisphere." First, there is no direct evidence that the reference conditions were unconscious (a large proportion of dreams even in N3).
  Second, the extent to which it "aligns" is debatable, in part also, because of what you discuss earlier: pre surgery the disconnected hemisphere was like N1, without that leading us to believe there were N1-like experiences at that point.
  Third, at least to me, it doesnt "alleviate" much if anything wrt the possibility of islands of awareness. It does, perhaps, indicate that the experiences it might be having are more like those we commonly see in N2 sleep, deep propofol sedation, and the like (that is, dreams in upwards of 80% of awakenings, depending on methods for obtaining reports), but is that really evidence for unconsciousness and no islands of awareness? I dont think so.

**>>** We thank the reviewer for this thoughtful critique. We acknowledge that our reference conditions cannot be unequivocally classified as unconscious. Consistent with revisions made throughout the manuscript, we have avoided the use of the adjective “unconscious” to prevent implying a categorical dismissal of the possibility of residual conscious experience in these states. We now write:

“*The broadband slowing, as indexed by the spectral exponent, closely matches that observed in deep NREM sleep —a state typically associated with a reduced likelihood of vivid conscious experiences—and parallels that seen during deep anesthesia and in the vegetative state.*”

In the paragraph that follows, we acknowledge that inferences about consciousness based on EEG alone must be made cautiously, especially in brain regions that are behaviorally inaccessible. We then thoroughly elaborate on available evidence suggesting that some form of disconnected or residual consciousness may persist even in conditions traditionally labeled as “unconscious”. Please see answer to **comment 32.**

- **32)** The discussion immediately following this is better, but does not touch on the fact that most conditions classically (and incorrectly) assumed to be "unconscious" states, are in fact (as far as we can tell with gold standard approaches based on immediate reports) riddled with conscious experience. This should be made very explicit in this discussion**,** rather than just pointing to some rare genetic conditions. This is because it provides important context wrt drawing conclusions about whether there is good reason to believe there can be any awareness (or, rather, experiences) in the disconnected cortical islands.

>> We appreciate the reviewer’s feedback and have revised the discussion accordingly. In particular, we have avoided the term "unconscious" and expanded the final paragraph to acknowledge that states traditionally labeled as unconscious—such as deep sedation, NREM sleep, and the vegetative state—may still support forms of residual or disconnected consciousness, even though the EEG displays slow wave activity. This paragraph follows the revised sentence mentioned in the previous **comment 31**:

*However, ~~a general caveat of this conclusion is that~~ any inference about the presence or absence of consciousness, based solely on the brain's physical properties (including spontaneous EEG dynamics), must be approached with caution, particularly in neural structures that are not behaviorally accessible. It remains possible that some form of consciousness could persist even in the presence of highly prominent slow waves in the spontaneous scalp EEG, as revealed by peculiar genetic conditions, i.e. Angelman syndrome (Frohlich et al., 2021), and by transient, disconnected psychedelic states (Blackburne et al., 2024). More broadly, growing evidence suggests that some form of disconnected, residual consciousness may persist in states traditionally labeled as “unconscious”—such as deep sedation, NREM sleep, and the vegetative state—despite the presence of slow EEG activity [Siclari et al., 2013; Siclari et al., 2017 Nir and Tononi, 2010; Sanders et al., 2012; Bonhomme et al., 2019; Sevenius Nilsen et al., 2022]. This possibility warrants further exploration. (...)*

The discussion that follows provides context and outlines how future hemispherotomy studies could offer stronger evidence to inform the ongoing debate around the ‘island of awareness’ hypothesis (see paragraph **‘Limitations and future directions’**).

- **33)** I am missing a conclusion of the overall, main findings of the study to wrap up the discussion section.
  >> We thank the reviewer for this valuable suggestion. In response, we have added a clear and concise concluding paragraph at the end of the Discussion, summarizing the main findings of the study. This new conclusion highlights the persistent neurophysiological reorganization observed in the isolated cortex after hemispherotomy, the role of subcortical (thalamic) inputs leading to the observed changes (see **comment 23**), and the alignment of the electrophysiological state of the isolated cortex’s with deep NREM sleep, as indexed by the spectral exponent. This finding indicates a unihemispheric sleep-like state during wakefulness, and is consistent with the potential absence of conscious experiences, or with a reduced state of awareness (see **comment 28**).

***Conclusions***  *Our findings demonstrate that hemispherotomy induces a lasting reorganization of neurophysiological activity in the isolated cortex toward a state characterized by prominent slow waves and a marked broadband spectral redistribution toward lower frequencies. Characterizing this pattern by the spectral exponent, we detected values aligned to those observed during pediatric deep NREM sleep, indicative of a unihemispheric sleep-like state during wakefulness, and consistent with absent or reduced conscious experiences.*

*The persistence of slow waves, lasting months to years after surgery, raises the question of whether they play any functional role or merely reflect a regression to a default mode of cortical activity.*

Reviewer #2

The study "Hemispherotomy: a cortical island of sleep-like activity in aware humans" by Colombo and colleagues subjects ten pediatric hemispherotomy patients to pre- and postoperative EEG-analysis. While a myriad of research studies have analyzed preoperative clinical, psychological, electrophysiological and imaging predictors in hemispherotomy candidates (Staudt et al., 2002; Gaubatz et al. 2020; Ramantani et al., 2024 and others), the fate of the isolated hemisphere has not received scientific attention. This is interesting as subjects who underwent the (less invasive) callosotomy have been in the center of decade-long neuroscientific and neurophilosophical research endeavours (Nagel, 1974; Wolman, 2012 and others). Only recently, the isolated hemisphere of individuals after hemispherotomy has been re-introduced in the debate by one of the study's coauthors (Bayne, 2020), when their capacity to be "islands of awareness" has been suggested. This most interesting claim, however, could not be substantiated due to a lack of empirical data. These data are now provided in this **most spectacular** study.  This is a concisely written manuscript involving a very rare study collective, longitudinal data and most elaborate EEG-analysis. It finds large-amplitude slow wave activity and an overall redistribution of power spectral density from high to low frequencies in the isolated hemisphere. Interestingly, a lack of spindle and slow-delta activity with smaller amplitude is found, indicative of disconnected thalamo-cortical connections. The EEG-findings resemble sleep and, thus, question the claim that the isolated hemisphere after hemispherotomy holds a potential to be an island of awareness. The emerging scientific debate is being steered in a new direction by these results, for which the authors deserve great credit. I had several minor revisions after reading the study, only to find that they had almost all been answered by the authors in the most extensive supplementary material. The study's limitations are almost completely addressed in the limitations-and-future-directions-paragraph of the manuscript (such as the role of the pathological hemisphere in the constitution of consciousness before surgery).

1. An additional limitation in all hemispherotomy studies is the clinical heterogeneity of the individuals regarding etiology, age at surgery and type of surgery.

**>>** We thank the reviewer for this thoughtful and appropriate comment.

We now display a **Supplementary Figure 7** that replicates main **Figure 2,** while also revealing at once the type of etiology (cortical malformation, perinatal ischemic stroke) and the type of hemispherotomy (whole-hemisphere, temporo-parieto-occipital) for each patient.

According to qualitative inspection, all subgroups of patients followed the overall pattern described in the main text, however, given the rarity of hemispherotomy patients, our limited sample size does not allow strong statistical inferences about the impact of etiology and the type of hemispherotomy.

For completeness, we report below and in the **Supplementary Material, section S6** ***Controlling for Etiology and Type of Hemispherotomy*** the proportion of patients in each subgroup that followed the pattern described in the main text, with respect to the inter-hemispheric spatial asymmetry after surgery and to the pre-post temporal change of the affected cortex.

***S6 Controlling for Etiology and Type of Hemispherotomy***
*We report below the ratio of patients in several subgroups that followed the pattern described in the main text, namely, the finding of an inter-hemispheric spatial asymmetry after surgery and a pre-post temporal change within the pathological cortex (see main results). Moreover, we display in* ***Supplementary Figure 7*** *the EEG features (slow delta power and spectral exponent) of the patient dataset (in the disconnected and contralateral cortex), along with their pre-post change (as in main* ***Figure 2****), while also revealing at once the type of etiology (cortical malformation, perinatal ischemic stroke) and the type of hemispherotomy (whole-hemisphere, temporo-parieto-occipital) for each patient.*

***Etiology***

*Considering the amount of slow-delta power (0.5-2 Hz), 4/4 patients with a cortical malformation displayed spatial asymmetry after surgery, and 4/4 patients showed a pre-post increase. Further, 5/6 patients with pre or perinatal ischemic stroke displayed spatial asymmetry after surgery and a 5/6 showed a pre-post increase.*

*Considering the spectral exponent (0.5-20 Hz), 4/4 patients with a cortical malformation displayed spatial asymmetry after surgery and 4/4 patients showed a pre-post decrease. Further, 6/6 patients with pre or perinatal ischemic stroke displayed spatial asymmetry after surgery and a 6/6 showed a pre-post increase.*

***Type of hemispherotomy***

*Considering the amount of slow-delta power (0.5-2 Hz), 3/3 patients with temporo-parieto-occipital disconnection displayed spatial asymmetry after surgery and a 3/3 showed a pre-post increase. Further, 6/7 patients with hemispherotomy of a whole-hemisphere displayed spatial asymmetry after surgery, and 6/7 patients showed a pre-post increase.*

*Considering the spectral exponent (0.5-20 Hz), 3/3 patients with a temporo-parieto-occipital disconnection displayed spatial asymmetry after surgery and 3/3 patients showed a pre-post decrease. Further, 7/7 patients with hemispherotomy of a whole-hemisphere displayed spatial asymmetry after surgery and a 7/7 showed a pre-post increase.*

***Supplementary Figure 7. Narrow- and broad-band spectral changes after surgery reveal a marked EEG slowing of the isolated cortex, robust across etiology and type of hemispherotomy*** *This figure mirrors* ***Figure 2*** *from the main text, graphically highlighting the different patients in terms of etiology and type of disconnection. Specifically, patients who underwent complete hemispherotomy are marked with a circle, while those who underwent temporo-parieto-occipital disconnection are marked with a square. Additionally, patients with a cortical developmental malformation as the underlying etiology are shown in red, whereas those with pre- or perinatal stroke sequelae are shown in green.* ***A****) Slow Delta Power (0.5-2 Hz) was significantly larger in the disconnected than in the contralateral cortex only in the session after surgery (****Figure 2A****). This pattern was observed in 4/4 patients with a cortical malformation, 5/6 patients with pre/perinatal ischemic stroke, 3/3 patients with temporo-parieto-occipital disconnection, 6/7 patients with hemispherotomy* ***B)*** *Slow Delta Power increased significantly pre to post surgery only in the disconnected cortex (****Figure 2B****); this pattern was observed in the same subset of patients described above, across the subgroups of etiology and type of hemispherotomy.*

***C)*** *The spectral exponent (0.5-20 Hz) was significantly more negative in the disconnected than in the contralateral cortex in the session after surgery* ***(Figure 2C).*** *This pattern was observed in all conditions: in 4/4 patients with a cortical malformation, 6/6 patients with pre/perinatal ischemic stroke, 3/3 patients with temporo-parieto-occipital disconnection and 7/7 patients with hemispherotomy.* ***D)*** *The PSD became significantly steeper from pre to post surgery only in the disconnected cortex, as indexed by the spectral exponent (****Figure 2D)****; this pattern was observed in all the patients, across the subgroups of etiology and type of hemispherotomy.*

Furthermore, we directly addressed the potential impact of age on narrow-band and broad-band EEG slowing by conducting extensive additional analyses, using age-matched subjects from the reference dataset, which confirmed the robustness of our main findings. Please see also the answers to **comment 14** of reviewer 1, and to **comment 2** of reviewer 3, and the new **Supplementary Figures 10, 11, 15** from the new sections of the **Supplementary Material S9  and S12**

Regarding the details of the type of surgery, unfortunately this information was not available for all patients; we have therefore acknowledged this point as a limitation in the revised manuscript.

In the **Methods** of the main text we now mention:

*Moreover, we evaluated whether etiology and type of hemispherotomy had any influence on the main findings (****Supplementary Material section S6****).*

In the **Results** of the main text we now mention

*In supplementary control analysis, neither the etiology (cortical malformation, perinatal stroke) nor the type of hemispherotomy (whole-hemisphere, and temporo-parieto-occipital disconnection) appeared to influence the main results, as all subgroups qualitatively showed the same post-surgical asymmetry and pre–post changes in the disconnected cortex observed at the group level (****Supplementary Material, section S6 )****.*

In the **Limitations and future directions** section of the main text we now mention:

*This study included only two disconnection types (hemispherotomy of an entire hemisphere and temporo-parieto-occipital disconnection) and did not consider the details of the surgical procedure; larger and more heterogeneous cohorts could clarify the roles of surgical technique, lesion volume, and disconnection topography in shaping electrophysiological outcomes.*

2. While authors provide subgroup analysis and a mixed effects model in the supplement, they have not used the lesion volume and location as a covariate in their analysis which would have been of interest.
   **>>**We thank the reviewer for this interesting consideration.
   Unfortunately, in this neurophysiological study we did not have a quantitative assessment of the lesion volume stemming from neuroimaging data (MRI scans). Nonetheless, some considerations can be drawn by closely inspecting **Figure 2** and the new **Supplementary Figure 7.** Patients that underwent temporo-parieto-occipital disconnection present a substantially smaller extent of isolated cortex induced by the surgical procedure; however, they followed the same overall pattern of those that received hemispherotomy of a full hemisphere, i.e. all three patients showed both spatial asymmetry after surgery and a pre-post change induced by the surgery, with respect to either narrow-band slowing (slow-delta power) or broad-band slowing (spectral exponent). See the answer to the previous comment for details.

Concerning lesion location, as mentioned our cohort includes only two disconnection types— hemispherotomy of a whole hemisphere and temporo-parieto-occipital disconnection—limiting the possibility for further stratification (e.g., anterior vs. posterior disconnection). Future studies involving a broader range of disconnection patterns may provide deeper insight into the effect of lesion location.

Finally, we performed a mixed effect model analysis including the type of hemispherotomy as a covariate, and found no significant effect of the type of hemispherotomy (and neither any interaction with it was significant), while time and, crucially, the interaction of time and spatial Side remained significant as in the mixed effect model that did not include such covariate (**Supplementary Material section S6.4**), for either of the two EEG features. We would like to note that, given the limited sample size, mixed-effects models incorporating a large number of factors should be interpreted with caution. Still, we now add a new section to the Supplementary Material:	**S6.4 Mixed effect models including ‘type of hemispherotomy’ as a covariate**
*To account for the potential contribution of lesion extension, we re-estimated the mixed-effects models for both spectral exponent and slow-delta power including type of hemispherotomy (whole hemisphere vs temporo-parieto-occipital disconnection) as a covariate, and assessed fixed effects using marginal ANOVA.

For Slow Delta power, the inclusion of the type of hemispherotomy did not alter the pattern of effects observed previously. The updated model revealed no significant main effect of spatial side (F(1, 32) = 0.25, p = 0.617), nor of the type of hemispherotomy (F(1, 32) = 1.74, p = 0.197), but confirmed a significant main effect of time (F(1, 32) = 8.11, p = 0.0076). Critically, the interaction between spatial side and time remained significant and robust (F(1, 32) = 13.80, p = 0.0008), consistent with the previous model. No significant interactions were observed involving type of hemispherotomy (all p > 0.45), indicating that lesion extension did not modulate the spatial or temporal dynamics of slow-delta activity.
Similarly, for the spectral exponent, the inclusion of the type of hemispherotomy did not alter the pattern of effects observed previously. As in the original model, we found a significant main effect of spatial side (F(1, 32) = 5.41, p = 0.026), a strong effect of time (F(1, 32) = 18.03, p < 0.001), and a large, highly significant interaction between side and time (F(1, 32) = 51.89, p < 0.000001). The type of hemispherotomy itself had no significant main effect (F(1, 32) = 0.39, p = 0.538), and its interactions with side and time did not reach significance (all p > 0.07), although the interaction with time showed a statistical trend (F(1, 32) = 3.44, p = 0.073).
Altogether, these findings indicate that using type of hemispherotomy as a covariate in a mixed effect model does not alter the core spatiotemporal effects observed for each of the two EEG spectral features; hence lesion size is not a major contributor to the interindividual variance in this context. Nonetheless, future studies incorporating larger sample sizes and a broader range of disconnection types with different lesion volumes and locations may uncover subtle modulatory effects on the electrophysiological slowing induced by hemispherotomy.*

In the main text we now mention this specific analysis in the Results sections as follows:

*We moreover verified that the extent of disconnected cortex did not play a significant role, by means of a mixed effect model analysis including the type of hemispherotomy as a covariate (****Supplementary Material, section S6.4****)*

We now explicitly acknowledge the limitations highlighted in the two comments above in the revised **Limitations and future directions** section (please see the answer to previous comment).

Reviewer #3

In this study Colombo et al investigated EEG activities in young human epileptic patients before and after complete isolation of cortex or large part of cortex in one hemisphere that was responsible for the seizure onsets. The authors report that isolated large parts of the cortex show continuous slow wave activities that are reminiscent, but not identical to slow wave activities recorded during slow wave sleep. Based on previous animal experiments, such a finding could be expected but it was never demonstrated in human. Therefore, this study provides fundamental new information on electrographic behavior of isolated human cortex. Bravo.

Despite my great enthusiasm about this study, I have several general comments which, if addressed, would likely improve the presentation of the results, and I have also some specific/minor comments which point to some small errors or unclearly described aspects of the manuscript.

General comments.

1) Essentially you compared EEG in a 'pathological' hemisphere before and after disconnection with contralateral hemisphere without specifying in each case the state of vigilance with control population in which you specify W, N1, N2 and SWS. I was continuously looking for a vigilance state in your patient population. So, ideally, you should present data in your patient group with specified W, N1, N2 and SWS. If you do not have such data because such recordings were clinically not justified, I would still recommend mentioning in each figure legend that your patient recordings were done during wake.

**>>** We thank the reviewer for allowing us to point out this important clarification. The fundamental premise of our study is that the electrophysiological analysis of the disconnected cortex was performed using EEG signals recorded while subjects were in a behavioral state of wakefulness. Indeed, our key finding is a profound dissociation, whereby the disconnected cortex is engulfed in prominent slow waves resembling those of deep sleep while the subject, and rest of the brain are awake. We emphasize this aspect in the title ("in awake humans"); however, we acknowledge that it was not consistently stated throughout the manuscript. We have now revised the text to explicitly clarify, in multiple sections, that all recordings from hemispherotomy patients were obtained while the subjects were behaviourally awake.
Some examples of these clarifications are reported below:

**Abstract**

- *We address these questions by exploring for the first time the electroencephalographic (EEG) state of the isolated cortex during wakefulness before and after surgery in ten pediatric patients*

**Introduction**

- *Here, we fill this gap by analyzing the EEG background–i.e. free of epileptiform discharges–recorded ~~studying for the first time regional EEG patterns~~ during wakefulness in ten pediatric participants who had undergone a complete cortical disconnection*

**Materials and methods** 

- *(inclusion criteria) presence of at least 3 min of quiet wakefulness free from artifacts and interictal epileptiform discharges.*
- *During the EEG the participants, lying awake on a bed or in the arms of the parent, did not perform any particular task*
- *Each EEG recording was* *performed in wakefulness and gathered from* *~~obtained by~~* *19 scalp electrodes (cup in chlorinated silver) positioned according to the international 10–20 system and a 32-channel amplifier (Neurofax EEG-1200, Nihon Kohden Corporation).*
- *In the previous part of the study we compared the spectral exponent in behaviourally awake patients before and after surgical disconnection to that of a reference pediatric cohort across wakefulness and NREM sleep stages.*
- *After observing that in awake patients the broad-band spectral exponent of the disconnected cortex closely resembled that of deep NREM sleep.*

**Results**

- *The EEG recordings were performed in awake patients within 12 months before surgery (mean=5.6, std = 4.67, range = [1, 12]) and at least six months after surgery (delay in months: mean=19.8, std=9.6, range = [6.96, 34.92]).*
- *The visual inspection of the EEG time-series recorded in an awake patient and of the topographies of low-frequency PSD (Slow Delta power, 0.5-2 Hz) reveal high-amplitude (>50 microvolts) slow waves emerging over the disconnected cortex post-surgery, associated with pronounced inter-hemispheric asymmetry*
- *As shown in Figure 2, at the population level, in awake patients, the disconnected cortex following surgery displayed increased Slow Delta power and an overall PSD redistribution towards low frequencies and a steeper broad-band decay, as evident from the PSD and its 1/f-like fit, aggregated across patients.*
- *Finally, the spectral exponent values assessed in awake patients before and after cortical disconnection were compared to the values assessed in a reference pediatric sample during wakefulness and NREM sleep*
- *We further characterized slow wave activity occurring during wakefulness in the disconnected cortex [...]*
- *Overall, the disconnected cortex in awake patients showed a remarkable emergence of Slow Delta activity, leading to a state departing from wakefulness, yet different from full-fledged NREM sleep*

1.1) More, if all your recordings in patients were done in wake, than a part of your study related spindle analysis has no meaning because spindles during wake are not generated. Then, I would strongly recommend removing the part of your manuscript related to spindles. With or without isolation, spindles would not be generated.

>> After thoughtfully considering the reviewer’s comment, we decided to follow the reviewer’s advice and moved the lengthy section on spindle analysis to the Supplementary Material. Such findings are now briefly described and discussed in the main text.

We now mention in the main method section:
‘*Given the established thalamic origin of sleep spindles, we confirmed their absence in the disconnected cortex through multiple assessments: visual inspection by a trained neurologist, analysis of total and periodic power in the sigma band, and automated detection of sigma-band bursts (****Supplementary Material, section S14 Sigma oscillations and spindle activity).’***

In the result section:

*‘Overall, the disconnected cortex showed a remarkable emergence of slow-delta activity, leading to a state departing from wakefulness, yet different from full-fledged NREM sleep, insofar the disconnected cortex did not display any spindle activity, in line with the thalamic disconnection and with the established thalamic origin of sleep spindles.’*

In the discussion section:
*As expected under conditions of thalamic disconnection, post-surgery slow waves were not accompanied by spindle activity (according to visual inspection, sigma burst detection, and spectral analysis of total and periodic power,* ***Supplementary Material, section S14*** *)*

**2.**	All your data presented as average across all EEG electrodes and patients. This give you higher statistical power, but there are some drawbacks too.

**2.a**	It is known that in 2-year-old children that slow wave activity is highest in the occipital cortex and in 10-year-old children the highest power is in central and frontal regions (Kurth S, Ringli M, Geiger A, LeBourgeois M, Jenni OG, Huber R. Mapping of cortical activity in the first two decades of life: a high-density sleep electroencephalogram study. J Neurosci. 2010; 30 (40): 13211-13219.).

**2.b**	As you previously demonstrated the sleep slow waves propagate. There are also animal data that cortical slow wave synchrony after isolation or thalamic inactivation is reduced thus propagation velocity is reduced (you cited Lemieux et al., 2014, but they also have follow up study on that topic (Lemieux M, Chauvette S, Timofeev I. Neocortical inhibitory activities and long-range afferents contribute to the synchronous onset of silent states of the neocortical slow oscillation. J Neurophysiol. 2015; 113 (3): 768-779).
Based on these two facts, your conclusions on properties of slow waves in isolated cortex could be erroneous. If you average slow waves that propagate slower in one case as compared to control case, or propagate from posterior to anterior or central to peripheral regions without known velocity, you can obtain averaged data showing smaller slow wave amplitudes, different periodicity, etc.

>> We thank the reviewer for this insightful and important comment. We acknowledge the relevance of the works by Lemieux et al., including the 2015 study that highlighted the key role of long-range afferents in the synchronization and propagation of cortical slow oscillations. Indeed, the loss of long-range connectivity—a hallmark of the disconnected cortex following hemispherotomy—could alter the propagation dynamics of cortical slow waves, leading to a reduction in their velocity or even to localized generation with reduced synchrony.

These mechanisms give a valuable perspective to interpret our findings. For clarity, we note that slow wave properties were extracted from individual electrodes and then averaged across the channels (with the sparse spatial sampling typical of clinical recordings)—not from spatially averaged voltage signals. We observed that the disconnected cortex exhibited slow oscillations with lower amplitude and a longer period compared to physiological sleep.

As the reviewer points out, when slow waves originate from multiple asynchronous cortical generators and propagate more slowly over the cortical surface, the resulting waveforms  detected over a scalp electrode tend to have reduced amplitude and a longer duration due to spatial and temporal desynchronization. We have now incorporated this important mechanistic perspective into the **Discussion,** as it offers a plausible explanation for the observed findings

*The reduced amplitude and longer period of slow oscillations in the disconnected cortex may also reflect cortical dynamics with reduced propagation velocity. Animal studies suggest that cortical isolation or thalamic inactivation reduces neuronal synchrony and slows the propagation of slow waves (Lemieux et al., 2015). Without long-range thalamic afferents, slow oscillations may arise from multiple asynchronous local cortical sources, leading to a spatiotemporal dispersion of slow waves. This low synchrony at the cortical surface level can contribute to the finding of EEG slow waves from the disconnected cortex having lower amplitude and longer duration than those from physiological sleep.*

**2.b.1**	Since you have only 10 patients from 2.5 to almost 12 years of age, it would be difficult to make groups, but you can do plots showing measured parameters of slow waves of individual patients on one axes and age on a second axes.

>> We thank the reviewer for the insightful comment pointing at the potential impact of age on EEG slowing in our patients dataset. We found no significant correlation between age and EEG features in our patient dataset (reported in the new **Supplementary Figure 9)**. Beyond marginal trends observed for the spectral exponent in the contralateral cortex, no significant correlations with age were found in the disconnected hemisphere, likely due to the high inter-individual variability typical of severe epilepsy—particularly evident in the affected cortex.

Moreover, given the known impact of age on slow-wave activity and the spectral exponent in healthy pediatric populations (Kurth et al., 2010, Favaro et al., 2023), we sought to verify whether the findings in the patient dataset relative to the comparisons with wakefulness and sleep from the reference dataset (displayed in **Figure 3** and **Figure 4**) would hold after controlling for age.

Specifically, we sought to confirm that the electrophysiological profile of the disconnected cortex resembled deep NREM sleep (main **Figure 3**), beyond any potential effects of age. We thus applied a pairwise age-matching approach (illustrated in **Supplementary Figure 10**) to compare spectral exponent values of the patient dataset to those of the pediatric reference dataset across wakefulness and NREM sleep stages (**Supplementary Figure 11**).This analysis confirmed that the spectral exponent values of the disconnected cortex resembled N1 sleep before surgery and shifted toward values characteristic of deeper NREM stages (N2–N3) after surgery, independently of age.

Moreover, we extended the same age-matching approach to the analysis of the amplitude and period of slow-delta oscillations, comparing the disconnected cortex to pediatric reference values during wakefulness and NREM sleep (**Supplementary Figure 15**). This analysis replicated the core results reported in **Figure 4**, showing that the disconnected cortex exhibited amplitude values intermediate between wakefulness and NREM sleep (with larger differences observed relative to wakefulness), and significantly longer oscillation period than either condition, independently of age.

We accordingly report these new figures and analyses in new sections **(S9 and S12)** of the Supplementary Material (highlighted by underlined titles) as follows:

***Supplementary Figure 9. Correlation analysis among hemispherotomy patients revealed the absence of significant correlations between age and EEG features, either slow-delta power or spectral exponent.*** *The left panel of the figure shows that both before (top row) and after (bottom row) disconnection, in both the disconnected hemisphere (right) and the contralateral one (left), there is no correlation between age and spectral exponent. The right panel similarly shows that neither before nor after disconnection, in either hemisphere, is there a correlation between age and the slow-delta band envelope. Beyond marginal trends observed for the spectral exponent in the contralateral cortex, no significant correlations with age were found in the disconnected hemisphere, likely due to the high inter-individual variability typical of severe epilepsy—particularly evident in the pathological cortex*

***S9 Supporting age-matched comparison of spectral exponent between Patient and Reference datasets***

*Given the well-known impact of age on EEG features (Kurth et al., 2010; Gorgoni et al., 2020; Favaro et al., 2023), we conducted supplementary analyses to factor out the effect of age in our findings related to the comparison between the patient and the pediatric reference dataset. Primarily, we sought to verify if the electrophysiological profile of the disconnected cortex resembled deep NREM sleep (main* ***Fig. 3****), beyond any potential effects of age. Accordingly, we applied a pairwise age-matching approach (illustrated in* ***Supplementary Figure 10)*** *to compare the electrophysiological profiles of the patient dataset to those of the pediatric reference dataset across wakefulness and NREM sleep stages (****Supplementary Figure 11****).*

**Supplementary Figure 10. *Matching subjects by age across the Patient and the Reference dataset.*** *The figure shows age-matching across the patient dataset and a subset of the reference dataset; with each subplot comparing the age of patients to that of matched control subjects from the pediatric reference dataset, separately for pre- and post-surgery. The x-axis represents subject number (sorted by age), and the y-axis shows age in years. In both panels (Pre-Surgery and Post-Surgery), each red dot (patient) is paired with a nearby blue dot (reference subject), indicating that age-matching was performed on an individual basis. The dashed lines connecting each pair indicate pairwise matching, and the vertical distance shows the age difference between each pair. All patients were age-matched within approximately 1 year or less, which supports a robust age-controlled comparison. Such tight age matching was meant to ensure that any differences observed between patients and the whole reference data (e.g., in spectral slope or slow-wave activity) would not be driven by age-related changes.*

*Once age-matched subjects were identified, we compared the spectral exponent values between the patients dataset (in the disconnected or contralateral cortex, before or after surgery) and the the subsample of the pediatric reference dataset (either during wakefulness, N1, N2, or N3 sleep) (****Supplementary Figure 11****), by computing the distribution of pairwise differences between age-matched subjects. We then identified the vigilance states showing the greatest similarity through a bootstrap analysis of the mean pairwise differences across conditions (violin plots in* ***Supplementary Figure 11  B,D,F,H****).*

*We first qualitatively assessed how many hemispherotomy cases showed a steeper spectral decay than their age-matched counterparts during deep NREM sleep in the neurotypical reference dataset. Thus, we considered the proportion of age-matched cases in the patient dataset that showed a negative difference relative to N2 and N3 sleep of the pediatric reference dataset, indexing a steeper spectral decay in patients.The contralateral cortex did not exhibit a steeper spectral decay relative to either N2 or N3 sleep in any case (F,H). Before surgery, the pathological cortex showed a steeper spectral decay—indexed by negative differences—relative to N2 sleep in 2/10 cases and to N3 sleep in 0/10 cases (B). After surgery, the disconnected cortex showed a steeper spectral decay relative to N2 sleep in 9/10 cases and to N3 sleep in 3/10 cases (D).*

*For the contralateral cortex, before and after surgery alike, the distribution of pairwise differences was centered between wakefulness and N1 sleep values; the bootstrap distribution of mean pairwise differences indicated larger similarity with wakefulness and N1 sleep (****Supplementary Figure 11 F and H****), revealing a mild slowing but still distinct from deeper NREM stages.*

*For the pathological cortex before surgery, the distribution of pairwise differences was centered tightly around zero relative to N1 sleep values; the bootstrap distribution of mean pairwise differences indicated the greatest similarity to light (N1) sleep (****Supplementary Figure 11 B****).*

*For the disconnected cortex after surgery, the distribution of pairwise differences was centered between N2 and N3 sleep values; the bootstrap distribution of mean pairwise differences indicated larger similarity with deep (N2-N3) sleep (****Supplementary Figure 11 D****).*

***Supplementary Figure 11, Age-matched analysis comparing spectral exponent values in hemispherotomy to the pediatric reference dataset across wakefulness and NREM vigilance stages.*** *Each column presents two plots corresponding to a condition of the Patient dataset (Left to Right: Disconnected cortex pre- and post-surgery, followed by Contralateral cortex pre- and post-surgery), compared to each vigilance stage of the pediatric Reference dataset (wakefulness, N1, N2, N3, colored in shades spanning from green to blue). The first row* ***(A,C,E,G)*** *shows the absolute spectral exponent values, with lines connecting age-matched subjects across the Patient and Reference datasets. The second row* ***(B,D,F,H)*** *displays pairwise differences in spectral exponent: each dot represents the difference for a subject pair, calculated by subtracting the value of a subject in a given vigilance stage of the Reference dataset from that of the corresponding subject in the Patient dataset. Violin plots depict the bootstrap distribution of mean pairwise differences across subjects, thereby providing an estimate of the expected mean distance between paired conditions.
Overall, age-matched results revealed a mild slowing in the contralateral cortex, both pre-surgery (****E,F****) and post-surgery (****G,H****), with spectral exponent values intermediate between wakefulness (W) and N1. Importantly, age-matched results replicated the main findings (****Fig. 3****): spectral exponent values in the Disconnected cortex pre-surgery closely aligned with those observed during N1 sleep (****A,B****), while post-surgery values were intermediate between N2 and N3 sleep* ***(C,D)****.*

These supplementary results are briefly mentioned in the **results** section of the main text as:

*Age-matched analysis further strengthened this result, revealing that the disconnected cortex showed lower spectral exponent values in all 10 cases compared to their reference counterparts during both wakefulness and N1 (Supplementary Material S9, Supplementary* ***Figure 11****).  (...)*

*Supplementary age-matching analysis confirmed these findings (Supplementary Material S9, Supplementary Figure 11); regardless of age, while the spectral exponent values in the disconnected cortex resembled those of N1 sleep before surgery, they shifted toward values characteristic of deeper NREM stages (N2–N3) after surgery.*

Accordingly, in the **discussion** section, we now mention:

*Age-matched analysis further supported this finding, revealing lower spectral exponent values in all 10 cases compared to their reference counterparts during both wakefulness and N1 sleep (* ***Supplementary Figure 11****)*

We have included a new supplementary section to address potential age-related biases in the comparison of slow-wave properties with the reference dataset:

***S12  Supporting age-matched comparison of slow wave properties between Patient and Reference datasets***
*To further validate the analysis on slow wave properties of the disconnected cortex, we adopted the same age-matching approach detailed above (****Supplementary Figure 10****) and applied it to the analysis presented in* ***Figure 4*** *(Mean amplitude of the slow-delta envelope and period of slow-delta oscillations in the disconnected hemisphere, compared with respect to wakefulness and NREM sleep in the reference pediatric sample). This analysis corroborated the main results on slow wave properties of the disconnected cortex in relation to pediatric reference values, by controlling for the effects of age.*

*The amplitude of the slow-delta envelope of the disconnected cortex showed intermediate values between those of wakefulness and NREM (N2 and N3 jointly considered,* ***Supplementary Figure 15 A****, consistently with the main results in* ***Figure 4A****); through age-matching its values resulted significantly larger than those of wakefulness ( T(9)=3.741, p = 0.0046, with 9/10 subjects showing a consistent pairwise difference) and not significantly different from those of NREM sleep (T(9)=-1.633, p = 0.13, with 8/10 subjects showing a consistent pairwise difference,* ***Supplementary Figure 15 B****).*

*Further, the period of slow-delta oscillations of the disconnected cortex showed values higher than those of both wakefulness and NREM (****Supplementary Figure 15 C****, consistently with the main results in* ***Figure 4B****), through age-matching its values resulted significantly larger than those of both wakefulness (T(9)=4.783, p = 0.001, with 10/10 showing a consistent pairwise difference) and NREM sleep (T(9)= 3.686, p = 0.005, with 10/10 showing a consistent pairwise difference,* ***Supplementary Figure 15 D****).*

*This analysis replicated the core results concerning the properties of slow-delta activity reported in* ***Figure 4****: the disconnected cortex showed amplitude values intermediate between wakefulness and NREM sleep–with larger differences observed relative to wakefulness than to sleep–and significantly longer oscillation period than either condition, independently of age.
Together, these results strengthen the age-independent validity of our findings.*

**Supplementary Figure 15. *Age-matched analysis of amplitude and period of slow-delta oscillations in the disconnected cortex compared to the reference pediatric dataset during wakefulness and NREM sleep.*** *Results are drawn from the disconnected cortex in all 10 hemispherotomy patients age-matched to a subset of subjects from the reference dataset. (****A–B****) Slow-delta envelope amplitude comparison across datasets. (****A****) Boxplots show the amplitude of the slow-delta envelope in the disconnected cortex compared to wakefulness and NREM sleep in the reference dataset, with lines connecting age-matched subjects across datasets.(****B****) Pairwise differences after age-matching, relative to wakefulness and NREM sleep.*

*(****C–D****) Mean period of slow-delta oscillations comparison across datasets. (****C****) Boxplots show the mean period in the disconnected cortex compared to the reference dataset, with lines connecting age-matched subjects across datasets. (****D****) Pairwise differences after age-matching.*

*Overall, the disconnected cortex exhibited slow-delta amplitude values intermediate between wakefulness and NREM sleep, and period values significantly longer than both wakefulness and NREM sleep, consistent with the findings reported in* ***Figure 4*.**

**2.b.2** To address the issue of regional specificity, you can average data obtained over a few electrodes located over frontal lobe vs. occipital lobe etc. If you would do such or similar analysis and obtain information that is congruent with your current findings, it will reinforce your point regarding slow wave properties.
If by whatever reason, you cannot do such analysis, you can add a brief paragraph in you limitation section describing this as a limitation.

>> We appreciate the suggestion of the reviewer that prompted a set of new supplementary analysis to verify whether global findings on slow wave properties were also observed within two regions of interest (**Supplementary Material, section S13**). Specifically, we considered separately anterior and posterior regions in those subjects that received a hemispherotomy of a whole-hemisphere (N=7), and performed the main analysis on slow delta power in the patient dataset, to verify whether hemispherotomy led to unihemispheric slowing in both regions (New **Supplementary** **Figure 16**). For consistency, we also report the results of this regional analysis performed on the spectral exponent values.

Then, adopting the same age-matching approach outlined before, we assessed potential regional specificities when comparing the disconnected cortex (in either posterior or anterior regions) to reference the pediatric sample (New **Supplementary Figure 17**).
Consistently with the main findings obtained across the whole disconnected cortex, unihemispheric slowing after hemispherotomy was observed across anterior and posterior regions. Further, slow-delta activity in the disconnected cortex showed values of amplitude and period intermediate between wakefulness and NREM sleep, in both anterior and posterior regions (**Supplementary Figure 17 A,C**). We detail all the new supplementary analyses below:

***S13 Regional aspects of EEG features among hemispherotomy patients.***

*Given that the hotspot with maximal slow wave activity migrates from posterior to anterior regions in healthy children (Kurth et al., 2010) one may ask if the main findings can be observed even within two regions of interests, respectively considering a subset of electrodes in the anterior and posterior regions, in the subset of patients that received a hemispherotomy of a whole-hemisphere (N=7), thereby excluding those with a hemispherotomy of the parieto-occipito-temporal area (N=3). In our low-density montage set-up with bipolar electrodes, we thus selected four bipolar derivations for the anterior region (Fp1-F3, F3-F7, F7-Fp1, F3-C3) and for the posterior regions (O1-T5, O1-P3, P3-T5, C3-P3), and considered their median value, as in the main analysis.*

*Concerning spatial inter-hemispheric contrasts (contralateral vs disconnected hemisphere) after surgery, Slow Delta Power was larger in the disconnected hemisphere than in the contralateral hemisphere, within the posterior region (T(6)=-3.201, p= 0.0186), and within the anterior region  (T(6)=-3.267, p= 0.0171) (****Supplementary Figure 16****)*

*Concerning temporal contrasts (Post-Pre surgery) of the disconnected hemisphere, Slow Delta Power increased post-surgery with respect to pre-surgery, within the posterior region (T(6)=2.526, p= 0.0449), but not within the anterior region, which only showed a marginal trend (T(6)=1.937, p= 0.1008). (****Supplementary Figure 16****)*

**Supplementary Figure 16. *Regional analysis of Slow Delta Power (0.5–2 Hz) following whole-hemisphere hemispherotomy revealed unihemispheric slowing after surgery, across anterior and posterior regions.*** *Boxplots show Slow Delta Power values of hemispherotomy patients for the contralateral and disconnected cortex, before (left-panel) and after surgery (middle panel), along with the temporal post-pre difference following surgery (right panel). Analyses were restricted to patients undergoing complete hemispherotomy (N=7) to enable separate analysis for the posterior and anterior regions. Each dot represents an individual patient; patients were connected by dashed lines across conditions. (A) Anterior region. After surgery, Slow Delta Power was larger in the disconnected hemisphere compared to the contralateral hemisphere (T(6) = –3.267, p = 0.0171); the temporal increase (Post vs. Pre) in the disconnected hemisphere revealed only a marginal trend (T(6) = 1.937, p = 0.1008). (B) Posterior region. After surgery, Slow Delta Power was significantly larger in the disconnected hemisphere compared to the contralateral hemisphere (T(6) = –3.201, p = 0.0186), and also increased significantly from pre- to post-surgery within the disconnected hemisphere (T(6) = 2.526, p = 0.0449).*

*For consistency, we also performed the same regional analysis for the spectral exponent.*

*Concerning spatial inter-hemispheric contrasts (contralateral vs disconnected hemisphere) after surgery, the Spectral Exponent (0.5-20 Hz) was more negative in the disconnected hemisphere than in the contralateral hemisphere, within the posterior region (T(6)=9.172, p= 0.0001), and within the anterior region  (T(6)=6.402, p= 0.0007). Concerning temporal contrasts (Post-Pre surgery) of the disconnected hemisphere, the Spectral Exponent (0.5-20 Hz) became more negative post-surgery with respect to pre-surgery, within the posterior region (T(6)=6.706, p= 0.0005), and within the anterior region (T(6)=4.61, p= 0.0037).*

*Overall, for slow-delta power, both posterior and anterior regions showed a significant inter-hemispheric asymmetry after hemispherotomy of a whole-hemisphere; there was a pre-post change in the disconnected cortex that was significant only within the posterior region. Further, for the spectral exponent, posterior and anterior regions alike displayed both a significant inter-hemispheric asymmetry after hemispherotomy of a whole-hemisphere, and a significant pre-post significant change in the disconnected cortex. Thus, larger and more consistent effects across regions were observed with the spectral exponent than with slow delta power.
We reason that EEG measures derived from the amount of absolute power (such as slow-delta power) are more affected by non-specific inter-individual variability than measures based on the relative distribution of power (such as the spectral exponent). This variability likely reflects differences in signal amplitude due to pathological heterogeneity (e.g. extent and localization of the epileptogenic zone, cortical atrophy) and surgical factors (e.g. individualized surgical approaches), which influence the amount of preserved gray matter and local conductivity. In contrast, relative measures—by capturing differences in spectral shape—retain inter-individual variability more closely associated with neurophysiological dynamics, while being less sensitive to confounds related to gray matter size and conductivity.*

Further, we performed a regional analysis on slow wave properties to assess potential poster-anterior specificities when comparing the disconnected cortex to reference pediatric sample during wake and NREM sleep (**Supplementary Figure 17, top panels, A,C,E,G**), and applied an age-matching approach to control for the effect of age in each region (**Supplementary Figure 17, bottom panels, B,D,F,H**).

***S13.1 Postero-Anterior specificities of Slow-Delta Amplitude***
*We explored potential regional specificities in the slow wave properties within the disconnected cortex, while accounting for age-specific effects. Thus, we performed an analysis of slow wave amplitude and period, separately in the anterior and posterior regions, in comparison to the reference dataset (****Supplementary Figure 17, top panels,  A,C,E,G****), and applied an age-matching approach to control for the effect of age in each region (****Supplementary Figure 17, bottom panels, B,D,F,H****). In order to consider such regional differences, we restricted the analysis to whole-hemisphere hemispherotomy (N=7), thereby excluding those with a hemispherotomy of the parieto-occipito-temporal area (N=3), and considered median values over electrodes placed in an anterior region (Fp1-F3, F3-F7, F7-Fp1, F3-C3) and in a posterior region (O1-T5, O1-P3, P3-T5, C3-P3), for both the patient and the reference dataset.*

**Supplementary Figure 17. *Regional age-matched analysis of amplitude and period of slow-delta oscillations following whole-hemisphere hemispherotomy (N=7) in relation to the pediatric reference dataset across wake and NREM sleep****.*

*(A–D) Slow-delta envelope amplitude comparison between the patient and reference datasets. (A,C) Boxplots show amplitude values for the disconnected cortex compared to the reference pediatric dataset during wakefulness and NREM sleep, separately for anterior (A) and posterior (C) regions. (B,D) Corresponding pairwise differences relative to wakefulness and NREM sleep, after age-matching. (E–H) Mean period of slow-delta oscillations comparison between the patient and reference datasets. (E,G) Boxplots show period values for the disconnected cortex compared to the reference dataset during wakefulness and NREM sleep, separately for anterior (E) and posterior (G) regions. (F,H) Corresponding pairwise differences after age-matching.*

*Consistently with the main findings, both anterior and posterior disconnected regions showed intermediate amplitude values between wakefulness and NREM sleep; extending the main findings this analysis revealed that the anterior region more closely resembled NREM sleep reference values. Consistently with the main findings, both anterior and posterior regions exhibited slow-delta periods substantially longer than those observed during wakefulness and NREM sleep.*

*Overall, the amplitude of the slow-delta envelope of the disconnected cortex showed intermediate values between those of wakefulness and NREM (N2 and N3 jointly considered), for both anterior and posterior regions (****Supplementary Figure 17  A, C)****, in line with the main results obtained considering the median values over the whole disconnected cortex (****Figure 4 A****) .
Specifically, age-matching revealed that the anterior disconnected cortex showed amplitude values significantly larger than those of wakefulness (T(6)=2.939, p = 0.026, with 7/7 subjects showing a consistent pairwise difference) and not significantly different from those of NREM sleep (T(9)=-0.764, p = 0.463, with 5/7 subjects showing a consistent pairwise difference),* ***Supplementary Figure 17 B.***  *Moreover, age-matching revealed that the posterior disconnected cortex showed larger amplitude values than those of wakefulness, albeit only with a non significant trend (T(6)=2.206, p = 0.0695, with 6/7 subjects showing a consistent pairwise difference), and significantly smaller than those of NREM sleep (T(6)=-0.764, p = 0.463, with 6/7 subjects showing a consistent pairwise difference),* ***Supplementary Figure 17 D.***

***S13.2 Postero-Anterior specificities of Slow-Delta Period***

*Further, the period of slow-delta oscillations of the disconnected cortex showed values higher than those of both wakefulness and NREM, for both anterior and posterior regions (****Supplementary Figure 17 E, G****), in line with the main results obtained considering the median values over the whole disconnected cortex (****Figure 4B****).*

*Specifically, age-matching revealed that the anterior disconnected cortex showed period values significantly larger than those of both wakefulness (T(6)=3.7, p = 0.01, with 7/7 subjects showing a consistent pairwise difference) and NREM sleep (T(6)=3.349, p = 0.0154, with 7/7 subjects showing a consistent pairwise difference),* ***Supplementary Figure 17 F.***

*Similarly, age-matching revealed that the posterior disconnected cortex showed period values significantly larger than those of both wakefulness (T(6)=5.363, p = 0.0017, with 7/7 subjects showing a consistent pairwise difference) and NREM sleep (T(6)=3.494, p = 0.0129, with 7/7 subjects showing a consistent pairwise difference),* ***Supplementary Figure 17 H.***

*In sum, while both anterior and posterior regions of the disconnected cortex showed values of the amplitude of the slow-delta envelope intermediate between wakefulness and NREM sleep, age-matching revealed that the anterior region showed values that resembled NREM sleep reference values more closely than the posterior region. Moreover, both anterior and posterior regions of the disconnected cortex showed values of the period of slow-delta oscillations that substantially exceeded those of both wakefulness and NREM sleep, in both the anterior and posterior regions. This age-matched regional analysis expanded the analysis presented in* ***Figure 4*** *(Mean amplitude of the slow-delta envelope and period of slow-delta oscillations in the disconnected hemisphere, compared with respect to wakefulness and NREM sleep in the reference pediatric sample), revealing postero-anterior specificities in slow wave amplitude within the disconnected cortex, that could not be ascribed to an age-related effect.*

*We reason that such postero-anterior differences in the amplitude of the slow-delta envelope may be related to the specific structural patterns—particularly the location and extent of the lesions. In particular, post-ischemic damage may contribute to a reduced EEG signal amplitude.*

**Specific/minor comments**

3. It would be important to describe briefly paroxysmal activities in the isolated cortex, in particular, because such activities were previously described in human isolated cortex (Echlin FA, Arnet V, Zoll J. Paroxysmal high voltage discharges from isolated and partially isolated human and animal cerebral cortex. Electroencephalography and Clinical Neurophsysiology. 1952; 4: 147-164).

>> We thank the reviewer for raising this important point. While the focus of the main analysis was on background EEG activity, and we carefully excluded segments with epileptiform activity, we believe that it can be valuable to add supplementary description of this activity.
It is interesting to note that the epileptic activity recorded in the isolated cortex appears to be strongly dependent on the nature of the isolated tissue. Specifically, our sample consists of either patients with extensive cortical malformations (mainly cortical dysplasias and polymicrogyria) or patients with post-stroke sequelae, predominantly characterized by malacic and gliotic tissue resulting from a pre- or perinatal stroke.

In the first group, we observe the persistence of frequent epileptiform discharges, such as spike/polyspike-wave complexes or sharp wave-slow wave complexes with high voltage. In the second group, occasional epileptic abnormalities like isolated spikes or sharp waves are seen, occurring against a background of low-to-medium voltage activity. This difference is visible in **Supplementary Figure 1**, where we show the epochs included and excluded from the analysis.

In both cases, however, the epileptiform discharges seem to organize into bursts, interspersed with epochs where the background activity predominantly consists of slow waves.

This descriptive finding suggests that an isolated area of malformed cortical tissue is capable of intrinsically generating epileptic activity. Conversely, post-ischemic tissue, when isolated, tends to generate sporadic and isolated discharges, which were significantly increased in frequency and spread when connections with subcortical structures and the contralateral hemisphere were intact.

We have briefly described epileptic activity in the **Supplementary Material, section S1.**

*Epileptiform activity in the isolated cortex was found to be dependent on the nature of the underlying tissue. In patients with extensive cortical malformations (such as cortical dysplasias and polymicrogyria), frequent high-amplitude epileptiform discharges, including spike/polyspike-wave complexes and sharp wave-slow wave complexes were observed. In contrast, in patients with post-stroke sequelae, isolated spikes or sharp waves occurred against a background of low-to-medium voltage activity. In both cases, epileptiform discharges are organized into bursts separated by periods of slow wave activity (see* ***Supplementary Figure 1*** *for examples).*

4. In multiple figures it is unclear which state is reported. Sometimes it is very confusing. in Fig. 3 legend you state 'The contralateral cortex, both before and after surgery, showed values compatible with wakefulness'. Were patients awake or they were asleep but values of spectral exponent were compatible with wakefulness.

>> We acknowledge that the manuscript would benefit from several reminders that the hemispherotomy patients were always behaviorally awake. We have accordingly made several mentions of it (Please see answer to **comment 1**).

**5.**	p. 17 of pdf …reached all NREM sleep stages (up to N3),… What classification of states was used? In (Iber C, Ancoli-Israel S, Chesson A, Quan S. Manual for the Scoring of Sleep and Associated Events: Rules, Terminology and Technical Specifications. Westchester: American Academy of Sleep Medicine; 2007) there is stage 1, stage 2 and SWS. Stage 3 was used in earlier classifications.

**>>** The classification of sleep stages in our study follows the standard criteria introduced by the American Academy of Sleep Medicine (AASM) in the 2007 manual edited by Iber et al., which redefined non-REM stages as N1, N2, and N3—combining the earlier stages 3 and 4 into N3, formerly known as SWS. This terminology remains in use in later editions of the AASM scoring manual, including those edited by Berry (2018) and Troester (2023). Thus, our reference to N3 corresponds to the current standard for deep NREM sleep.

Berry RB, Albertario CL, Harding SM, et al. The AASM Manual for the Scoring of Sleep and Associated Events: Rules, Terminology and Technical Specifications. Version 2.5. Darien, IL: American Academy of Sleep Medicine; 2018.

Troester MM, Quan SF, Berry RB. The AASM Manual for the Scoring of Sleep and Associated Events, Version 3. United States: American Academy of Sleep Medicine; 2023.

**6.**	p. 17 of pdf. …virtual EEG reference… unclear to me what is "virtual EEG reference"? Please explain.

>> We appreciate the reviewer’s input, which gives us the opportunity to better express this point that was previously expressed unclearly. In both the patient and reference datasets, EEG was originally recorded using a physical reference electrode, enabling the measurement of voltage differences between electrodes. However, once the EEG signals are digitized, the referencing scheme (or montage) can be modified retrospectively through simple linear operations—commonly referred to as re-referencing. In the reference dataset, we initially used a common average reference (as in Favaro et al., 2023). In contrast, for the patient dataset, we employed bipolar derivations, also computed via digital re-referencing, to avoid smearing large voltage fluctuations between hemispheres.

To allow direct comparison between groups, we reprocessed the reference dataset using the same bipolar montage applied to the patient data—effectively aligning the virtual references. In light of the reviewer’s comment, we entirely rephrased the sentence for greater clarity.

*Furthermore, we digitally re-referenced the EEG recordings of the pediatric reference dataset to match the bipolar montage used in the patient dataset, ensuring methodological consistency for comparative analyses (see section Data acquisition and Preprocessing).
~~Finally we adapted the virtual EEG reference of the pediatric reference dataset to that of the patient dataset, which required relatively high spatial specificity.~~*

**7.**	The calculation of Amplitude of Slow-Delta activity is unclear to me. Could you please make a panel in Suppl. Fig. 6 to explain this graphically?

>> Yes, absolutely; we can visually exemplify the computation of the amplitude of Slow-Delta activity graphically. The procedure is fairly simple and detailed in a new dedicated **Supplementary Figure 12**. Hereby we employed a randomly chosen segment of 30 seconds of the isolated cortex from one hemispherotomy patient.

***Supplementary Figure 12.*** *The EEG signal (cyan trace) is first band-pass filtered in the slow-delta band (0.5–2 Hz; black trace). The envelope of the oscillation is then computed as the absolute value of the Hilbert transform of the filtered signal (red trace). This represents the instantaneous amplitude, calculated as the vector length in the complex plane, combining the real and imaginary components of the oscillatory dynamics (Cohen MX, 2014). Finally, the average value over time is retained for subsequent statistical analyses.*

*Cohen MX. Analyzing Neural Time Series Data: Theory and Practice. Cambridge, MA: MIT Press; 2014. doi:10.7551/mitpress/9609.001.0001*

**8.**	In the section entitled 'Period of slow delta oscillations and rate of large slow waves' I did not find how you defined and detected 'large slow waves'?

>> We thank the reviewer for pointing out an outdated residual mention from an earlier draft,  which was ultimately excluded during the internal revision of the manuscript. Specifically, a previous draft included an analysis of slow wave rate, which yielded results largely consistent with the current findings based on slow wave amplitude (see above). However, this measure was removed as it was redundant with current estimates and methodologically less straightforward, particularly due to the difficulty of defining an appropriate amplitude threshold across both the disconnected cortex and the cortex of neurotypical controls. This analysis  became superfluous in the light of the reported amplitude differences. We have now removed the remaining reference to this earlier analysis from the title to ensure consistency.

**9.**	Please be consistent with terminology/wording. In the section 'Sigma oscillations and spindle activity' you talk about low amplitude alpha activity, but no word about sigma! In the main text, regarding spindle detection you write you relayed on a trained neurologist, which is not reliable to me for such a fine study, but in the supplemental materials, you describe a reliable automatic method of spindle detection.

>> We thank the reviewer for these valuable comments, which are part of a suggested broader revision of the analyses related to spindle activity. We decided to move the detailed treatment of these analyses to the Supplementary Materials (section **S14**, ***Sigma oscillations and spindle activity***). Please see also the answer to **comment 1.1**. As indicated, we emphasized the multiple assessments used to investigate spindle activity and did not rely solely on visual inspection by a trained neurologist. In the Supplementary Materials, we clarified in the first paragraph of the dedicated section the sequential process that led from an initial qualitative inspection of EEG traces to more fine-grained quantitative analyses. It is important to note that on qualitative inspection, post-disconnection EEG traces did not display full-fledged spindles but rather short stretches of low-voltage activity in the alpha band, not in the sigma range—a finding that was subsequently confirmed through quantitative analysis.

*“To determine whether the disconnected cortex (recorded when the patients were behaviourally awake) was devoid of spindle oscillations, a trained neurologist first inspected the EEG traces. Despite the abundance of slow oscillations resembling a sleep-like pattern, spindle grapho-elements were not identified and only sporadic, low-amplitude alpha (and not sigma) activity could be observed in the disconnected cortex, in line with the established thalamic origin of sleep spindles (Luthi, 2014; Fernandez and Luthi, 2020). Subsequently, quantitative spectral and temporal analyses were performed to confirm this qualitative finding.”*

**10**.	The first paragraph of the method section and the first paragraph of the result section largely repetitive.
>> We thank the reviewer for the comment. While there is some overlap between the first paragraphs of the Methods and Results sections, their focus serves distinct purposes. The Methods paragraph was designed to specify the criteria and procedures used to retrospectively select the patient cohort, ensuring methodological transparency and reproducibility. In contrast, the first paragraph of the Results section provides a factual summary of the actual characteristics of the selected cohort, including etiology, surgical procedures, timing of recordings, duration of EEG recordings, as part of presenting the study outcomes. We believe maintaining this structure improves clarity for the reader by separating the methodological requirements from the actual characteristics of the resulting sample.

**11.1**	Fig. 1. It is my understanding that all the data in this fig are coming from a single subject. It would be nice to indicate this in the title of the fig.

>> We thank the reviewer for the suggestion. As correctly noted, Figure 1 presents data from a single subject. In response, we have specified this directly in the figure title, rather than only in the legend, for greater clarity:

*~~Representative~~* ***Narrow- and broad-band spectral changes after hemispherotomy, in an awake representative patient, displaying marked EEG slowing of the isolated cortex.***

**11.2**	Fig. 1B. I presume that recordings in the right and left hemispheres (pre and post) are done simultaneously. It is not explicitly stated.

>>We thank the reviewer for the observation. As correctly presumed, EEG recordings in these patients are always performed across the entire scalp, simultaneously capturing electrical activity from both hemispheres. We have specified this detail in the figure caption as follows:

*‘(****B****) EEG recordings were performed simultaneously for both hemispheres 8 months before surgery (at 11 y.o.), and 12 months after.’*

**12.**	Fig. 1 C and D. Several things are unclear:
What was the state NREM sleep (stage?), REM sleep or wake?

>> We thank the reviewer for the observation. As correctly presumed, EEG recordings were performed simultaneously across the entire scalp, capturing activity from both hemispheres. We have now explicitly clarified this point in both the figure title and in the caption as follows:

***‘Narrow- and broad-band spectral changes in an awake representative patient after left hemispherotomy displaying marked EEG slowing of the isolated cortex.***

*Cortical isolation of the left hemisphere in a 11 y.o. representative patient during wakefulness.(..) (B) EEG recordings were performed during wakefulness, simultaneously for both hemispheres.*

**13.**	What was the duration of the analyzed and shown recording?
>>In Figure 1, the patient's EEG was displayed for illustrative purposes over a short segment of 6 seconds, as reported in the legend, whereas the duration of the recording after exclusion of epileptiform activity was of 234 seconds (~4 minutes) before surgery and 426 seconds (~7 minutes) after surgery, which was used to compute the PSD (**Fig 1 C**). We accordingly add this information in the Figure legend:

*Short EEG segments (6 s) from the isolated cortex (F7-F3 and T5-P3 bipolar derivations, left panel) and the contralateral homologue (F8-F4 and T6-P4, right panel) are shown, pre-surgery (above) and post-surgery (below), selected from the recordings (respectively, 4 and 7 minutes, after the exclusion of artifactual and epileptiform segments).*

**14**.	Fig. 3 and supplementary fig. 5 upper part looks to be identical.  Such a repetition is not needed. However, it would be important to see in patient data set dynamics of slow/delta power and or spectral exponent throughout states: W, N1, N2 and SWS before and after disconnection, something like what you have shown for the reference dataset.
>> We apologize for not making clear enough the patient’s vigilance state: all recordings available were performed during wakefulness, vigilance changed only in the reference dataset. Future studies should acquire EEG recordings in patients with hemispherotomy during both wakefulness and sleep, to assess whether vigilance-related fluctuations influence the state of the disconnected cortex via systemic factors such as hormones, neuromodulators, or perfusion.

We accordingly mention in the **Limitations and future directions** section:

*Future prospective studies should include sleep recordings and repeated EEG assessment, to assess potential state-dependent changes and their evolution over time.*

The bootstrapped differences analysis is going to this point, but it looks a bit confusing to me. Either it shows something that I do not understand, or the description is not adequate. Ideally, you should present real measurements.

>>To clarify this last point, the mentioned bootstrap analysis estimates the differences between the means of conditions estimated across bootstrap resamplings. Let’s consider a single comparison of spectral exponent values, for instance, that of the values from the disconnected cortex in awake hemispherotomy patients and that of N2 sleep in the reference dataset. For each bootstrap resampling iteration, the mean of the disconnected cortex and the mean of N2 in the reference dataset is estimated, and the difference of such means is computed. Across iterations, this led to a distribution of difference of means, thereby giving an idea of how distant are the means of the two populations, according to bootstrap methodology.

**15.**	Suppl Figs 6 and 7 please increase font size. It is almost impossible to read with the current font size.

- Fig. 5. Please increase font size. I cannot read any numbers in the insets even after magnification.

>>We have accordingly increased font size in the mentioned Supplementary Figures

- Fig. 2 B-E Square symbols and dots are too small to be visible.

>> To better visualize different subgroups among hemispherotomy patients (including those marked by squares and dots, related to the type of hemispherotomy), we have created a new dedicated Supplementary Figure

***Supplementary Figure 7 Etiology and Type E. Narrow- and broad-band spectral changes after surgery reveal a marked EEG slowing of the isolated cortex, robust across etiology and type of hemispherotomy***

*This figure mirrors* ***Figure 2*** *from the main text, graphically highlighting the different patients in terms of etiology and type of disconnection. Specifically, patients who underwent complete hemispherotomy are marked with a circle, while those who underwent temporo-parieto-occipital disconnection are marked with a square. Additionally, patients with a cortical developmental malformation as the underlying etiology are shown in red, whereas those with pre- or perinatal stroke sequelae are shown in green.* ***A****) Slow Delta Power (0.5-2 Hz) was significantly larger in the disconnected than in the contralateral cortex only in the session after surgery (****Figure 2A****). This pattern was observed in 4/4 patients with a cortical malformation, 5/6 patients with pre/perinatal ischemic stroke, 3/3 patients with temporo-parieto-occipital disconnection, 6/7 patients with hemispherotomy* ***B)*** *Slow Delta Power increased significantly pre to post surgery only in the disconnected cortex (****Figure 2B****); this pattern was observed in the same subset of patients described above, across the subgroups of etiology and type of hemispherotomy.*

***C)*** *The spectral exponent (0.5-20 Hz) was significantly more negative in the disconnected than in the contralateral cortex in the session after surgery* ***(Figure 2C).*** *This pattern was observed in all conditions: in 4/4 patients with a cortical malformation, 6/6 patients with pre/perinatal ischemic stroke, 3/3 patients with temporo-parieto-occipital disconnection and 7/7 patients with hemispherotomy.* ***D)*** *The PSD became significantly steeper from pre to post surgery only in the disconnected cortex, as indexed by the spectral exponent (****Figure 2D)****; this pattern was observed in all the patients, across the subgroups of etiology and type of hemispherotomy.*

Briefly, all subgroups related to etiology and hemispherotomy type revealed a coherent pattern to that observed across all patients (**Fig. 2**).

**16.**	Fig. 4, Suppl Fig 7 and related text. You mention here slow/delta waves during wake in contralateral cortex. The presence of slow waves during wake (unless patients were sleep deprived) is a big surprise to me. Please illustrate with original recordings the slow/delta waves presence during wake.

>> We thank the reviewer for the comment, which allows us to clarify that we do not intend to suggest the presence of classical slow waves during wakefulness in the contralateral cortex, rather, we aimed to contextualize quantitative features of slow wave activity of the disconnected cortex.

Let’s consider first that the EEG has a rich broad-band quasi-rhythmic and arrhythmic activity, with a larger prevalence of low frequencies–especially in children–according to an approximate 1/f distribution. Hence, a low frequency signal component can be obtained using a filtering approach, from which quantitative features can be estimated, assessing period and amplitude. We extracted such features from slow-delta oscillations in the disconnected cortex, and then provided a contextual reference to interpret such features—primarily considering the neurotypical pediatric dataset during NREM sleep and its baseline wakefulness (**Figure 4**), and moreover, to thoroughly display the distribution of values obtained within the patient and in the reference dataset (**Supplementary Figure 13 and 14** ).

To compare the period of the slow-delta activity, we processed the signals in the same way across both the patient and reference dataset, by filtering the EEG in the delta band (0–4 Hz) and selecting only those oscillations with a period longer than 0.5 seconds. We did not perform a classical slow wave detection due to the montage available in our recordings (bipolar derivations and absence of mastoid electrodes), and, further, to avoid setting an arbitrary amplitude threshold differently impacting neurotypical reference subjects and hemispherotomy patients (given the known impact of the gray matter density and the lack of synchronizing inputs from the thalamus).

In the contralateral cortex, when patients were awake, this approach isolated a slow-activity component, characterized by low-to-medium amplitude oscillations with a long period above 0.5 seconds, derived from background polyrhythmic activity characteristic of infants and children—especially if there are brief “intrusions of drowsiness”. This activity is clearly distinct, in both amplitude and period, from the slow wave activity detected during wakefulness in the disconnected cortex.

**17.**	Fig. 5 and related text. I have again a problem with your data presentation. For your reference group all is clear. During sleep there is a maximum around 13 Hz, which likely represent spindles. In your patient group, the state of vigilance is not indicated and in contralateral as well as in pathological hemisphere, there is no sign of a peak around 13 Hz. The peak is around 9 Hz, likely alpha. This indicates to me that the recordings were collected during wake.  So how did you expect to record spindles during wake? Why do you make a section of your paper about spindles in a condition in which spindles cannot be observed? Please make sure that you show data separately for NREM sleep and wake.
**>>** As correctly pointed out by the reviewer, the analysis was confirmatory: we did not expect to observe spindles, rather we wanted to show that their absence was probably the most obvious and striking difference from NREM sleep. On a second thought, we agree that this analysis is not central to the paper, and is thereby moved to the supplementary material. Accordingly, we replaced these lengthy sections of the paper with short mentions in the main text (see **comment 1.1**)

**18.**	Suppl Figs 6 and 7 has panels identical to the panels shown in the fig 4.

Fig. 4 is not mentioned in the result section.

>> We appreciate the attention to details of the reviewer for this helpful observation. We have now addressed both issues to mention the correspondences. First, we added a direct reference to **Figure 4** in the Results section, where the analysis is first introduced:

*We further characterized slow wave activity occurring during wakefulness in the disconnected cortex, by comparing the average amplitude of the envelope of EEG activity filtered in the slow-delta band with respect to the pediatric reference sample across both wake and sleep (****Figure 4****).*

Second, we clarified the relationship between the main and supplementary figures by explicitly indicating, in the legends of **Supplementary Figures 13 and 14**, which panels correspond to those in **Figure 4** (i.e. ‘***as in Figure 4, left panel’, ‘as in Figure 4, right panel***). These supplementary figures are now also cross-referenced in the main figure legend to guide the reader to additional detail (*See* ***Supplementary Figure 13 and 14*** *for additional details)*

**19.**	p. 29 of pdf. '… longer periods compared to that seen during physiological sleep (Figure 5).' You **likely mean Fig. 4.** Fig. 5 is about spindles.

>> Thank you for noting this incorrect reference, which was meant to point to the former Figure 4.
p. 30 of pdf '…consistent with the characteristics of physiological wakefulness (Figure 4,…' Do you mean Fig. 5 or some supplemental figs?

>> Thank you for noting this incorrect reference, which was meant to point to the former Figure 5. This figure has now been moved to the supplementary material, and renamed as **Supplementary Figure 18**

**20.**	p. 30 of pdf '…interesting questions about the role of slow waves after disconnection.' It seems that there is **no role of slow waves in isolated cortex**. As you mentioned above, slow wave activity in isolated cortex **is default state.**

>> According to the reviewer’s suggestion, we have removed such sentence from the Discussion.

*~~This result is unsurprising, as spindles are generated in the thalamus and require intact bidirectional thalamo-cortical connectivity (David et al., 2013), but raises interesting questions about the role of slow waves after disconnection.~~*

We also revised the following discussion paragraph (moving it from the end of the **‘Limitations and future directions’** to the end of the revised paragraph ‘**Properties of post-disconnection slow waves’**), changing the order of the arguments, to place greater emphasis on slow waves as the default mode of cortical electrical activity. We reinforce the considerations made from Krone and Vyazovskiy (2021) by also referring to the experimental study from Lemieux et al. (2014) and the review paper from Sanchez-Vives et al. (2017).

*Our discovery of slow waves persisting for years in the human isolated cortex raises significant questions. Specifically, the functional significance of cortical slow waves intruding during wakefulness—in absence of the subcortical inputs required for physiological sleep regulation and spindle activity—remains unclear. Given that similar sleep-like dynamics are commonly observed following a wide range of brain injuries (Massimini et al., 2024), it will be important to determine whether these patterns retain neuroplastic or homeostatic functions (Carmichael and Chesselet, 2002; Tononi and Cirelli, 2006; Facchin et al., 2020; Sarasso et al., 2024; Tscherpel et al., 2024) or whether they merely represent a regression of cortical activity to its default ‘intrinsic’ state (Lemieux et al., 2014; Sanchez-Vives et al., 2017; Krone and Vyazovskiy, 2021). Addressing this question is particularly important for understanding the role of slow waves after brain injury and their potential implications for rehabilitation, and will require focused longitudinal investigations.*

Finally, we conclude the manuscript by returning to this theme in the new **conclusions** section, noting that the long-term persistence of slow waves raises the fundamental question of whether they retain any functional role or simply reflect a reversion to an intrinsic default cortical mode.

*The persistence of slow waves, lasting months to years after surgery,  raises the question of whether they play any functional role or merely reflect a regression to a default mode of cortical activity.*
