# Supplementary material for: Hemispherotomy leads to persistent sleep-like slow waves in the isolated cortex of awake humans
Source: PLoS Biol. 2025 Oct 16;23(10):e3003060. doi: 10.1371/journal.pbio.3003060 (PMC12530565; doi:10.1371/journal.pbio.3003060)
Supplement: Attachment — Submitted filename: response to reviewers_2nd rebuttal.docx [file pbio.3003060.s003.docx]

**Reviewer #1** (Bjørn Erik Juel signed his report): First of all, I would like to commend the authors on a tremendous effort in the revision process. Despite the largely positive reviewer feedback, the authors went above and beyond what I would typically expect in replying to my previous comments. In other words, I am satisfied with their effort, and accept almost all edits proposed in the resubmission.

We are grateful for the positive remark and for the stimulating and insightful comments that this referee has provided throughout the revision process, which resulted in a very engaging and constructive exchange.

There is, however, one set of responses from the authors (with associated changes in the resubmission) that I cannot leave unanswered. It is most prominent, I think, in the response to point 5 (to reviewer 1). They propose the following sentence: "The findings of prominent slow oscillations and broad-band slowing support inferences of **absent or reduced awareness** in the isolated cortex." However it also shines through in other responses and edits, like "In sum, our findings of a unihemispheric electrophysiological spectral profile resembling deep-sleep are **consistent with absent, or reduced conscious** experiences, **rather than constituting definitive evidence against** any form of conscious experience" in combination with "It remains possible that **some form of consciousness could persist** even in the presence of highly prominent slow waves in the spontaneous scalp EEG, as revealed by peculiar genetic conditions, i.e. Angelman syndrome (Frohlich et al., 2021), and by sometransient, disconnected psychedelic states (Blackburne et al., 2024). More broadly, growing evidence suggests that some form of disconnected, residual consciousness may persist in states traditionally labeled as "unconscious"—such as deep sedation, NREM sleep, and the vegetative state."

While I am well aware of the long standing tradition of labeling states such as nrem sleep and deep sedation as states of unconsciousness or absence of experience (or the weaker, but similar, "reduced consciousness"), it is just not the case that such states are typically devoid of experience, or even necessarily associated with something that is reasonably labeled with the diminutive "residual consciousness". To the contrary, **reports of rich experiences are not that uncommon** (from both NREM and propofol sedation (RASS -4/MOAAS 1), and combined with the well known amnesic effects of anesthetics, and the confused and tired state of individuals trying to report their experiences upon awakening from deep nrem sleep or sedation, it seems more reasonable to err on the side of caution in these situations. Of course, I understand that it is sometimes necessary to provide simple stories and narratives (e.g. in clinical situations where next-of-kin might need comfort), but an academic article is not the place for it.

As remarked in our letter to the editor, we are very sympathetic with the reviewer’s position and we acknowledge the evidence that NREM sleep, deep sedation and the vegetative state are not necessarily states of diminished or absent consciousness, as indeed a significant amount of rich conscious reports can be obtained in this conditions, in at least some people, by careful probing procedures. We have now carefully gone through the text and removed any general statement, explicitly or implicitly, implying this simplistic assumption.
To highlight this shared perspective, in the revised discussion, we now explicitly mention the empirical observations from serial awakenings in NREM sleep.

*‘serial awakenings (in deep NREM sleep) during full nights of sleep, result in a significant rate of dream reports (12-25%, 45%, 34% according respectively to Nielsen, 2001; Siclari et al., 2013; Siclari et al., 2018), reports of vague dreams without content (50%, 33%, 39%) as well as reports of no conscious experience (50%, 22%, 28%).’*

To deal with this, I recommend something like the following:

- In place of phrases like "absent or reduced awareness**" substitute "conscious experiences similar to those typical of NREM sleep and deep sedation**". This maintains the possibility that the disconnected hemisphere has no, reduced, residual, non-rich experiences (depending on what is actually the case in NREM etc), without drawing assumptions that have a high likelihood of being too simplistic, given the current state of the evidence.

- Alternatively, make it even more clear that the current evidence is, contrary to the traditional view, that NREM sleep, deep sedation, and other states with pronounced slow waves are generally not devoid of conscious experience (despite the problems associated with reporting such experiences), but that **the authors nevertheless choose to assume** that they are in fact best characterized as **states with "absent or reduced awareness**".

As discussed above, we do not endorse the general statement of the second option and we now better qualify our position; slow waves can be of different types with differential impact on dream reports. We now explicitly mention within-state comparisons during NREM sleep showing that absence of reported conscious experience is associated with lower spindle density, higher low-frequency power, together with an increased prevalence of a distinct type of slow waves—small, shallow, frequent waves lacking accompanying high-frequency bursts—generated from cortico-cortical interactions (Siclari et al., 2018). These electrophysiological features and type of slow waves closely resemble those observed in the disconnected hemisphere, which is structurally devoid of subcortical inputs. We have revised the relevant sentences in the Discussion to better reflect this interpretive nuance, that support our provisional and non-exclusive inferences that the disconnected hemisphere may sit in a state of absent or diminished awareness. We also acknowledge in the discussion and in the limitations section that different interpretations remain plausible, and help devise future experiments to narrow down the current ambiguities.

In this revision, we have taken further steps to clarify that our claims are interpretive rather than definitive. Moreover, we have shifted the argumentative weight in the Discussion from general analogies with NREM or sedation toward within-state evidence and specific electrophysiological dynamics, in order to support provisional inferences about consciousness.

Finally, I would recommend removing speculation/inferences about conscious state/contents of the disconnected hemisphere from highlights and abstract and leave it purely to the discussion as interpretation of the results (with a clear indication that inferences about absent/reduced/un-consciousness are based on common but empirically disputed assumptions regarding the ground truth of consciousness in nrem/deep sedation/UWS/etc).

>We have also revised the Abstract to reflect the interpretive nature of our conclusions. Specifically, we now state that the findings **“provisionally support”** inferences of absent or reduced awareness in the disconnected hemisphere. The abstract now juxtaposes the robustness of the observed neurophysiological alterations with a clear acknowledgment that our inferences are limited and not definitive.

Something similar, I think, should be the case for the end of the introduction: avoid making too strong assumptions about what slow-waves allow you to infer about (un)consciousness. Despite the authors claiming (with many references!) that the measures used is "validated as a reliable EEG marker of the level of consciousness across a range of physiological [...], pharmacological [...], and pathological states", it is, unfortunately, the case that the ground truth about absence of (or reduced) consciousness was rarely adequately controlled for in the tested populations/cited studies, and thus the success of the marker (and most others with it) relies on the traditional (but too simplistic) preconceived assumptions about presence/level of consciousness in those populations. (Im not saying there are other markers that fare any better, just that most studies to date of testing/validating markers of consciousness have taken for granted the traditional view of consciousness in nrem/sedation/uws/etc)

>Regarding the comment on the Introduction, we respectfully note that the sentence in question does not rely on an explicit binary assumption about the presence or absence of consciousness. Rather, it appears right after a paragraph that emphasizes the limitations of slow-wave-based markers, and acknowledges growing evidence for **conscious experience during states traditionally labeled as unconscious**. In this revision, we have further strengthened that nuance by explicitly adding that **conscious experiences can occur in deep sleep and deep sedation** (Fröhlich et al., 2021; Sevenius Nilsen et al., 2022).
The sentence now reads:

*“While the presence of slow waves may not necessarily indicate a complete loss of consciousness, and conscious experiences can occur in deep sedation and deep sleep (Fröhlich et al., 2021; Sevenius Nilsen et al., 2022), a background EEG dominated by slow-wave activity is broadly recognized as a key factor in clinically and ethically significant decision-making.”*

Our framing reflects a pragmatic and operational perspective, in line with how background EEG activity—particularly slow-wave dominance—is often interpreted in clinical contexts for ethically relevant decisions.

Anyways, I recognize that my (strong) opinions are countercultural and not typical of the traditional view of our field, and therefore I leave it up to the authors how/if they wish to take this points into consideration, and to the editor to make the final decision. In other words, I think the paper can easily be published as is, but I'll just reiterate that I think the paper would be more stringent and less speculative if empirically disputed, but traditionally common, assumptions about absent/reduced consciousness in slow-wave states were edited to be more agnostic about the ground truth.

Congratulations, and best of luck!

We thank the reviewer for his open-mindedness and for engaging with our work in a clear and constructive way. We appreciate the thoughtful articulation of concerns, as well as the fairness in leaving room for different interpretive approaches. We have aimed to reflect this exchange by clarifying the provisional nature of our claims, while preserving the core scientific message.

**Reviewer #2:** the authors have answered all my questions satisfactorily, and I congratulate them on their work.

We thank the reviewer for their positive feedback and appreciation of our work.

## **Reviewer #3** (Igor Timofeev signed his report): overall, the authors provided satisfactory corrections to my critique. Therefore, I have only some minor suggestions.

We thank the reviewer for carefully re-evaluating our revised manuscript and for the positive overall assessment. We are especially grateful for the detailed and precise comments, which allowed us to further improve the clarity and consistency of the manuscript and supplementary materials.

Please check color code in Supplementary Fig. 3. I have feeling that pre and post colors, particularly for disconnect hemisphere are reversed.

Supplementary Fig. 14. In the legend, you reference to panels A, B and C. In the figure itself the panels are not labelled as A, B, C.

We carefully reviewed all figures and corrected minor oversights. In particular, the color code in Supplementary Figure 3 has been checked and remains correct; the mentions in the legend to the panels in Supplementary Figure 14 have been labeled properly (left, middle, right panels). With respect to the colors of Suppl. Fig 3: one can appreciate the results best in comparison with figure 2; specifically, after disconnection, while the disconnected cortex enhances lower frequency activity and steepens its spectral decay, it also displays less deviations from the aperiodic 1/f-like background.

On a side note, It must also be noted that periodic or semi-periodic activity (Palva and Palva 2018) in lower frequencies rarely appears as well-formed distinct peaks over the 1/f-like decay of the PSD, making it difficult to disentangle from the aperiodic EEG background. This remains an underdeveloped area of research. Although some recent biologically inspired models have been proposed, they tend to be overly conservative and fail to reproduce an actual 1/f profile (Brake et al., 2021). Others have pursued different analytical strategies, for instance, empirical mode decomposition (Blackburne et al., 2024).

Brake	N, Duc	F, Rokos	A, Arseneau	F, Shahiri	S, Plourde	G. Aperiodic EEG activity masks the dynamics of neural oscillations during loss of consciousness from propofol. bioRxiv2021 October 12.464109. 2021: 10.1101/2021.10.12.464109.

p. 128 of PDF … with respect to physiological NREM sleep and wakefulness… Here, and in some other places below. It is clear that a major part of the study was done during waking state. Because still some recordings (reference dataset) were done during NREM, and in order to avoid any confusion, please double check that in each part of the manuscript and figure you clearly outline the exact state of the patient.

As suggested, we systematically checked the entire manuscript and figure legends to clarify in which vigilance state the EEG was recorded.

Some references in the text are not found in the list of references. I did not check all, but at least Lemieux et al., 2015 is cited in the text, but could not be found in the list of references.

We thoroughly reviewed both the main text and the supplementary materials to ensure that all cited works are included in the final reference list.

Some supplementary figures are either not referenced in the text or they are not referenced in the order from Supp. Fig. 1 through Supp. Fig. 19. Please check and update.

We revised the text to improve the clarity of figure references, especially in relation to the order in which supplementary figures are presented and cited. The order of citation is primarily introduced in the **Materials and Methods** section, following the logical structure of the analytical pipeline. This explains why, in the **Results**, some figures and supplementary sections may appear out of numerical sequence.
To improve clarity:

- We added explicit references to **Supplementary Figures 4 and 7** in the main text.
- We now directly cite **Supplementary Section S8 and Figures 9, 10, and 11.**
- **Supplementary Figure 12,** which is a methodological clarification, is not cited in the main text but visually complements **Supplementary Section S10** (which is cited).
- We rearranged the supplementary tables and moved the former Table 3 (now **Table 1**) to the beginning of the Supplementary Materials, as it contains the demographic and clinical information and is the first table cited in the main text.
- Finally, we added a few sentences in the **Materials and Methods** to explicitly cite **Supplementary Sections S12 and S13,** which were previously referenced only in the Results, and could thus cause confusion.

Below we report all relevant text additions in their context (*additions are marked by underlined text,* see tracked changes in the revised manuscript and supplementary file).

*Here, we estimated the spectral exponent over the 0.5–20 Hz range, so as to include the Slow Delta range, while excluding higher frequencies which are prone to muscular contamination in children (we showed in Supplementary Figure 4, for completeness, a group-level broad-band PSD estimated over the 0.5-40 Hz range). A similar fitting range, 1-20 Hz, previously used in Favaro et al., 2023, was also investigated in our patients’ sample for completeness (Supplementary Material, section S3.2; Supplementary Figure 5).*

*We further compared the strength of statistical evidence between narrow-band and broad-band spectral features by means of bootstrap analysis (Supplementary Material, section S5; Supplementary Figure 6). Moreover, we evaluated whether etiology and type of hemispherotomy had any influence on the main findings (Supplementary Material, section S6; Supplementary Figure 7).*

*Finally, given the well-known impact of age on EEG features (Kurth et al., 2010; Gorgoni et al., 2020; Favaro et al., 2023), we conducted age-matched supplementary analyses to factor out a potential effect of age in our findings (Supplementary Material, section S8 and S9; Supplementary Figures 9, 10, 11).*

*We then estimated the mean period across all Slow Delta oscillations (Supplementary Material, section S11). A density plot of the typical span of the period values across subjects is shown in Supplementary Figure 14, for both the patient and reference dataset. To compare values between subjects of different conditions, the median value across channels was retained (Figure 4).*

*Finally, given the well-known impact of age not only on spectral exponent but also on slow delta oscillations (Kurth et al., 2010; Castelnovo et al., 2023; Bergamo et al., 2024), we conducted supplementary analyses to control the effects of age on our findings. These analyses corroborated the main results on slow wave properties of the disconnected cortex (Supplementary Material, section S12; Supplementary Figure 15). Moreover, given that in sleep the hotspot with maximal slow wave activity migrates across the developmental age from posterior to anterior regions in healthy children (Kurth et al., 2010), we performed supplementary analyses considering a subset of electrodes in the anterior and posterior regions, in order to verify potential regional effects (Supplementary Material, section S13; Supplementary Figures 16 and 17)*
